# Impact of health behaviours and deprivation on well-being in a national sample of English young people

**DOI:** 10.1136/bmjpo-2018-000335

**Published:** 2018-11-09

**Authors:** Aswathikutty Gireesh, Shikta Das, Russell M Viner

**Affiliations:** 1 Population, Policy and Practice Programme, UCL Great Ormond Street Institute of Child Health, London, UK; 2 MRC Unit for Lifelong Health and Ageing at UCL, UCL Institute of Cardiovascular Science, London, UK

**Keywords:** adolescent health, sleep, nutrition

## Abstract

**Objective:**

To determine the modifiable factors influencing well-being in boys and girls by accounting for deprivation, ethnicity and clustering within local authorities.

**Methods:**

We used data from a very large nationally representative survey, the What About Youth study involving 120 115 adolescents aged 15 years. Our outcome measure of mental well-being was the Warwick-Edinburgh Mental Wellbeing Scale (WEMWBS). Potential explanatory factors included substance abuse, screen time, eating habits, reading, bullying, sleeping pattern, physical activity and area-level deprivation. We ran unadjusted and adjusted multilevel models for each explanatory factor, after adjusting for ethnicity, deprivation and including a random effect for the local authority.

**Results:**

Boys had a higher overall mean WEMWBS score than girls (p<0.0001). In the adjusted model, each of multiple risk behaviours, eating habits, sleep, bullying, physical activity, screen-time and reading were independently associated with mental well-being in both boy and girls (p<0.0001 for both). Sleep and eating behaviours had a stronger association in both sexes than bullying, physical activity and screen time. Young people from black ethnic groups had significantly higher well-being in both sexes. Deprivation was not associated with well-being among boys but was among girls.

**Conclusion:**

The largest contributors to adolescent well-being appear to be sleep, eating behaviours and bullying when considered in a multivariable framework. While adolescents from black ethnic groups had higher overall well-being scores, area deprivation did not affect male well-being but had a small effect on female well-being. Future longitudinal studies and health policies need to consider a range of behavioural factors to drive improvements in adolescent well-being.

**What is already known on this topic?**

- Young people’s well-being may be affected by multiple individual and contextual factors.
- Key determinants of adolescent well-being remain unclear.
- Few studies have examined a wide range of potential determinants while adjusting for area-level deprivation.

**What this study hopes to add?**

- Findings support current policy foci on bullying, physical activity and screen-time as correlates of well-being among young people.
- Sleep and eating behaviours may also be important policy targets for promoting adolescent well-being.
- A coherent policy framework to promote adolescent well-being needs to be multifaceted and consider a range of health factors in young people’s lives.

## Introduction

There are growing concerns about the well-being of young people in modern societies, particularly in the UK where there is evidence that young people’s well-being is lower than in many comparable developed countries.^1 2^

Well-being is defined as ‘the state of being comfortable, healthy or happy’.^3^ From a holistic perspective, well-being incorporates different dimensions of adolescent lives including social relationships and individual functioning.^4^ However, the determinants of adolescent well-being is a relatively understudied area in comparison with the large literature on factors associated with mental health problems, as well-being concept has only been on greater focus over the past two decades.^5 6^

A wide range of factors have been shown to be related to adolescent well-being, including a range of cognitive and relational factors such as bullying,^7^ family structure and relationships,^8^ peer support^9^ and school connectedness.^10^ Other behaviours also influence well-being, including substance use (alcohol, drugs and smoking habits),^11–13^ fruit and vegetable consumption,^14^ breakfast consumption,^15^ physical activity,^16^ sleep duration,^17^ sedentary behaviour^7 18^ and leisure time activities.^19^ However, published studies use a wide range of well-being measures, resulting in conflicting findings.^20 21^ Furthermore, studies have largely focused on single risk factors and not explored how factors including behavioural factors interact to influence well-being. Additionally, given that many such behaviours are strongly socially patterned, studies have thus far paid little attention to confounding by socioeconomic position and issues relating to the clustering of behaviours and well-being within localities.

Policy initiatives to improve well-being among young people have largely focused on cognitive and psychological factors related to resilience to adversity, and have paid little attention to the contribution of non-psychological modifiable factors, such as other lifestyle behaviours. Understanding the potential contribution of modifiable behavioural factors to adolescent well-being may inform different strategies to improve young people’s well-being.

We used a very large recent nationally representative and population-based survey of English adolescents aged 15 years to examine the contribution of individual-level modifiable behaviours to well-being, including potentially protective (sleep, reading and physical activity) and risk behaviours (substance use, unhealthy eating habits and excessive screen time). Our objective was to identify modifiable behavioural factors for mental well-being in boys and girls using an adolescent-specific measure and accounting for deprivation, ethnicity and clustering within local authorities (LAs).

## Methods

### Study design and sample

The What About Youth study is a large-scale youth-oriented survey funded by the Department of Health in England and carried out by NHS Digital in 2014.^22^ The primary aim of the survey was to collect robust LA-level data on youth health behaviours and general health to improve their health outcomes. Study participants were those who turned 15 years old in the academic year 2013/2014. A random sampling methodology was employed to draw 298 080 participants from the National Pupil Database. The sample size was calculated to attain 1000 young people in each of 152 LAs in England; two LAs were merged with their nearest neighbours due to small size.

The achieved sample was 120 115 individuals, of whom 16% responded online and 84% via postal means (2835 opted out). The response rates differed by gender, with adjusted response rates of 35% in boys and 49% in girls, and by deprivation, ethnicity and LA. Non-response weights using these factors were calculated to provide alignment between the achieved and target samples.^22^ We obtained a fully anonymised cohort data electronically from the UK Data Service website.^22^

### Outcome variable

Mental well-being was measured using the Warwick-Edinburgh Mental Wellbeing Scale,^23^ a population-level well-being measure. It is validated to use in adolescents aged 13 years or more and focus primarily on the positive aspects of mental well-being (internal consistency, α=0.90). Participants indicate how often they feel like each of the 14 items using a 5-point scale that ranges from 5 ‘all the time’ to 1 ‘none of the time’.^24^ Total scores ranged from 14 to 70 and were calculated by summing each participant’s responses. The potential explanatory behavioural variables were identified from the literature review of previous publication. A detailed description of each variable is given in supplementary appendix A.

10.1136/bmjpo-2018-000335.supp1 Supplementary file 1

A composite variable for risk behaviour index for substance use was constructed by the summation of three dichotomous risk behaviour variables: (a) smoking: if currently smokes, (b) drinking alcohol: if drinks once a month or more frequently, (c) cannabis use: if ever tried cannabis. Based on the number of risk behaviours, we categorised it from ‘none’ to ‘three’. Similarly, composite unhealthy eating habit index was derived from (a) skipping breakfast: if avoided breakfast in last 7 days, (b) poor diet: if consumed less than five portions of fruits and vegetables a day, (c) takeaway food: if consumed takeaway food in past 7 days. Based on a combination of unhealthy behaviours, the composite score was categorised into 0=none, 1=only one, 2=any two and 3=all three. Physical active for 60+ min for at least 5 days were classified as ‘physically active’ and the rest ‘physically inactive.’ This threshold was defined in line with government recommendations,^25 26^ except for the intensity of exercise which was not available in the dataset. The selected threshold was taken at 5 days a week, as only 13% reported being physically active for 7 days a week. A digital screen time variable was computed based on reported weekend and weekday usage of television, internet, smartphone and computer games. Subjects were categorised into ‘≥7 hours/day’, ‘about 5–6 hours/day’, ‘about 3–4 hours/day’, ‘about 2 hours/day’ and ‘≤1 hours/day’. Time spent reading on weekends and weekdays had response options ranging from none to 7 hours per day. Based on the distribution of data, we recoded the variable as ‘none’, ‘about half an hour/day’, ‘about 1 hour/day’ and ‘2 hours /day’. The frequency of 8 hours sleep in the last 7 days was coded as ‘every day’, ‘most days’, ‘some days’ and ‘not in the past 7 days’. Bullying was measured with Olweus Bully/Victim Questionnaire, a reliable 8-item scale used to assess the bullying victimisation.^27^ We combined responses to create one overall measure of bullying experience (yes/no). In line with a previous study,^28^ adolescents who were bullied more than ‘two or three times a month’ were categorised as bullying victims.

Ethnicity, deprivation and mode of questionnaire completion were selected as confounders in the relationship between well-being and potential explanatory variables as shown in previous studies.^7^ Ethnicity was self-identified by participants’ and was an adaptation of the 2001 UK census categories, supplemented by questions on the national group. English Index of Multiple Deprivation (IMD) was used as a measure of relative deprivation for small areas.^29^ IMD scores were divided into three deprivation categories as defined by quintiles of the national distribution: 1 and 2 (high deprivation), 3 (average), 4 and 5 (low). Participants were allowed to choose between online or postal modes of questionnaire completion.

### Analyses

We conducted unadjusted and adjusted multilevel regression, in Stata V.14. For well-being scores, the interaction between gender and health behaviours was statistically significant (p<0.001) and therefore, analyses were stratified by gender. All variables were plotted to check the distribution and normality was checked with the Kolmogorov-Smirnov test. All estimates were weighted by representativeness of participants to compensate for the disproportionate selection of sample and non-response bias. Pearson χ^2^ tests were used to compare differences in the distribution of explanatory variables by gender. An unadjusted analysis was run to test the association between each independent variable (substance use, unhealthy eating habits, screen time, reading, bullying, physical activity and sleeping hours) and the outcome adolescent well-being (model 1). The analyses were repeated in a multivariable analysis where ethnicity, mode of questionnaire delivery and IMD were added as confounders between each risk factor and outcome (model 2). In model 3, all explanatory factors and covariates were included simultaneously to obtain associations between each variable and well-being scores after adjusting for confounders and other explanatory variables. LAs were treated as random effects in all models. Models were also tested for significant quadratic terms signifying curvilinear relationships; this was only significant for reading. The intraclass correlations coefficient, being the proportion of total variance attributable to differences at the LA level, was estimated using multilevel models for well-being with adjustment for IMD, ethnicity and mode of completion.

## Results

In total, 57 153 boys (47.82%) and 62 962 girls (52.18%) participated in the study (table 1). Of these, boys had higher average well-being score in comparison with girls. The intracluster correlation coefficient for the well-being score was 0.032 for girls and 0.024 for boys in the adjusted model, suggesting that variance in adolescent well-being is small at LA level.

**Table 1:** Descriptive statistics for well-being scores and explanatory variables under study, by gender

	Total	Boys		Girls		
	N	N	Mean (SD)/%	N	Mean (SD)/%	P values
Full sample (N %)	**120 115**	**57 153**	**47.58%**	**62 962**	**52.42%**	
WEMWBS scores	117 842	56 352	47.82%	61 490	52.18%	<0.0001
Mean (SD)			50 (8.60)		45 (9.66)	
Substance use*	71 133	32 516	45.71%	38 617	54.29%	<0.0001
None		17 133	52.69	19 932	51.61	
One		10 783	33.16	12 150	31.46	
Two		3010	9.26	3785	9.8	
Three		1590	4.89	2750	7.12	
Unhealthy eating habits†	115 918	55 289	47.70%	60 629	52.30%	<0.0001
None		12 081	21.85	11 951	19.71	
One		20 287	36.69	20 389	33.63	
Two		16 373	29.61	19 064	31.44	
Three		6548	11.84	9225	15.22	
Sleeping hours (>8 hours)	117 516	56, 207	47.83%	61 307	52.17%	<0.0001
Not in the past 7 days		3676	6.54	6922	11.29	
Some days		11 051	19.66	16 051	26.18	
Most days		20, 121	35.8	20 928	34.14	
Everyday		21, 361	38	17 406	28.39	
Bullying	117 744	56 309	47.82%	61 435	52.18%	<0.0001
No		45 959	81.62	45 094	73.40	
yes		10 350	18.38	16 341	26.60	
Physical activity	118 450	56 674	47.85	61 776	52.15	<0.0001
Physically active		48 172	85	44 348	71.79	
Inactive		8502	15	17 428	28.21	
Screen time	118 845	56 892	47.87	61 943	52.13%	<0.0001
About 2 hours/day		3609	6.34	3469	5.6	
≤1 hours/day		912	1.6	929	1.5	
About 3–4 hours/day		18 621	32.73	17 311	27.95	
About 5–6 hours/day		17 198	30.23	17 850	28.82	
≥7 hours/day		16 559	29.1	22 387	36.14	
Reading	118 140	56 513	47.84	61 627	52.16	<0.0001
None		14 278	25.26	9875	16.02	
About Half an hour/day		17 572	31.09	15 363	24.93	
About 1 hour/day		12 505	22.13	13 442	21.81	
≥ 2 hours/day		12 158	21.51	22947	37.24	

Figures in bold refer to full sample distribution, the proportion of males and females in the study.

*Risk behaviours include smoking, drinking and cannabis use.

†Unhealthy eating habits include skipping breakfast, not having five portions of fruits and vegetables and consumption of takeaway food.

WEMWBS, Warwick-Edinburgh Mental Wellbeing score.

Table 1 shows that there were significant differences in the distribution of potential explanatory variables between boys and girls. Girls had higher risk factors such as substance use, unhealthy eating habits, screen time and reported more bullying than boys. Protective variables such as sleeping hours (more than 8 hours) and reading were also significantly higher in girls than in boys.

All explanatory variables and IMD were significantly associated with well-being in both sexes (table 2). Well-being in both sexes decreased with the use of substance use, unhealthy eating habits, bullying, physical activity and longer screen time in both sexes. Protective factors, such as, sleeping more than 8 hours and reading more than 2 hours were associated with higher well-being in both sexes.

**Table 2:** Univariate analysis between well-being and explanatory variables, by gender

	Model†			
	Boys		Girls	
	B	95 % CI	B	95 % CI
Substance use				
None	Reference		Reference	
One	−0.34*	(−0.56 to –0.13)	−1.48**	(−1.70 to –1.27)
Two	−2.19**	(−2.60 to –1.78)	−4.96**	(−5.35 to –4.57)
Three	−3.56**	(−4.16 to –2.95)	−5.89**	(−6.47 to –5.30)
Unhealthy eating habits				
None	Reference		Reference	
One	−1.65**	(−1.88 to –1.43)	−2.63**	(−2.91 to –2.34)
Two	−3.36**	(−3.60 to –3.12)	−4.85**	(−5.13 to –4.58)
Three	−5.00**	(−5.39 to –4.61)	−6.49**	(−6.83 to –6.14)
Sleeping hours (>8 hours)				
Not in the past 7 days	Reference		Reference	
Some days	2.99**	(2.51 to 3.47)	4.71**	(4.39 to 5.04)
Most days	5.59**	(5.15 to 6.03)	8.39**	(8.06 to 8.72)
Everyday	7.49**	(7.07 to 7.87)	10.70**	(10.35 to 11.06)
Bullying				
No	Reference		Reference	
Yes	−4.70**	(−3.59 to –3.31)	−5.84**	(−5.25 to –4.90)
Physical activity				
Physically active	Reference		Reference	
Inactive	−3.85**	(−4.11 to –3.61)	−2.77**	(−3.04 to –2.51)
Screen time				
About 2 hours/day	Reference		Reference	
≤1 hours/day	0.07	(−0.59 to 0.73)	0.08	(−0.69 to 0.85)
About 3–4 hours/day	−1.22**	(−1.60 to –0.84)	−1.27**	(−1.71 to –0.83)
About 5–6 hours/day	−2.17**	(−2.51 to –1.83)	−3.06**	(−3.52 to –2.60)
≥7 hours/day	−3.72**	(−4.12 to –3.32)	−5.38**	(−5.80 to –4.97)
Reading				
None	Reference		Reference	
About Half an hour/day	1.61**	(1.41 to 1.82)	2.17**	(1.88 to 2.46)
About 1 hour/day	2.25**	(2.06 to 2.44)	2.54**	(2.26 to 2.82)
≥2 hours/day	2.27***	(2.01 to 2.52)	2.35**	(2.05 to 2.66)
IMD scores				
High deprivation	Reference		Reference	
Average deprivation	0.57**	(0.31 to 0.81)	0.35**	(0.10 to 0.590
Least deprivation	1.05**	(0.87 to 1.21)	1.16**	(0.92 to 1.40)
Ethnicity				
White	Reference		Reference	
Mixed	0.18	(−0.29 to 0.65)	−0.44*	(−0.82 to –0.06)
Asian	−0.54*	(−0.85 to –0.23)	0.98**	(0.59 to 1.38)
Black	0.91**	(0.42 to 1.39)	1.10**	(0.67 to 1.52)
Other	−0.45*	(−0.84 to –0.04)	0.30	(−0.09 to 0.70)

*P<0.05; **P<0.001.

†Unadjusted model taking into account clustering at local authority level, multilevel models fitted with weighted design weights, quadratic function added to reading.

IMD, Index of Multiple Deprivation.

In the multivariable models adjusted for covariates (table 3), poorer well-being was associated with multiple substances use and multiple unhealthy eating habits in a dose-dependent fashion. Being physically inactive, longer screen time and experiencing bullying were both associated with decrements in well-being in both sexes, with the association being stronger in girls than in boys.

**Table 3:** Gender-stratified partially adjusted and fully adjusted multilevel modelling for well-being and explanatory variables

Variables	Boys				Girls			
	Model†		Model‡		Model†		Model‡	
	B	95 % CI	B	95 % CI	B	95 % CI	B	95 % CI
Substance use								
None	Reference		Reference		Reference		Reference	
One	−0.40**	(−0.61 to –0.18)	−0.14	(−0.36 to 0.08]	−1.51**	(−1.73 to –1.30)	−0.77**	(−0.97 to –0.57)
Two	−2.16**	(−2.57 to –1.75)	−1.05**	(−1.42 to –0.67)	−4.84**	(−5.22 to –4.46)	−2.67**	(−3.01 to –2.33)
Three	−3.50**	(−4.11 to –2.88)	−1.63**	(−2.16 to –1.09)	−5.80**	(−6.36 to –5.23)	−2.79**	(−3.35 to –2.24)
Eating habits								
None	Reference		Reference		Reference		Reference	
One	−1.63**	(−1.85 to –1.41)	−0.89**	(−1.18 to –0.60)	−2.63**	(−2.92 to –2.34)	−1.37**	(−1.69 to –1.06)
Two	−3.31**	(−3.55 to –3.08)	−1.84**	(−2.14 to –1.54)	−4.84**	(−5.12 to –4.55)	−2.29**	(−2.64 to –1.96)
Three	−4.95**	(−5.34 to –4.56)	−2.44**	(−2.82 to –2.06)	−6.46**	(−6.79 to –6.10)	−2.61**	(−3.04 to –2.18)
Sleeping hours (>8 hours)								
Not in the past 7 days	Reference		Reference		Reference		Reference	
Some days	2.98**	(2.50 to 3.45]	2.69**	(2.10 to 3.28)	4.69**	(4.36 to 5.01)	3.71**	(3.34 to 4.08)
Most days	5.55**	(5.11 to 5.99]	4.30**	(3.78 to 4.82)	8.35**	(8.01 to 8.69)	6.64**	(6.28 to 7.00)
Everyday	7.45**	(7.03 to 7.86]	5.79**	(5.29 to 6.28)	10.65**	(10.29 to ,11.01)	8.16**	(7.72 to 8.60)
Bullying								
No	Reference		Reference		Reference		Reference	
Yes	−4.64**	(−4.86 to –4.42)	−3.78**	(−4.09 to –3.48)	−5.77**	(−5.98 to –5.56)	−4.01**	(−4.23 to –3.78)
Physical activity								
Physically active	Reference		Reference		Reference		Reference	
Inactive	−3.78**	(−4.02 to –3.55)	−2.63**	(−2.95 to –2.30)	−2.77**	(−3.04 to –2.50)	−1.70**	(−2.01 to –1.39)
Screen time								
About 2 hours/day	Reference		Reference		Reference		Reference	
≤1 hours/day	0.09	(−0.57 to 0.75]	0.34	(−0.58 to 1.26)	0.09	(−0.69 to 0.87)	−0.38	(−1.49 to 0.72)
About 3–4 hours/day	−1.22**	(−1.60 to –0.83)	−0.61*	(−0.99 to –0.23)	−1.26**	(−1.70 to –0.83)	−0.54	(−1.14 to 0.05)
About 5–6 hours/day	−2.15**	(− 2.50 to – 1.81)	−0.82**	(−1.27 to –0.37)	−3.04**	(−3.49 to –2.58)	−1.21**	(−1.75 to ,–0.67)
≥7 hours/day	−3.67**	(−4.06 to –3.27)	−1.20**	(−1.65 to –0.75)	−5.32**	(−5.73 to –4.90)	−1.81**	(−2.29 to –1.33)
Reading								
None	Reference		Reference		Reference		Reference	
About Half an hour/day	1.59**	(1.38 to 1.80)	0.57**	(0.26 to 0.88)	2.13**	(1.84 to 2.42)	0.56**	(0.23 to 0.90)
About 1 hour/day	2.24**	(2.04 to 2.43)	1.04**	(0.79 to 1.29)	2.51**	(2.24 to 2.78)	0.60**	(0.26 to 0.93)
≥2 hours/day	2.28**	(2.05 to 2.54)	0.90**	(0.55 to 1.25)	2.34**	(2.03 to 2.64)	0.33*	(0.02 to 0.64)
IMD scores								
High deprivation	–	–	Reference		–	–	Reference	
Average deprivation	–	–	−0.12	(−0.38 to 0.14)	–	–	0.16	(−0.16 to 0.49)
Least deprivation	–	–	0.17	(−0.06 to 0.40)	–	–	0.36*	(0.06 to 0.66)
Ethnicity								
White	–	–	Reference		–	–	Reference	
Mixed	–	–	−0.06	(0.81 to –0.59)	–	–	0.44	(−0.08 to 0.96)
Asian	–	–	−0.09	(0.81 to –0.84)	–	–	0.35	(−0.53 to 1.23)
Black	–	–	0.99*	(0.01 to 0.25)	–	–	1.75**	(1.20 to 2.31)
Other	–	–	−0.59	(0.02 to –1.09)	–	–	−0.19	(−0.88 to 0.49)
Mode of questionnaire delivery								
Online	–	–			–	–		
paper	–	–	0.24	(−0.03 to 0.51)	–	–	1.26**	(0.99 to 1.53)

Multilevel models fitted with weighted design weights, quadratic function added to reading.

*P<0.05; P<0.005.

†Each predictor variable adjusted for ethnicity, mode of questionnaire delivery and IMD.

‡ All variables mutually adjusted for each other.

IMD, Index of Multiple Deprivation.

**Figure 1:**
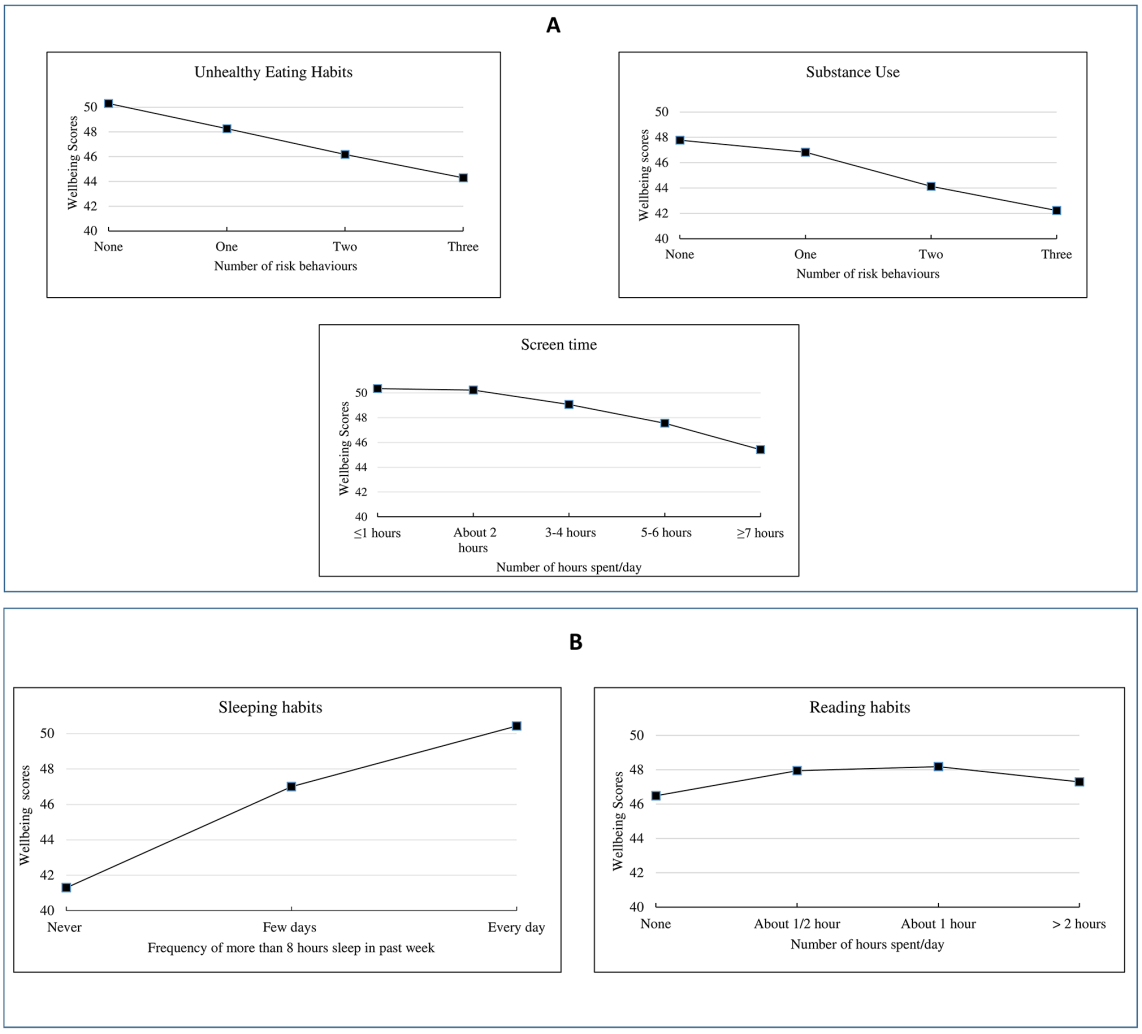
Relationship between well-being and health behaviours: (A) risk factors, (B) protective factors.

Higher well-being was associated with the number of days young people achieved more than 8 hours of sleep, again in a dose-dependent fashion. Habitual reading most days was associated with higher well-being although there was no evidence of a dose–response above 30 min per day in the fully adjusted model (figure 1). Adolescents from black ethnic groups had higher well-being scores overall. Area deprivation did not affect male well-being but had a small effect on female well-being.

## Discussion

This study broadens our understanding of risk and protective factors associated with well-being in adolescence, using a very large nationally representative survey to examine a wide spectrum of behavioural and psychosocial factors relating to youth well-being and taking into account deprivation and clustering at LA level. The study shows that young people who reported lower levels of well-being were more likely to have engaged in multiple unhealthy eating habits and substance use, be victims of bullying, have exercised insufficiently, have exceeded recommended screen time use. These findings were robust to mutual adjustment for all variables and for deprivation, ethnicity and mode of questionnaire delivery. A dose–response pattern was also observed between well-being and health behaviours such as substance use, unhealthy eating habits and sleeping pattern. A decrease in the number of risk behaviours and an increase in the number of days slept for more than 8 hours, corresponded with an increase in average levels of well-being. The impact of deprivation on well-being was surprisingly small, as was LA locality variance, suggesting that variance in well-being lies largely in behavioural and psychological factors.

Since different studies have used different variables to define various aspects of well-being in the analyses, comparison with other studies is difficult. We found boys reported higher mean well-being than girls, consistent with national reports for England,^30 31^ but in contrast to findings from recent Health Survey England 2015, where only slight gender variations were observed.^32^ The proportions reporting each of the behaviours were broadly similar to those found in other recent national surveys.^31 33 34^ Girls reported higher levels of risky health behaviours including current smoking, alcohol consumption, bullying and lower levels of physical activity. On the other hand, boys were more likely to report higher levels of physical activity, and these were consistent with findings from the Health Behaviour in School-aged Children England.^33^

These findings corroborate with two previous studies where happiness as a marker of well-being was found to be positively associated with multiple health protective behaviours (sports participation and healthier eating) and negatively associated with multiple risk behaviours (smoking, alcohol use and heavy screen use) in adolescents.^35 36^ Our finding that substance use was associated with lower well-being is similar to that seen in other studies, as were our findings for being bullied.^7^ The association of sleep duration and reading with well-being in young people has been little studied. Leisure time and adequate sleep have been identified as being associated with well-being,^17 19^ however ours is the first to examine these alongside other behavioural and psychological factors.

We found an association between deprivation and lower well-being, although in contrast to previous studies^37^ the association was small, and we found no association in boys. This may reflect the lack of adjustment for multiple behaviours, ethnicity and area effects in other studies. Our finding suggests that much of what has previously been understood as unhealthy behaviours themselves associated with deprivation may mediate deprivation effects. We found that young people from black ethnic groups reported significantly higher well-being in both sexes, consistent with previous UK findings.^38^ However, the reasons for this remain unclear and require further study. We found this association to be robust to adjustment for deprivation and all significant behavioural and psychological factors, suggesting this likely relates to factors not measured in our study.

We used a large, nationally representative sample of ethnically and socioeconomically diverse adolescents. Prior studies have examined very few health behaviours and relied on proxy measures of well-being rather than on population-level well-being measures that tap into both feelings and psychological flourishing. In our study, associations between well-being and behavioural factors were examined within a multivariable and multilevel framework, using a validated well-being scale with robust psychometric properties.

Our findings are subject to a number of limitations. Our data were cross-sectional and thus the direction of causality is unclear for the behavioural variables. Participant responses could be influenced by social desirability, and those with poor well-being may be inclined toward endorsing questions more than others, thus introducing bias. The direction of such biases is unclear, however, we note that girls (who had a higher response rate in the overall survey) reported higher levels of both more risky and protective behaviours than boys, potentially reflecting social desirability biases. All variables used were self-reported except for area-level deprivation. We have also repeated analysis excluding the outliers and that did not materially affect the findings. We used bullying victimisation as a proxy for psychological problems due to the lack of more appropriate variables in the dataset; thus it is possible that some of the associations seen here result from inadequate adjustment for psychological issues. We combined variables across domains into composite variables. this may have introduced bias although the direction of bias is unclear.

## Conclusion

Our findings suggest that promoting healthy sleep, reading and healthy eating behaviours may present important policy targets for enhancing adolescent well-being in addition to more accepted foci on physical activity, screen time and bullying. While there was an association between deprivation and well-being, the association was small. Future work is needed to examine these modifiable factors within a longitudinal causal framework.

## Supplementary Material

Author's manuscript
