# Supplementary material for: Impact of health behaviours and deprivation on well-being in a national sample of English young people
Source: BMJ Paediatr Open. 2018 Nov 9;2(1):e000335. doi: 10.1136/bmjpo-2018-000335 (PMC6241972; doi:10.1136/bmjpo-2018-000335)
Supplement: Supplementary file 1 [file bmjpo-2018-000335supp001.pdf]

APPENDIX A

(i) VARIABLE DESCRIPTION

Composite variables	Original variables	Variable labels	Composite variables
**Substance use risk** [1-6]	**Definitions were:**		**Alcohol + smoking + cannabis**
	Smoking: Cigarette smoking status (currently smoke)	SmkSt2	(1) Smoking risk: If currently smokes (2) Drinking risk: If drinks once a month or more frequently (3) Cannabis risk: If ever tried cannabis. Each item was coded 0 for "no" and 1 for "yes". The composite score obtained ranged from zero to three based on the number of risk behaviours; (0= None, 1= Only one, 2 =Any two and 3 =All three
	Drinking: Alcohol drinking status (Usually have an alcoholic drink once a month or more frequently)	alcfrq1	
	Cannabis: Cannabis use status (Ever tried cannabis)	Cantry	
**Physical Activity** [7 8]	**Physical activity: in line with general recommendations**		**Duration of exercise + Frequency of exercise + Number of days met recommended guidelines**
	How many days physically active for 60+ minutes, in last 7 days (Active for more than 5 days)	Physact	The first question on moderate to vigorous physical activity was "Over the past 7 days, on how many days you were physically active for a total of at least 60 minutes per day and responses ranged from 0 to 7. Second, the participants were asked "Outside school hours: how often do you usually exercise in your free time so much that you get out of breath or sweat?" with responses from less than a once a month to every day. The question on duration was "Outside school hours: how many hours do you usually exercise in your free time so much that you get out of breath or sweat?", with responses from none to 7 or more hours. Adolescents who exercised for more than twice a week for an hour or more or were physically active for 60+ minutes for at least 5 days were classified as "physically active" and the rest "physically inactive".
	How often usually exercise in free time (Active for more than twice a week)	ExerOft	
	How many hours usually exercise in free (Active for an hour or more)	ExerHrs	
**Sleeping habits** [9 10]	**Sleeping frequency in last week**		
	How often slept 8+ hours per night in last 7 days	Sleep8pn	The question was "Over the past 7 days, how often did you sleep for 8 hours or more" and the responses were "every day", "most days", "some days" and "not in the past 7 days".

**Eating Habits** [11-13]	**Definition of each unhealthy eating habits were:**		**Breakfast consumption + take away food + 5 A day**
	How often eaten take-away food in last 7 days (unhealthy habit if taken takeaway in past seven days)<br>How often eaten breakfast in last 7 days (unhealthy habit if breakfast not taken regularly)<br>Consumed more than five portions of fruit and veg yesterday (unhealthy habit if 5-A-DAY not taken regularly)	Eattake<br><br>Eatbfst<br><br>FV5ormor	(1) Skipping Breakfast: If avoided breakfast in last 7 days (2) Poor diet: If consumed less than 5 portions of fruits and vegetables a day (3) Takeaway food: If consumed takeaway food in past 7 days. Each item was coded 0 for "no" and 1 for "yes". According to the number of unhealthy eating habits participant's exhibited, a risk score from zero to three (0= None, 1= Only one, 2 =Any two and 3 =All three) was given
**Screen time** [8 14-16]	**Definitions of screen time derived as follows (Weekend and weekday hours combined)**		**TV +_computer games + internet**
	Hours per day watching TV<br><br>Hours per day spent playing computer games<br><br>Hours per day spent on computers for communicating, or homework<br>Hours per day spent on smart phone	Watchwk, Watchwe<br>Compwk , Compwe<br>Comphwk, Comphwe<br>Smartwk, Smartwe	FOR ALL, Response options for each item were 'none at all', 'about half an hour a day', 'about 1 hour a day', 'about 2 hours a day', 'about 3 hours a day', 'about 4 hours a day', 'about 5 hours a day', 'about 6 hours a day' and 'about 7 or more hours a day'. The total screen time was calculated by grouping the similar options of each item and by combining both weekends and weekdays. Subjects were then categorised into "≥7hrs/day", "About 5-6 hours/day", "About 3 -4 hours/day", "About 2 hrs/day" and "≤1hrs/day".
**Reading** [17]	**Definitions of reading derived as follows:**		**Reading hours (Weekend + weekdays)**
	Hours per day spent reading outside school hours	Readwk, Readwe	"About how many hours a day do you usually spend sitting down reading books, magazines or newspapers (including e-readers, online newspapers, and magazines) and studying when you are not at school?". The questions were asked for weekends and weekdays separately. The response options ranged from none to ≥ 7 hours per day
**Bullying** [18 19]	**Frequency with which the behaviour occurred over the past couple of months:**		**Traditional bullying (physical+ verbal + relational)**

I was called mean names, was made fun of, or teased in a hurtful way	Bllynam	Bullying measure was meant to assess the extent to which they had recently experienced other children directing different types of bullying towards themselves. Six statements reflecting traditional bullying (physical, verbal, and relational) were asked. Questions were rated by participants using a 5-point response scale that ranged from 0="I haven't been bullied this way in the past couple of months" 1="It has happened once or twice", 2="2 or 3 times a month", 3="2 or 3 times a week", to 4="Several times a week."
Other people left me out of things on purpose, excluded me from their group of friends, or completely ignored me	Bllyout	
I was hit, kicked, pushed, shoved around, or locked indoors"	Bllyhit	
Other people lied or spread humours	Bllylie	
Made fun of me because of weight	Bllyfun	
Sexual jokes/comments	Bllysex	

(ii) Individual Items and scoring of Warwick-Edinburgh Mental Well-being scale (WEMWBS)

1. I've been feeling optimistic about the future
2. I've been feeling useful
3. I've been feeling relaxed
4. I've been feeling interested in other people
5. I've had energy to spare
6. I've been dealing with problems well
7. I've been thinking clearly
8. I've been feeling good about myself
9. I've been feeling close to other people
10. I've been feeling confident
11. I've been able to make up my own mind about things
12. I've been feeling loved
13. I've been interested in new things
14. I've been feeling cheerful

Each of the 14 item responses in WEMWBS are scored from 1 (none of the time), 2 (rarely), 3 (some of the time), 4 (often) to 5 (all of the time). A total scale score is calculated by summing the 14 individual item scores.

REFERENCES

1. Farmer S, Hanratty B. The relationship between subjective wellbeing, low income and substance use among schoolchildren in the north west of England: a cross-sectional study. *Journal of Public Health* 2012;34(4):512-22. doi: 10.1093/pubmed/fds022
2. Phillips-Howard PA, Bellis MA, Briant LB, et al. Wellbeing, alcohol use and sexual activity in young teenagers: findings from a cross-sectional survey in school children in North West England. *Substance abuse treatment, prevention, and policy* 2010;5:27. doi: 10.1186/1747-597x-5-27 [published Online First: 2010/11/12]
3. Newcomb MD, Bentler PM, Collins C. Alcohol Use and Dissatisfaction with Self and Life: A Longitudinal Analysis of Young Adults. *Journal of Drug Issues* 1986;16(4):479-94. doi: 10.1177/002204268601600401
4. Bergman MM, Scott J. Young adolescents' wellbeing and health-risk behaviours: gender and socio-economic differences. *Journal of Adolescence* 2001;24(2):183-97. doi: https://doi.org/10.1006/jado.2001.0378
5. Taylor G, McNeill A, Girling A, et al. Change in mental health after smoking cessation: systematic review and meta-analysis. *BMJ : British Medical Journal* 2014;348 doi: 10.1136/bmj.g1151
6. Booker CL, Skew AJ, Sacker A, et al. Well-Being in Adolescence—An Association With Health-Related Behaviors:Findings From Understanding Society, the UK Household Longitudinal Study. *The Journal of Early Adolescence* 2014;34(4):518-38. doi: 10.1177/0272431613501082
7. Ussher MH, Owen CG, Cook DG, et al. The relationship between physical activity, sedentary behaviour and psychological wellbeing among adolescents. *Social Psychiatry and Psychiatric Epidemiology* 2007;42(10):851-56. doi: 10.1007/s00127-007-0232-x
8. Booker CL, Skew AJ, Kelly YJ, et al. Media Use, Sports Participation, and Well-Being in Adolescence: Cross-Sectional Findings From the UK Household Longitudinal Study. *American Journal of Public Health* 2015;105(1):173-79. doi: 10.2105/AJPH.2013.301783
9. Fuligni AJ, Hardway C. Daily Variation in Adolescents' Sleep, Activities, and Psychological Well-Being. *Journal of Research on Adolescence* 2006;16(3):353-78. doi: 10.1111/j.1532-7795.2006.00498.x
10. James S, Hale L. Sleep Duration and Child Wellbeing: A Nonlinear Association. *Journal of clinical child and adolescent psychology : the official journal for the Society of Clinical Child and Adolescent Psychology, American Psychological Association, Division 53* 2017;46(2):258-68. doi: 10.1080/15374416.2016.1204920
11. Rooney C, McKinley MC, Woodside JV. The potential role of fruit and vegetables in aspects of psychological well-being: a review of the literature and future directions. *Proceedings of the Nutrition Society* 2013;72(4):420-32. doi: 10.1017/S0029665113003388 [published Online First: 09/11]
12. Conner TS, Brookie KL, Carr AC, et al. Let them eat fruit! The effect of fruit and vegetable consumption on psychological well-being in young adults: A randomized controlled trial. *PLoS ONE* 2017;12(2):e0171206. doi: 10.1371/journal.pone.0171206
13. Lien L. Is breakfast consumption related to mental distress and academic performance in adolescents? *Public Health Nutrition* 2007;10(4):422-28. doi: 10.1017/S1368980007258550 [published Online First: 04/01]
14. Hoare E, Milton K, Foster C, et al. The associations between sedentary behaviour and mental health among adolescents: a systematic review. *International Journal of Behavioral Nutrition and Physical Activity* 2016;13(1):108. doi: 10.1186/s12966-016-0432-4
15. Przybylski AK, Weinstein N. A Large-Scale Test of the Goldilocks Hypothesis:Quantifying the Relations Between Digital-Screen Use and the Mental Well-Being of Adolescents. *Psychological Science* 2017;28(2):204-15. doi: 10.1177/0956797616678438

16. Hamer M, Yates T, Sherar LB, et al. Association of after school sedentary behaviour in adolescence with mental wellbeing in adulthood. *Preventive Medicine* 2016;87(Supplement C):6-10. doi: https://doi.org/10.1016/j.ypmed.2016.02.021
17. Trainor S, Delfabbro P, Anderson S, et al. Leisure activities and adolescent psychological well-being. *Journal of Adolescence* 2010;33(1):173-86. doi: https://doi.org/10.1016/j.adolescence.2009.03.013
18. Landstedt E, Persson S. Bullying, cyberbullying, and mental health in young people. *Scandinavian journal of public health* 2014;42(4):393-9. doi: 10.1177/1403494814525004 [published Online First: 2014/03/13]
19. Przybylski AK, Bowes L. Cyberbullying and adolescent well-being in England: a population-based cross-sectional study. *The Lancet Child & Adolescent Health* 2017;1(1):19-26. doi: 10.1016/S2352-4642(17)30011-1
