# Supplementary material for: Impact of health behaviours and deprivation on well-being in a national sample of English young people
Source: BMJ Paediatr Open. 2018 Nov 9;2(1):e000335. doi: 10.1136/bmjpo-2018-000335 (PMC6241972; doi:10.1136/bmjpo-2018-000335)
Supplement: Author's manuscript [file bmjpo-2018-000335.draft_revisions.pdf]

BMJ Paediatrics Open

BMJ Paediatrics Open is committed to open peer review. As part of this commitment we make the peer review history of every article we publish publicly available.

When an article is published we post the peer reviewers' comments and the authors' responses online. We also post the versions of the paper that were used during peer review. These are the versions that the peer review comments apply to.

The versions of the paper that follow are the versions that were submitted during the peer review process. They are not the versions of record or the final published versions. They should not be cited or distributed as the published version of this manuscript.

BMJ Paediatrics Open is an open access journal and the full, final, typeset and author-corrected version of record of the manuscript is available on our site with no access controls, subscription charges or pay-per-view fees (http://bmjpaedsopen.bmj.com).

If you have any questions on BMJ Paediatrics Open's open peer review process please email info.bmjpo@bmj.com

BMJ Paediatrics Open

The Impact of Health Behaviours and Deprivation on Wellbeing in a National Sample of English Young People

Journal:	*BMJ Paediatrics Open*
Manuscript ID	bmjpo-2018-000335
Article Type:	Original article
Date Submitted by the Author:	14-Jun-2018

Complete List of Authors:	Gireesh, Aswathikutty; University College London, Institute of Child Health<br>Das, Shikta; University College London, Institute of Child Health<br>Viner, Russell; UCL Institute of Child Health, Population, Policy & Practice Research Programme

Keywords:	Adolescent Health, Sleep, Nutrition

Confidential: For Review Only

SCHOLARONE™
Manuscripts

TITLE

The Impact of Health Behaviours and Deprivation on Wellbeing in a National Sample of English Young People

Aswathikutty Gireesh MSc. [a]*

Shikta Das PhD [b]*

Russell Viner M.B.B.S., PhD [a]

*Joint first authors

[a] UCL Great Ormond Street Institute of Child Health, Population, Policy and Practice Programme, 30 Guilford Street, London WC1N 1EH, United Kingdom

[b] UCL Institute of Cardiovascular Science, MRC Unit for Lifelong Health and Ageing at UCL

Address for correspondence:

Aswathikutty Gireesh

Great Ormond Street Institute of Child Health, Population, Policy and Practice Programme. 30, Guilford Street, London WC1N 1EH, UK.

Email address: a.aswathikutty-gireesh@ucl.ac.uk

Funding Sources

This report is independent research commissioned and funded by the National Institute for Health Research Policy Research Programme. The views expressed in this publication are those of the author(s) and not necessarily those of the NHS, the National Institute for Health

Confidential: For Review Only

Research, the Department of Health or its arm's length bodies, and other Government Departments.

Conflicts of interests

The authors have no conflicts of interest to disclose

Word count: 2574

Confidential: For Review Only

ABSTRACT

Objective: To determine the malleable factors influencing wellbeing in boys and girls by accounting for area-level deprivation, ethnicity and clustering within local authorities

Methods: We used data from a nationally-representative survey, the What About Youth (WAY) study involving 120,115 adolescents aged 15 years. Our outcome measure of mental wellbeing was the Warwick-Edinburgh Mental Wellbeing Scale (WEMWBS). The explanatory factors included potentially protective behaviours (sleep, reading and physical activity), risk behaviours (substance use, unhealthy eating habits and excessive screen time) and area-level deprivation. We ran unadjusted and adjusted multilevel models for each explanatory factor, adjusting for ethnicity, deprivation and including a random effect for a local authority (LA).

Results: Boys had a higher overall mean WEMWBS score than girls (*P* <0.0001). In the adjusted model, each of multiple risk behaviours, eating habits, sleep, bullying, physical activity, screen-time and reading were independently associated with mental wellbeing in both boy and girls (P<0.0001 for both). Sleep and eating behaviours had a stronger effect in both sexes than bullying, physical activity and screen time.

Conclusion: The largest contributors to adolescent wellbeing appear to be sleep, eating behaviours and bullying when considered in a multivariable framework. Future longitudinal studies and health policies need to consider range of behavioural factors to drive improve improvements in adolescent wellbeing.

Keywords: Adolescent, Young People, Mental Wellbeing, Health Behaviours

Confidential: For Review Only

There are growing concerns about the wellbeing of young people in modern societies particularly in the UK where there is evidence that young people's wellbeing is lower than in many comparable developed countries [1-3]. Wellbeing is defined as “the state of being comfortable, healthy or happy” [4]. From a holistic perspective, wellbeing incorporates different dimensions of adolescent lives including social relationships as well as individual functioning [5]. However, the determinants of adolescent wellbeing is a relatively understudied area in comparison with the large literature on factors associated with mental health problems, as wellbeing concept has only been on greater focus over the past two decades [6 7]. A wide range of factors have been shown to be related to adolescent wellbeing, including a range cognitive and relational factors such as bullying [8] family structure and relationships [9], peer support [10] and school connectedness[11] . Other behaviours also influence wellbeing, including substance use (alcohol, drugs and smoking habits) [12-14], fruit and vegetable consumption [15 16], breakfast consumption [17], physical activity [18], sleep duration [19], sedentary behaviour [20 21] and leisure time activities[22]. However, published studies use a wide range of wellbeing measures, resulting in conflicting findings [23-26]. Furthermore, studies have largely focused on single risk factors and not explored how a wide range of risk factors including behavioural factors influence wellbeing. Additionally, given that many such behaviours are strongly socially patterned, studies have thus far paid little attention to how potential risk factors are confounding by socioeconomic position and issues relating to the clustering of behaviours and wellbeing within localities.

Policy initiatives to improve wellbeing and mental health amongst young people have largely focused upon cognitive and psychological factors related to resilience to adversity, and have paid little attention to the contribution of non-psychological modifiable factors, such as other lifestyle behaviours. Understanding the potential contribution of modifiable behavioural factors to adolescent wellbeing may inform different strategies to improve young people's wellbeing [27].

Confidential: For Review Only

We used a very large recent nationally-representative and population-based survey of English 15 year-olds to examine the contribution of individual-level modifiable behaviours to wellbeing, including potentially protective (sleep, reading and physical activity) and risk behaviours (substance use, unhealthy eating habits and excessive screen time). Our objective was to identify modifiable behavioural factors for mental wellbeing in boys and girls using an adolescent-specific measure and accounting for deprivation, ethnicity and clustering within local authorities (LAs).

Methods

Study design and sample

The What About Youth study (WAY) is a large-scale youth-oriented survey funded by Department of Health (DH) in England and carried out by NHS Digital in 2014 [28]. The primary aim of the survey was to collect robust local authority level data on youth health behaviours and general health to improve their health outcomes. Study participants were born between 01/09/1998 and 31/08/1999 and turned 15 years old in the academic year 2013/14. A random sampling methodology was employed to draw 298,080 participants from the National Pupil Database (NPD). The sample size was calculated to attain 1000 young people in each of 152 LAs in England with 2 LAs were merged with their nearest neighbours due to small size.

The analysis sample was 120,115 individuals, of whom 16% responded online and 84% via postal means (2,835 opted out). The response rates differed by gender, with adjusted response rates of 35% in boys and 49% in girls, and by deprivation, ethnicity and LA. Non-response weights using these factors were calculated to provide alignment between the achieved and target samples and these weights were used in our analyses. We obtained a fully anonymised cohort data electronically from the United Kingdom (UK) Data Service website[28]

Confidential: For Review Only

Measures

Mental wellbeing was measured using the Warwick-Edinburgh Mental Wellbeing Scale (WEMWBS), a 14 item population-level measure [29]. It is validated to use in adolescents' age 13 or more and focus primarily on positive aspects of mental wellbeing. Participants indicate how often they feel like each of the 14 items using a 5-point scale that ranges from 5 "all the time" to 1 "none of the time" [30]. Total scores ranged from 14-70 and were calculated by summing each participant's responses. The potential explanatory behavioural variables were identified from the literature review of previous publication. A detail description of each variable is given in appendix A of supplementary material.

A composite variable for risk behaviour index for substance use was constructed by the summation of three dichotomous risk behaviour variables: (a) Smoking: If currently smokes (b) Drinking alcohol: If drinks once a month or more frequently (c) Cannabis use: If ever tried cannabis. Based on the combination of risk behaviours, the composite score was categorised as 0= none, 1= only one, 2 =any two and 3 =all three. Similarly, composite unhealthy eating habit index was derived from (a) Skipping Breakfast: If avoided breakfast in last 7 days (b) Poor diet: If consumed less than 5 portions of fruits and vegetables a day (c) Takeaway food: If consumed takeaway food in past 7 days. Based on a combination of unhealthy behaviours, the composite score was categorised into 0= none, 1= only one, 2 =any two and 3 =all three. Physical active for 60+ minutes for at least 5 days were classified as "physically active" and the rest "physically inactive." This threshold was defined in line with government recommendations [31 32], except for the intensity of exercise which was not available in the dataset. The selected threshold of activity level was taken at 5 days a week, as only 13% reported being physically active for 7 days a week. A screen time variable was computed based on reported weekend and weekday usage of television, internet, smartphone and computer games. Subjects were categorised into "≥7hrs/day", "About 5-6 hours/day",

Confidential: For Review Only

"About 3 -4 hours/day", "About 2 hrs/day" and "≤1hrs/day". Time spent reading on weekends and weekdays and the response options ranged from none to seven hours per day. We recoded the variable as "none", about half an hour/day", "about 1 hour/day" and "2 hours /day". The frequency of eight hours sleep in the last seven days was coded as "every day", "most days", "and some days" and "not in the past 7 days". Bullying was measured with Olweus Bully/Victim Questionnaire, a reliable 8-item scale used to assess the bullying victimisation [33]. We combined responses to create one overall measure of bullying experience (yes/no). In line with previous studies[34], adolescents who were bullied more than "two or three times a month" were categorised as bullying victims.

Ethnicity, area deprivation, and mode of questionnaire completion were selected as confounders in the relationship between wellbeing and potential explanatory variables as shown in previous studies [35]. Ethnicity was self-identified by participants' and was an adaptation of the 2001 UK census categories, supplemented by questions on the national group. English Index of Multiple Deprivation (IMD) was used as a measure of relative deprivation for small areas [36]. The IMD scores were divided into three deprivation categories defined by quintiles of the national distribution: 1 and 2 (high deprivation), 3 (average), 4 and 5 (low deprivation). The participants were allowed to choose either online or postal modes of questionnaire completion.

Statistical Analyses

We conducted unadjusted and adjusted multilevel regression, in Stata 14 (Stata Corp, College Station, TX). For wellbeing scores, the interaction between gender and health behaviours was statistically significant ($p<0.001$) and therefore, analyses were stratified by gender. All variables were plotted to check the distribution and normality was checked with

Confidential: For Review Only

Kolmogorov-Smirnov (K-S) test. All estimates were weighted by representativeness of participants to compensate for the disproportionate selection of sample and non-response bias. Spearman $\chi 2$ tests were used to compare the distribution of explanatory variables by gender. An unadjusted analysis was run to test the association between each independent variable (substance use, unhealthy eating habits, screen time, reading, bullying, physical activity and sleeping hours) and outcome adolescent wellbeing (Model 1). The analyses were repeated in a multivariable analysis where ethnicity, mode of questionnaire delivery and IMD were added as confounders between each risk factor and outcome (Model 2). In model 3, all explanatory factors and covariates were included simultaneously to obtain associations between each variable and wellbeing scores after adjusting for confounder, and other explanatory variables were presented. LAs were treated as random effects in all models. Models were also tested for significant quadratic terms signifying curvilinear relationships; this was only significant for reading. The intraclass correlations coefficient (ICC), being the proportion of total variance attributable to differences at the LA level, was estimated using multilevel models for wellbeing with adjustment for IMD and ethnicity and mode of completion.

Results

In total, 47.8% boys and 52.2% of girls participated in the study (Table A1). Of these, boys had higher average wellbeing score in comparison to girls. The intracluster correlation coefficient (ICC) for the wellbeing score was 0.003 in both boys and girls suggesting that variance in adolescent wellbeing is small at local authority level.

Table A1 shows that there were significant differences in the distribution of explanatory variables between boys and girls. Girls had higher risk factors such as substance use, unhealthy eating habits, screen time and reported more bullying than boys. Protective

Confidential: For Review Only

variables such as sleeping hours (more than8 hours) and reading were also significantly higher in girls than in boys.

All explanatory variable and IMD significantly associated with wellbeing in both sexes (Table A2). Wellbeing in both sexes decreased with use of substance use, unhealthy eating habits, bullying and physical activity and longer screen time in both sexes. Protective factors, such as, sleeping more than eight hours and reading more than two hours associated with higher wellbeing in both sexes.

In the adjusted multivariable models adjusted for covariates (Table A3), poorer wellbeing was associated with multiple substances use and multiple unhealthy eating habits in a dose-dependent fashion. Being physically inactive, longer screen time and experiencing bullying were both associated with decrements in wellbeing in both sexes, with the association being stronger in girls than in boys.

Higher wellbeing was associated with number of days young people achieved more than eight hours of sleep again in a dose-dependent fashion. Habitual reading most days was associated with higher wellbeing although there was no evidence of a dose-response above 30 minutes per day in the fully adjusted model (Figure 1). Young people from black ethnic groups had significantly higher wellbeing in both sexes. Deprivation was not associated with wellbeing amongst boys but was amongst girls.

Discussion

This study broadens our understanding of risk and protective factors associated with wellbeing in adolescence, using a very large nationally representative survey to examine a wide spectrum of behavioural and psychosocial factors relating to youth wellbeing and taking into account deprivation and clustering at local authority level. The study shows that young people who reported lower levels of wellbeing were more likely to have engaged in multiple

Confidential: For Review Only

unhealthy eating habits and substance use; be victims of bullying; have exercised insufficiently; have exceeded recommended screen time use. These findings were robust to mutual adjustment for all variables and for deprivation, ethnicity and mode of questionnaire delivery. A dose-response pattern was also observed between wellbeing and health behaviours such as substance use, unhealthy eating habits and sleeping pattern. A decrease in the number of risk behaviours and increase in the number of days slept for more than eight hours, corresponded with an increase in average levels of wellbeing. The impact of deprivation on wellbeing was surprisingly small, as was local authority locality variance, suggesting that variance in wellbeing lies largely in individual behavioural and school factors which was unavailable in this dataset.

Since different studies have used different variables to define various aspects of wellbeing in the analyses, comparison with other studies is difficult. We found boys reported higher mean wellbeing than girls, consistent with national reports for England [37 38], but in contrast to findings from recent Health Survey England (HSE) 2015, where only slight gender variations were observed [39]. The proportions reporting each of the behaviours were broadly similar to those found in other recent national surveys [27 40]. Girls reported higher levels of risky health behaviours including current smoking, alcohol consumption, bullying, on the other hand, boys were more likely to report higher levels of physical activity and these trends were consistent with findings from the Health Behaviour in School-aged Children (HBSC) England study [41].

These findings corroborate with two previous studies where happiness as a marker of wellbeing was found to be positively associated with multiple health protective behaviours (sport's participation and healthier eating) and negatively associated with multiple risk behaviours (smoking, alcohol use and heavy screen use) in adolescents[42 43]. Our finding that substance use was associated with lower wellbeing is similar to that seen in other studies, as were our findings for being bullied [35]. The association of sleep duration and reading with

Confidential: For Review Only

wellbeing in young people has been little studied. Leisure time and adequate sleep have been identified as being associated with wellbeing[19 22], however, ours is the first to examine these alongside other behavioural and psychological factors.

We found an association between deprivation and lower wellbeing, although in contrast to previous studies[27] the effect size was small and we found no association in boys. This may reflect the lack of adjustment for multiple behaviours, ethnicity and area effects in other studies. Our finding suggests that much of what has previously been understood as deprivation effects may be mediated by unhealthy behaviours themselves associated with deprivation. We found that young people black ethnic groups reported significantly higher wellbeing in both sexes, consistent with previous UK findings [25]. However, the reasons for this remain unclear and require further study. We found this association to be robust to adjustment for deprivation and all significant behavioural and psychological factors, suggesting this likely relates to factors not measured in our study.

We used a large, nationally representative sample of ethnically and socioeconomically diverse adolescents. Prior studies have examined very few health behaviours and relied on proxy measures of wellbeing rather than on population-level wellbeing measures that tap into both feelings and psychological flourishing. In our study, the associations between wellbeing and behavioural factors were examined within a multivariable and multilevel framework, using a validated wellbeing scale with robust psychometric properties.

Our findings are subject to a number of limitations. Our data were cross-sectional, and thus the direction of causality is unclear for the behavioural variables studied. Participant responses could be influenced by social desirability, and those with poor wellbeing may be inclined towards endorsing questions more than others, thus introducing bias. The direction

Confidential: For Review Only

of such biases is unclear, however, we note that girls (who had a higher response rate in the overall survey) reported higher levels of both more risky and protective behaviours than boys, potentially reflecting social desirability biases. All variables used were by self-reported except for area-level deprivation. We have also repeated analysis excluding the outliers, and that did not materially affect the findings. We used bullying victimization as a proxy for psychological problems due to the lack of more appropriate variables in the dataset; thus it is possible that some of the associations seen here result from inadequate adjustment for psychological issues. We combined variables across domains into composite variables; this may have introduced bias although the direction of bias is unclear.

Conclusions

Our findings suggest that promoting healthy sleep, reading, and healthy eating behaviours may present important policy targets for enhancing adolescent well-being in addition to more accepted foci on physical activity, screen time and bullying. Future work is needed to examine these factors within a longitudinal causal framework**.**

Confidential: For Review Only

What is already known on this topic?

Young people's wellbeing may be affected by multiple individual and contextual factors.

Key determinants of adolescent wellbeing remain unclear

Few studies have examined a wide range of potential determinants whilst adjusting for area-level deprivation.

What this study adds?

Findings support current policy foci on bullying, physical activity, and screen-time as correlates of wellbeing amongst young people

Sleep and eating behaviours may also be important policy targets for promoting adolescent wellbeing.

A coherent policy framework to promote adolescent wellbeing needs to be multifaceted and consider a range of health factors in young people's lives

Confidential: For Review Only

References

1. James S, Hale L. Sleep Duration and Child Wellbeing: A Nonlinear Association. *Journal of clinical child and adolescent psychology : the official journal for the Society of Clinical Child and Adolescent Psychology, American Psychological Association, Division 53* 2017;46(2):258-68. doi: 10.1080/15374416.2016.1204920
2. RCPCH. State of Child Health Report Royal College of Paediatrics and Child Health; 2017 [Available from: http://www.thehealthwell.info/node/1062390 accessed 6th November 2017.
3. Patton GC, Sawyer SM, Santelli JS, et al. Our future: a Lancet commission on adolescent health and wellbeing. *Lancet (London, England)* 2016;387(10036):2423-78. doi: 10.1016/s0140-6736(16)00579-1 [published Online First: 2016/05/14]
4. Oxford University Press. Oxford Dictionary. Oxford: Oxford University Press 2016.
5. Žukauskienė R. Adolescence and Well-Being. In: Ben-Arieh A, Casas F, Frønes I, et al., eds. Handbook of Child Well-Being: Theories, Methods and Policies in Global Perspective. Dordrecht: Springer Netherlands 2014:1713-38.
6. Keyes CL. Promoting and protecting mental health as flourishing: a complementary strategy for improving national mental health. *The American psychologist* 2007;62(2):95-108. doi: 10.1037/0003-066x.62.2.95 [published Online First: 2007/02/28]
7. Das JK, Salam RA, Lassi ZS, et al. Interventions for Adolescent Mental Health: An Overview of Systematic Reviews. *Journal of Adolescent Health* 2016;59(4, Supplement):S49-S60. doi: https://doi.org/10.1016/j.jadohealth.2016.06.020
8. Landstedt E, Persson S. Bullying, cyberbullying, and mental health in young people. *Scandinavian journal of public health* 2014;42(4):393-9. doi: 10.1177/1403494814525004 [published Online First: 2014/03/13]

Confidential: For Review Only

9. Dinisman T, Andresen S, Montserrat C, et al. Family structure and family relationship from the child well-being perspective: Findings from comparative analysis. *Children and Youth Services Review* 2017;80(Supplement C):105-15. doi: https://doi.org/10.1016/j.childyouth.2017.06.064

10. Raja SN, McGee R, Stanton WR. Perceived attachments to parents and peers and psychological well-being in adolescence. *Journal of Youth and Adolescence* 1992;21(4):471-85. doi: 10.1007/bf01537898

11. García Bacete FJ, Marande Perrin G, Schneider BH, et al. Effects of School on the Well-Being of Children and Adolescents. In: Ben-Arieh A, Casas F, Frønes I, et al., eds. Handbook of Child Well-Being: Theories, Methods and Policies in Global Perspective. Dordrecht: Springer Netherlands 2014:1251-305.

12. Farmer S, Hanratty B. The relationship between subjective wellbeing, low income and substance use among schoolchildren in the north west of England: a cross-sectional study. *Journal of Public Health* 2012;34(4):512-22. doi: 10.1093/pubmed/fds022

13. Phillips-Howard PA, Bellis MA, Briant LB, et al. Wellbeing, alcohol use and sexual activity in young teenagers: findings from a cross-sectional survey in school children in North West England. *Substance abuse treatment, prevention, and policy* 2010;5:27. doi: 10.1186/1747-597x-5-27 [published Online First: 2010/11/12]

14. Taylor G, McNeill A, Girling A, et al. Change in mental health after smoking cessation: systematic review and meta-analysis. *BMJ : British Medical Journal* 2014;348 doi: 10.1136/bmj.g1151

15. Conner TS, Brookie KL, Carr AC, et al. Let them eat fruit! The effect of fruit and vegetable consumption on psychological well-being in young adults: A randomized controlled trial. *PLoS ONE* 2017;12(2):e0171206. doi: 10.1371/journal.pone.0171206

16. Oddy WH, Robinson M, Ambrosini GL, et al. The association between dietary patterns and mental health in early adolescence. *Preventive Medicine* 2009;49(1):39-44. doi: https://doi.org/10.1016/j.ypmed.2009.05.009

Confidential: For Review Only

17. Lien L. Is breakfast consumption related to mental distress and academic performance in adolescents? *Public Health Nutrition* 2007;10(4):422-28. doi: 10.1017/S1368980007258550 [published Online First: 04/01]

18. Ussher MH, Owen CG, Cook DG, et al. The relationship between physical activity, sedentary behaviour and psychological wellbeing among adolescents. *Social Psychiatry and Psychiatric Epidemiology* 2007;42(10):851-56. doi: 10.1007/s00127-007-0232-x

19. Fuligni AJ, Hardway C. Daily Variation in Adolescents' Sleep, Activities, and Psychological Well-Being. *Journal of Research on Adolescence* 2006;16(3):353-78. doi: 10.1111/j.1532-7795.2006.00498.x

20. Przybylski AK, Weinstein N. A Large-Scale Test of the Goldilocks Hypothesis:Quantifying the Relations Between Digital-Screen Use and the Mental Well-Being of Adolescents. *Psychological Science* 2017;28(2):204-15. doi: 10.1177/0956797616678438

21. Hamer M, Yates T, Sherar LB, et al. Association of after school sedentary behaviour in adolescence with mental wellbeing in adulthood. *Preventive Medicine* 2016;87(Supplement C):6-10. doi: https://doi.org/10.1016/j.ypmed.2016.02.021

22. Trainor S, Delfabbro P, Anderson S, et al. Leisure activities and adolescent psychological well-being. *Journal of Adolescence* 2010;33(1):173-86. doi: https://doi.org/10.1016/j.adolescence.2009.03.013

23. Rose T, Joe S, Williams A, et al. Measuring Mental Wellbeing Among Adolescents: A Systematic Review of Instruments. *Journal of Child and Family Studies* 2017;26(9):2349-62. doi: 10.1007/s10826-017-0754-0

24. Pollard EL, Lee PD. Child Well-being: A Systematic Review of the Literature. *Social Indicators Research* 2003;61(1):59-78. doi: 10.1023/a:1021284215801

25. Patalay P, Fitzsimons E. Correlates of Mental Illness and Wellbeing in Children: Are They the Same? Results From the UK Millennium Cohort Study. *Journal of the American Academy of Child & Adolescent Psychiatry* 2016;55(9):771-83. doi: 10.1016/j.jaac.2016.05.019

26. Proctor CL, Linley PA, Maltby J. Youth Life Satisfaction: A Review of the Literature. *Journal of Happiness Studies* 2009;10(5):583-630. doi: 10.1007/s10902-008-9110-9

Confidential: For Review Only

27. Candace Currie, Cara Zanotti, Antony Morgan, et al. Social determinants of health and well-being among young people. International report from the 2009/2010 survey. Health Behavior in school-age children (HBSC) study. Denmark: WHO Regional Office for Europe, 2012:252.

28. HSCIC. What About YOUth? Survey. In: Service UD, ed. Data Collection. 2nd ed. England: HEALTH AND SOCIAL CARE INFORMATION CENTRE, 2014.

29. Tennant R, Hiller L, Fishwick R, et al. The Warwick-Edinburgh Mental Well-being Scale (WEMWBS): development and UK validation. *Health and Quality of Life Outcomes* 2007;5:63-63. doi: 10.1186/1477-7525-5-63

30. Taggart F, Stewart-Brown S, Parkinson J. Warwick-Edinburgh Mental Well-being Scale (WEMWBS) Scotland: NHS Health Scotland; 2015 [Available from: http://www.healthscotland.com/uploads/documents/26787-WEMWBS%20User%20Guide%20Version%202%20May%202015.pdf accessed 19th October 2017.

31. Morgan K, Hallingberg B, Littlecott H, et al. Predictors of physical activity and sedentary behaviours among 11-16 year olds: Multilevel analysis of the 2013 Health Behaviour in School-aged Children (HBSC) study in Wales. *BMC Public Health* 2016;16:569. doi: 10.1186/s12889-016-3213-8

32. Booth ML, Okely AD, Chey T, et al. The reliability and validity of the physical activity questions in the WHO health behaviour in schoolchildren (HBSC) survey: a population study. *British journal of sports medicine* 2001;35(4):263-7. [published Online First: 2001/07/31]

33. Olweus D. The Revised Olweus Bully/Victim Questionnaire. University of Bergen: Research Center for Health Promotion (HEMIL Center) 1996.

34. Cook CR, Williams KR, Guerra NG, et al. Predictors of bullying and victimization in childhood and adolescence: A meta-analytic investigation. *School Psychology Quarterly* 2010;25(2):65-83. doi: 10.1037/a0020149

35. Przybylski AK, Bowes L. Cyberbullying and adolescent well-being in England: a population-based cross-sectional study. *The Lancet Child & Adolescent Health* 2017;1(1):19-26. doi: 10.1016/S2352-4642(17)30011-1

Confidential: For Review Only

36. Department for Communities and Local Government. The English indices of deprivation London2015 [Available from: https://www.gov.uk/government/uploads/system/uploads/attachment_data/file/465791/English_Indices_of_Deprivation_2015_-_Statistical_Release.pdf

37. Office of National Statistics. Measuring National Well-being: Insights into children's mental health and well-being. England, 2015.

38. Office of National Statistics. Young people's well-being England: Office for National Statistics; 2017 [Available from: https://www.ons.gov.uk/peoplepopulationandcommunity/wellbeing/articles/youngpeopleswellbeingandpersonalfinance/2017 accessed 10th November 2017.

39. Fuller E, Morris S. Health Survey for England. London NHS Digital, 2015.

40. National Centre for Social Research. Smoking, Drinking and Drug Use among Young People. In: National Foundation for Educational Research, ed.: UK Data Service, 2012.

41. Brooks F, Magnusson J, Klemera E, et al. Health Behaviours of School-Aged Children (HBSC) 2013-2014 England2014 [Available from: www.hbscengland.com.

42. Booker CL, Skew AJ, Sacker A, et al. Well-Being in Adolescence—An Association With Health-Related Behaviors:Findings From Understanding Society, the UK Household Longitudinal Study. *The Journal of Early Adolescence* 2014;34(4):518-38. doi: 10.1177/0272431613501082

43. Booker CL, Skew AJ, Kelly YJ, et al. Media Use, Sports Participation, and Well-Being in Adolescence: Cross-Sectional Findings From the UK Household Longitudinal Study. *American Journal of Public Health* 2015;105(1):173-79. doi: 10.2105/AJPH.2013.301783

Confidential: For Review Only

Table A1. Descriptive statistics for well-being scores and explanatory variables under study, by gender

	Total	Boys		Girls		
	N	N	Mean (SD) / %	N	Mean (SD) / %	P value
	**120,115**	**57,153**	**47.58%**	**62,962**	**52.42%**	
**WEMWBS Scores** [1]	117,842	56,352	47.82%	61,490	52.18%	<0.0001
Mean (SD)			50 (8.60)		45 (9.66)	
**Substance use** [2]	71,133	32,516	45.71%	38617	54.29%	<0.0001
None		17,133	52.69	19932	51.61	
One		10,783	33.16	12150	31.46	
Two		3,010	9.26	3,785	9.8	
Three		1,590	4.89	2,750	7.12	
**Unhealthy Eating Habits** [3]	115918	55,289	47.70%	60,629	52.30%	<0.0001
None		12,081	21.85	11,951	19.71	
One		20,287	36.69	20,389	33.63	
Two		16,373	29.61	19,064	31.44	
Three		6,548	11.84	9,225	15.22	
**Sleeping hours (>8 hrs)**	117,516	56, 207	47.83%	61307	52.17%	<0.0001
Not in the past 7 days		3676	6.54	6922	11.29	
some days		11051	19.66	16051	26.18	
most days		20, 121	35.8	20928	34.14	
everyday		21, 361	38	17406	28.39	
**Bullying**	117,744	56,309	47.82%	61,435	52.18%	<0.0001
No		45,959	81.62	45,094	73.40	
yes		10,350	18.38	16,341	26.60	
**Physical activity**	118450	56,674	47.85	61776	52.15	<0.0001
Physically active		48,172	85	44,348	71.79	
Inactive		8,502	15	17,428	28.21	
**Screen time**	118,845	56,892	47.87	61,943	52.13%	<0.0001
About 2 hrs/day		3,609	6.34	3,469	5.6	
≤1hrs/day		912	1.6	929	1.5	
About 3 -4 hours/day		18,621	32.73	17,311	27.95	
About 5-6 hours/day		17,198	30.23	17,850	28.82	
≥7hrs/day		16,559	29.1	22387	36.14	
**Reading**	118,140	56,513	47.84	61,627	52.16	<0.0001
None		14,278	25.26	9,875	16.02	
About Half an hour/day		17,572	31.09	15,363	24.93	
About 1 hr/day		12,505	22.13	13,442	21.81	
≥2hrs/day		12,158	21.51	22,947	37.24	

[1] WEMWBS : Warwick-Edinburgh Mental Wellbeing score
[2] Risk behaviours include smoking, drinking and cannabis use
[3] Unhealthy eating habits include skipping breakfast, not having 5 portions of fruits and vegetables and consumption of takeaway food.

Confidential: For Review Only

Confidential: For Review Only

Table A2: Univariate analysis between well-being and explanatory variables, by gender

	MODEL[1]			
	Boys		Girls	
	B	95 % CI	*B*	95 % CI
**Substance Use**				
None	Reference		Reference	
One	-0.34*	[-0.56,-0.13]	-1.48**	[-1.70,-1.27]
Two	-2.19**	[-2.60,-1.78]	-4.96**	[-5.35,-4.57]
Three	-3.56**	[-4.16,-2.95]	-5.89**	[-6.47,-5.30]
**Unhealthy Eating Habits**				
None	Reference		Reference	
One	-1.65**	[-1.88,-1.43]	-2.63**	[-2.91,-2.34]
Two	-3.36**	[-3.60,-3.12]	-4.85**	[-5.13,-4.58]
Three	-5.00**	[-5.39,-4.61]	-6.49**	[-6.83,-6.14]
**Sleeping hours (>8 hrs)**				
Not in the past 7 days	Reference		Reference	
some days	2.99**	[2.51,3.47]	4.71**	[4.39,5.04]
most days	5.59**	[5.15,6.03]	8.39**	[8.06,8.72]
everyday	7.49**	[7.07,7.87]	10.70**	[10.35,11.06]
**Bullying**				
No	Reference		Reference	
Yes	-4.70**	[-3.59,-3.31]	-5.84**	[-5.25,-4.90]
**Physical activity**				
Physically active	Reference		Reference	
Inactive	-3.85**	[-4.11,-3.61]	-2.77**	[-3.04,-2.51]
**Screen time**				
About 2 hrs/day	Reference		Reference	
≤1hrs/day	0.07	[-0.59, 0.73]	0.08	[-0.69, 0.85]
About 3 -4 hours/day	-1.22**	[-1.60,-0.84]	-1.27**	[-1.71,-0.83]
About 5-6 hours/day	-2.17**	[-2.51,-1.83]	-3.06**	[-3.52,-2.60]
>= 7 hours / day	-3.72**	[-4.12,-3.32]	-5.38**	[-5.80,-4.97]
**Reading**				
None	Reference		Reference	
About Half an hour/day	1.61**	[1.41,1.82]	2.17**	[1.88,2.46]
About 1 hr/day	2.25**	[2.06,2.44]	2.54**	[2.26,2.82]
≥2hrs/day	2.27***	[2.01,2.52]	2.35**	[2.05,2.66]
**IMD Scores**				
High deprivation	Reference		Reference	

Confidential: For Review Only

Average deprivation	0.57**	[0.31,0.81]	0.35**	[0.10,0.59]
Least deprivation	1.05**	[0.87,1.21]	1.16**	[0.92,1.40]
**Ethnicity**				
White	Reference		Reference	
Mixed	0.18	[-0.29,0.65]	-0.44*	[-0.82,-0.06]
Asian	-0.54*	[-0.85,-0.23]	0.98**	[0.59,1.38]
Black	0.91**	[0.42,1.39]	1.10**	[0.67,1.52]
Other	-0.45*	[-0.84,-0.04]	0.30	[-0.09,0.70]

[1]Unadjusted model taking into account clustering at Local Authority Level, Multilevel models fitted with weighted design weights, Quadratic function added to reading.
** p<0.001, * p<0.05

Confidential: For Review Only

Table A3: Gender - stratified partially-adjusted and fully-adjusted multilevel modelling for well-being and explanatory variables

	Boys				Girls			
	MODEL[2]		MODEL[3]		MODEL[2]		MODEL[3]	
**Variables**	B	95 % CI	B	95 % CI	B	95 % CI	B	95 % CI
**Substance use**								
None	Reference		Reference		Reference		Reference	
One	-0.40**	[-0.61,-0.18]	-0.14	[-0.36,0.08]	-1.51**	[-1.73,-1.30]	-0.77**	[-0.97,-0.57]
Two	-2.16**	[-2.57,-1.75]	-1.05**	[-1.42,-0.67]	-4.84**	[-5.22,-4.46]	-2.67**	[-3.01,-2.33]
Three	-3.50**	[-4.11,-2.88]	-1.63**	[-2.16,-1.09]	-5.80**	[-6.36,-5.23]	-2.79**	[-3.35,-2.24]
**Eating Habits**								
None	Reference		Reference		Reference		Reference	
One	-1.63**	[-1.85,-1.41]	-0.89**	[-1.18,-0.60]	-2.63**	[-2.92,-2.34]	-1.37**	[-1.69,-1.06]
Two	-3.31**	[-3.55,-3.08]	-1.84**	[-2.14,-1.54]	-4.84**	[-5.12,-4.55]	-2.29**	[-2.64,-1.96]
Three	-4.95**	[-5.34,-4.56]	-2.44**	[-2.82,-2.06]	-6.46**	[-6.79,-6.10]	-2.61**	[-3.04,-2.18]
**Sleeping hours (>8 hrs)**								
Not in the past 7 days	Reference		Reference		Reference		Reference	
some days	2.98**	[2.50,3.45]	2.69**	[2.10,3.28]	4.69**	[4.36,5.01]	3.71**	[3.34,4.08]
most days	5.55**	[5.11,5.99]	4.30**	[3.78,4.82]	8.35**	[8.01,8.69]	6.64**	[6.28,7.00]
everyday	7.45**	[7.03,7.86]	5.79**	[5.29,6.28]	10.65**	[10.29,11.01]	8.16**	[7.72,8.60]
**Bullying**								
No	Reference		Reference		Reference		Reference	
Yes	-4.64**	[-4.86,-4.42]	-3.78**	[-4.09,-3.48]	-5.77**	[-5.98,-5.56]	-4.01**	[-4.23,-3.78]
**Physical activity**								
Physically active	Reference		Reference		Reference		Reference	
Inactive	-3.78**	[-4.02,-3.55]	-2.63**	[-2.95,-2.30]	-2.77**	[-3.04, -2.50]	-1.70**	[-2.01,-1.39]
**Screen time**								
About 2 hrs/day	Reference		Reference		Reference		Reference	
≤1hrs/day	0.09	[-0.57,0.75]	0.34	[-0.58,1.26]	0.09	[-0.69,0.87]	-0.38	[-1.49,0.72]
About 3 -4 hours/day	-1.22**	[-1.60,-0.83]	-0.61*	[-0.99,-0.23]	-1.26**	[-1.70,-0.83]	-0.54	[-1.14,0.05]

Confidential: For Review Only

About 5-6 hours/day	-2.15**	[-2.50,-1.81]	-0.82**	[-1.27,-0.37]	-3.04**	[-3.49,-2.58]	-1.21**	[-1.75,-0.67]
>= 7 hours / day	-3.67**	[-4.06,-3.27]	-1.20**	[-1.65,-0.75]	-5.32**	[-5.73,-4.90]	-1.81**	[-2.29,-1.33]
**Reading**								
None	Reference		Reference		Reference		Reference	
About Half an hour/day	1.59**	[1.38,1.80]	0.57**	[0.26,0.88]	2.13**	[1.84,2.42]	0.56**	[0.23,0.90]
About 1 hr/day	2.24**	[2.04,2.43]	1.04**	[0.79,1.29]	2.51**	[2.24,2.78]	0.60**	[0.26,0.93]
≥2hrs/day	2.28**	[2.05,2.54]	0.90**	[0.55,1.25]	2.34**	[2.03,2.64]	0.33*	[0.02,0.64]
**IMD Scores**								
High deprivation	--	--	Reference		--	--	Reference	
Average deprivation	--	--	-0.12	[-0.38,0.14]	--	--	0.16	[-0.16,0.49]
Least deprivation	--	--	0.17	[-0.06,0.40]	--	--	0.36*	[0.06,0.66]
**Ethnicity**								
White	--	--	Reference		--	--	Reference	
Mixed	--	--	-0.06	[0.81,-0.59]	--	--	0.44	[-0.08,0.96]
Asian	--	--	-0.09	[0.81,-0.84]	--	--	0.35	[-0.53,1.23]
Black	--	--	0.99*	[0.01,0.25]	--	--	1.75**	[1.20,2.31]
Other	--	--	-0.59*	[0.02,-1.09]	--	--	-0.19	[-0.88,0.49]
**Mode of questionnaire delivery**								
Online	--	--			--	--		
paper	--	--	0.24	[-0.03,0.51]	--	--	1.26**	[0.99,1.53]

[2] Each predictor variable adjusted for ethnicity, mode of questionnaire delivery and Index of multiple deprivation
[3] All Variables mutually adjusted for each other
Note: Multilevel models fitted with weighted design weights, Quadratic function added to reading
** p<0.001, * p<0.05

Confidential: For Review Only

Figure 1

Relationship between wellbeing and health behaviours: (A) Risk Factors (B) Protective Factors

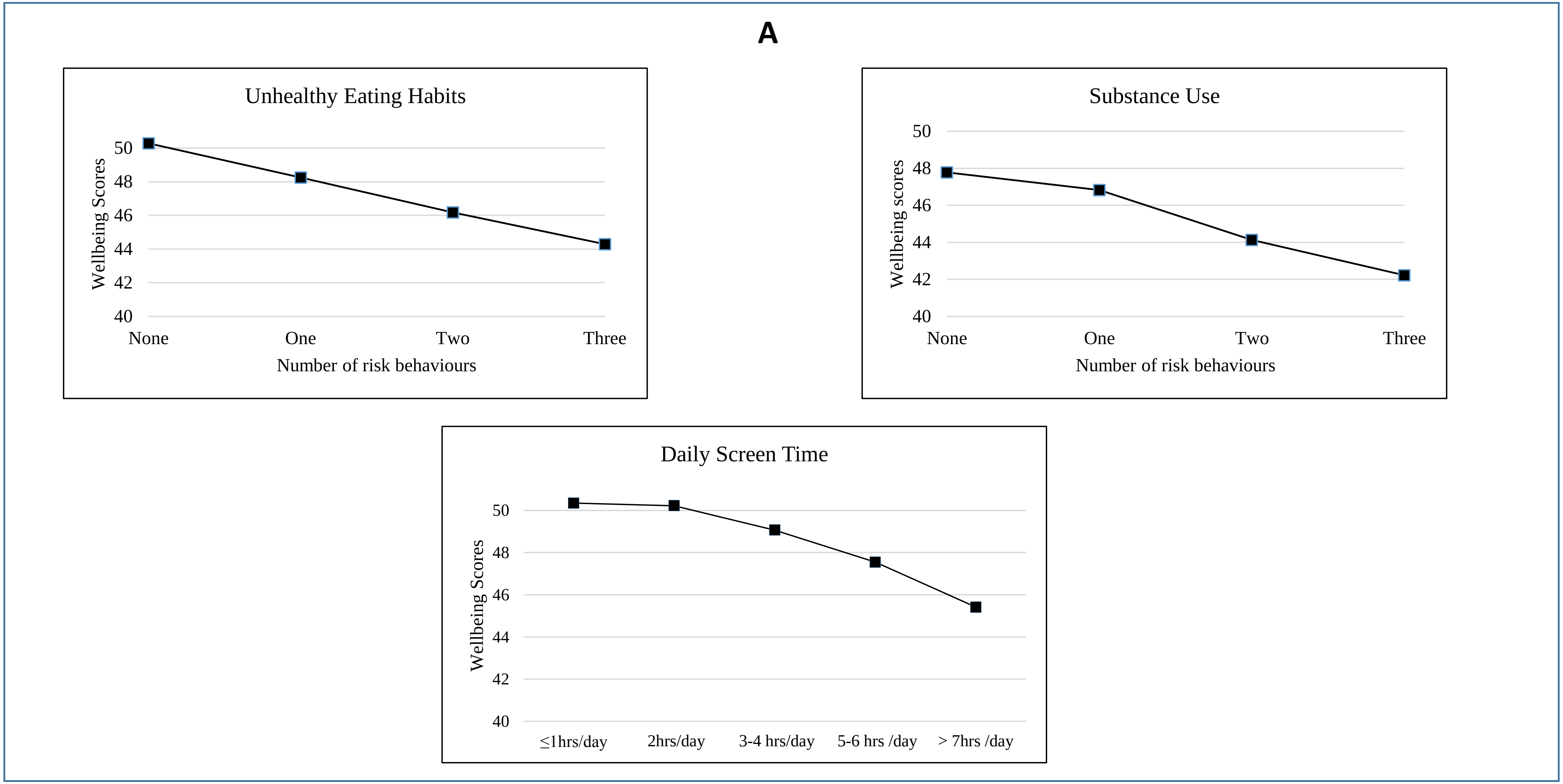

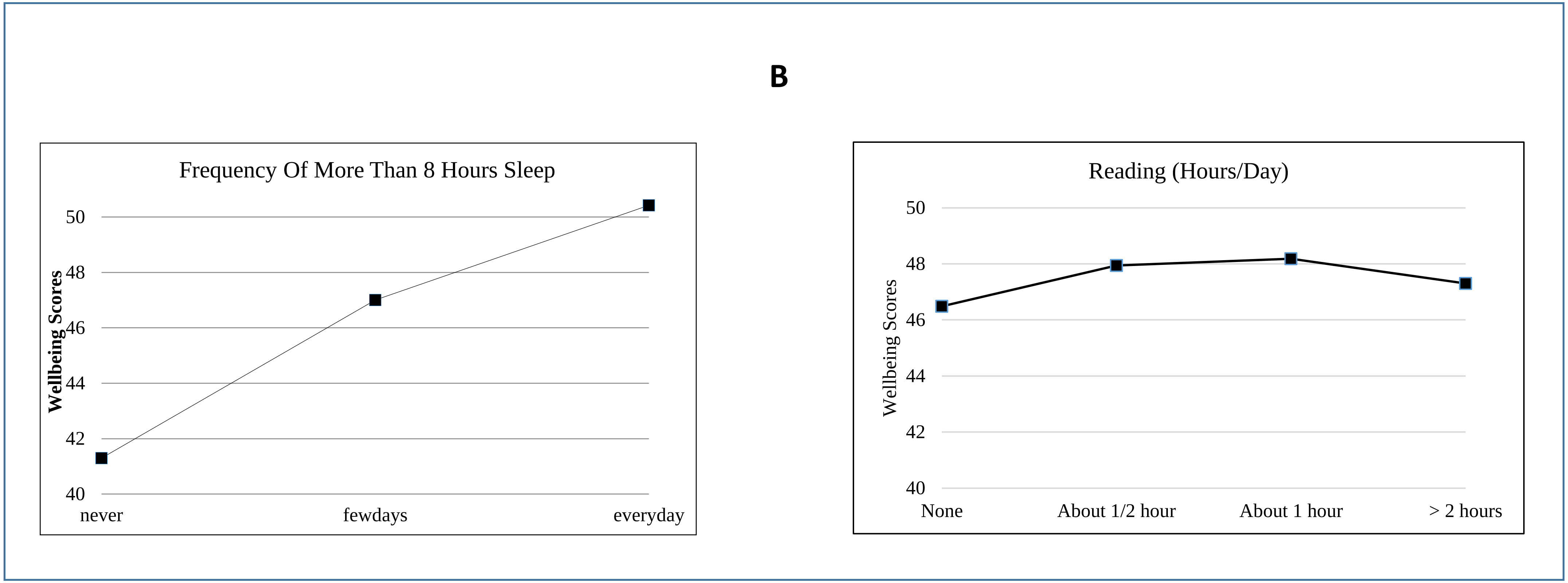
B
Frequency Of More Than 8 Hours Sleep
Wellbeing Scores
50
48
46
44
42
40
never
fewdays
everyday
Reading (Hours/Day)
Wellbeing Scores
50
48
46
44
42
40
None
About 1/2 hour
About 1 hour
> 2 hours

APPENDIX A

VARIABLE DESCRIPTION

Composite variables	Original variables	Variable labels	Composite variables
**Substance use risk [1-6]**	**Definitions were:**		**Alcohol + smoking + cannabis**
	Smoking: Cigarette smoking status (currently smoke)	SmkSt2	(1) Smoking risk: If currently smokes (2) Drinking risk: If drinks once a month or more frequently (3) Cannabis risk: If ever tried cannabis. Each item was coded 0 for “no” and 1 for “yes”. The composite score obtained ranged from zero to three based on the number of risk behaviours; (0= None, 1= Only one, 2 =Any two and 3 =All three
	Drinking: Alcohol drinking status (Usually have an alcoholic drink once a month or more frequently)	alcfrq1	
	Cannabis: Cannabis use status (Ever tried cannabis)	Cantry	
**Physical Activity [7]**	**Physical activity: in line with general recommendations**		**Duration of exercise + Frequency of exercise + Number of days met recommended guidelines**
	How many days physically active for 60+ minutes, in last 7 days (Active for more than 5 days)	Physact	The first question on moderate to vigorous physical activity was “Over the past 7 days, on how many days you were physically active for a total of at least 60 minutes per day and responses ranged from 0 to 7. Second, the participants were asked “Outside school hours: how often do you usually exercise in your free time so much that you get out of breath or sweat?” with responses from less than a once a month to every day. The question on duration was “Outside school hours: how many hours do you usually exercise in your free time so much that you get out of breath or sweat?”, with responses from none to 7 or more hours. Adolescents who exercised for more than twice a week for an hour or more or were physically active for 60+ minutes for at least 5 days were classified as “physically active” and the rest “physically inactive”.
	How often usually exercise in free time (Active for more than twice a week)	ExerOft	
	How many hours usually exercise in free (Active for an hour or more)	ExerHrs	
**Sleeping habits [8 9]**	**Sleeping frequency in last week**		
	How often slept 8+ hours per night in last 7 days	Sleep8pn	The question was “Over the past 7 days, how often did you sleep for 8 hours or more” and the responses were “every day”, “most days”, “some days” and “not in the past 7 days”.

Confidential: For Review Only

**Eating Habits [10-12]**	**Definition of each unhealthy eating habits were:**		**Breakfast consumption + take away food + 5 A day**
	How often eaten take-away food in last 7 days (unhealthy habit if taken takeaway in past seven days)	Eattake	(1) Skipping Breakfast: If avoided breakfast in last 7 days (2) Poor diet: If consumed less than 5 portions of fruits and vegetables a day (3) Takeaway food: If consumed takeaway food in past 7 days. Each item was coded 0 for "no" and 1 for "yes". According to the number of unhealthy eating habits participant's exhibited, a risk score from zero to three (0= None, 1= Only one, 2 =Any two and 3 =All three) was given
	How often eaten breakfast in last 7 days (unhealthy habit if breakfast not taken regularly)	Eatbfst	
	Consumed more than five portions of fruit and veg yesterday (unhealthy habit if 5-A-DAY not taken regularly)	FV5ormor	
**Screen time [13-16]**	**Definitions of screen time derived as follows (Weekend and weekday hours combined)**		**TV +_computer games + internet**
	Hours per day watching TV	Watchwk, Watchwe	FOR ALL, Response options for each item were 'none at all', 'about half an hour a day', 'about 1 hour a day', 'about 2 hours a day', 'about 3 hours a day', 'about 4 hours a day', 'about 5 hours a day', 'about 6 hours a day' and 'about 7 or more hours a day'. The total screen time was calculated by grouping the similar options of each item and by combining both weekends and weekdays. Subjects were then categorised into "≥7hrs/day", "About 5-6 hours/day", "About 3 -4 hours/day", "About 2 hrs/day" and "≤1hrs/day".
	Hours per day spent playing computer games	Compwk , Compwe	
	Hours per day spent on computers for communicating, or homework	Comphwk, Comphwe	
	Hours per day spent on smart phone	Smartwk, Smartwe	
**Reading [17]**	**Definitions of reading derived as follows:**		**Reading hours (Weekend + weekdays)**
	Hours per day spent reading outside school hours	Readwk, Readwe	"About how many hours a day do you usually spend sitting down reading books, magazines or newspapers (including e-readers, online newspapers, and magazines) and studying when you are not at school?". The questions were asked for weekends and weekdays separately. The response options ranged from none to ≥ 7 hours per day
**Bullying [18 19]**	**Frequency with which the behaviour occurred over the past couple of months:**		**Traditional bullying (physical+ verbal + relational)**

Confidential: For Review Only

I was called mean names, was made fun of, or teased in a hurtful way	Bllynam	Bullying measure was meant to assess the extent to which they had recently experienced other children directing different types of bullying towards themselves. Six statements reflecting traditional bullying (physical, verbal, and relational) were asked. Questions were rated by participants using a 5-point response scale that ranged from 0="I haven't been bullied this way in the past couple of months" 1="It has happened once or twice", 2="2 or 3 times a month", 3="2 or 3 times a week", to 4="Several times a week."
Other people left me out of things on purpose, excluded me from their group of friends, or completely ignored me	Bllyout	
I was hit, kicked, pushed, shoved around, or locked indoors"	Bllyhit	
Other people lied or spread humours	Bllylie	
Made fun of me because of weight	Bllyfun	
Sexual jokes/comments	Bllysex	

Confidential: For Review Only

1. Farmer S, Hanratty B. The relationship between subjective wellbeing, low income and substance use among schoolchildren in the north west of England: a cross-sectional study. *Journal of Public Health* 2012;34(4):512-22. doi: 10.1093/pubmed/fds022
2. Phillips-Howard PA, Bellis MA, Briant LB, et al. Wellbeing, alcohol use and sexual activity in young teenagers: findings from a cross-sectional survey in school children in North West England. *Substance abuse treatment, prevention, and policy* 2010;5:27. doi: 10.1186/1747-597x-5-27 [published Online First: 2010/11/12]
3. Newcomb MD, Bentler PM, Collins C. Alcohol Use and Dissatisfaction with Self and Life: A Longitudinal Analysis of Young Adults. *Journal of Drug Issues* 1986;16(4):479-94. doi: 10.1177/002204268601600401
4. Bergman MM, Scott J. Young adolescents' wellbeing and health-risk behaviours: gender and socio-economic differences. *Journal of Adolescence* 2001;24(2):183-97. doi: https://doi.org/10.1006/jado.2001.0378
5. Taylor G, McNeill A, Girling A, et al. Change in mental health after smoking cessation: systematic review and meta-analysis. *BMJ : British Medical Journal* 2014;348 doi: 10.1136/bmj.g1151
6. Booker CL, Skew AJ, Sacker A, et al. Well-Being in Adolescence—An Association With Health-Related Behaviors:Findings From Understanding Society, the UK Household Longitudinal Study. *The Journal of Early Adolescence* 2014;34(4):518-38. doi: 10.1177/0272431613501082
7. Ussher MH, Owen CG, Cook DG, et al. The relationship between physical activity, sedentary behaviour and psychological wellbeing among adolescents. *Social Psychiatry and Psychiatric Epidemiology* 2007;42(10):851-56. doi: 10.1007/s00127-007-0232-x
8. Fuligni AJ, Hardway C. Daily Variation in Adolescents' Sleep, Activities, and Psychological Well-Being. *Journal of Research on Adolescence* 2006;16(3):353-78. doi: 10.1111/j.1532-7795.2006.00498.x
9. James S, Hale L. Sleep Duration and Child Wellbeing: A Nonlinear Association. *Journal of clinical child and adolescent psychology : the official journal for the Society of Clinical Child and Adolescent Psychology, American Psychological Association, Division 53* 2017;46(2):258-68. doi: 10.1080/15374416.2016.1204920
10. Rooney C, McKinley MC, Woodside JV. The potential role of fruit and vegetables in aspects of psychological well-being: a review of the literature and future directions. *Proceedings of the Nutrition Society* 2013;72(4):420-32. doi: 10.1017/S0029665113003388 [published Online First: 09/11]
11. Conner TS, Brookie KL, Carr AC, et al. Let them eat fruit! The effect of fruit and vegetable consumption on psychological well-being in young adults: A randomized controlled trial. *PLoS ONE* 2017;12(2):e0171206. doi: 10.1371/journal.pone.0171206
12. Lien L. Is breakfast consumption related to mental distress and academic performance in adolescents? *Public Health Nutrition* 2007;10(4):422-28. doi: 10.1017/S1368980007258550 [published Online First: 04/01]
13. Hoare E, Milton K, Foster C, et al. The associations between sedentary behaviour and mental health among adolescents: a systematic review. *International Journal of Behavioral Nutrition and Physical Activity* 2016;13(1):108. doi: 10.1186/s12966-016-0432-4
14. Przybylski AK, Weinstein N. A Large-Scale Test of the Goldilocks Hypothesis:Quantifying the Relations Between Digital-Screen Use and the Mental Well-Being of Adolescents. *Psychological Science* 2017;28(2):204-15. doi: 10.1177/0956797616678438

Confidential: For Review Only

15. Hamer M, Yates T, Sherar LB, et al. Association of after school sedentary behaviour in adolescence with mental wellbeing in adulthood. *Preventive Medicine* 2016;87(Supplement C):6-10. doi: https://doi.org/10.1016/j.ypmed.2016.02.021
16. Booker CL, Skew AJ, Kelly YJ, et al. Media Use, Sports Participation, and Well-Being in Adolescence: Cross-Sectional Findings From the UK Household Longitudinal Study. *American Journal of Public Health* 2015;105(1):173-79. doi: 10.2105/AJPH.2013.301783
17. Trainor S, Delfabbro P, Anderson S, et al. Leisure activities and adolescent psychological well-being. *Journal of Adolescence* 2010;33(1):173-86. doi: https://doi.org/10.1016/j.adolescence.2009.03.013
18. Landstedt E, Persson S. Bullying, cyberbullying, and mental health in young people. *Scandinavian journal of public health* 2014;42(4):393-9. doi: 10.1177/1403494814525004 [published Online First: 2014/03/13]
19. Przybylski AK, Bowes L. Cyberbullying and adolescent well-being in England: a population-based cross-sectional study. *The Lancet Child & Adolescent Health* 2017;1(1):19-26. doi: 10.1016/S2352-4642(17)30011-1

Confidential: For Review Only

BMJ Paediatrics Open

The Impact of Health Behaviours and Deprivation on Wellbeing in a National Sample of English Young People

Journal:	*BMJ Paediatrics Open*
Manuscript ID	bmjpo-2018-000335.R1
Article Type:	Original article
Date Submitted by the Author:	12-Jul-2018

Complete List of Authors:	Gireesh, Aswathikutty; University College London, Institute of Child Health<br>Das, Shikta; University College London, Institute of Cardiovascular Science, MRC Unit for Lifelong Health and Ageing<br>Viner, Russell; UCL Institute of Child Health, Population, Policy & Practice Research Programme

Keywords:	Adolescent Health, Sleep, Nutrition

SCHOLARONE™
Manuscripts

Confidential: For Review Only

TITLE

The Impact of Health Behaviours and Deprivation on Wellbeing in a National Sample of English Young People

Aswathikutty Gireesh MSc. [a]*

Shikta Das PhD [a]*

Prof. Russell Viner M.B.B.S., PhD [b]

*Joint first authors

[a] UCL Great Ormond Street Institute of Child Health, Population, Policy and Practice Programme, 30 Guilford Street, London WC1N 1EH, United Kingdom

[b] UCL Institute of Cardiovascular Science, MRC Unit for Lifelong Health and Ageing at UCL

Address for correspondence:

Aswathikutty Gireesh

Great Ormond Street Institute of Child Health, Population, Policy and Practice Programme. 30, Guilford Street, London WC1N 1EH, UK.

Email address: a.aswathikutty-gireesh@ucl.ac.uk

Word count: 2574

Confidential: For Review Only

ABSTRACT

Objective: To determine the malleable factors influencing wellbeing in boys and girls by accounting for deprivation, ethnicity and clustering within local authorities.

Methods: We used data from a very large nationally-representative survey, the What About Youth (WAY) study involving 120,115 adolescents aged 15 years. Our outcome measure of mental wellbeing was the Warwick-Edinburgh Mental Wellbeing Scale (WEMWBS). Potential explanatory factors included substance abuse, screen time, eating habits, reading, bullying, sleeping pattern, physical activity and area-level deprivation. We ran unadjusted and adjusted multilevel models for each explanatory factor, after adjusting for ethnicity, deprivation and including a random effect for the local authority.

Results: Boys had a higher overall mean WEMWBS score than girls ($p < 0.0001$). In the adjusted model, each of multiple risk behaviours, eating habits, sleep, bullying, physical activity, screen-time and reading were independently associated with mental wellbeing in both boy and girls ($P<0.0001$ for both). Sleep and eating behaviours had a stronger effect in both sexes than bullying, physical activity and screen time.

Conclusion: The largest contributors to adolescent wellbeing appear to be sleep, eating behaviours and bullying when considered in a multivariable framework. Future longitudinal studies and health policies need to consider a range of behavioural factors to drive improve improvements in adolescent wellbeing.

Confidential: For Review Only

INTRODUCTION

There are growing concerns about the wellbeing of young people in modern societies particularly in the UK where there is evidence that young people's wellbeing is lower than in many comparable developed countries [1 2].

Wellbeing is defined as “the state of being comfortable, healthy or happy” [3]. From a holistic perspective, wellbeing incorporates different dimensions of adolescent lives including social relationships as well as individual functioning [4]. However, the determinants of adolescent wellbeing is a relatively understudied area in comparison with the large literature on factors associated with mental health problems, as wellbeing concept has only been on greater focus over the past two decades [5 6].

A wide range of factors have been shown to be related to adolescent wellbeing, including a range cognitive and relational factors such as bullying [7], family structure and relationships [8], peer support [9] and school connectedness [10]. Other behaviours also influence wellbeing, including substance use (alcohol, drugs and smoking habits) [11-13], fruit and vegetable consumption [14], breakfast consumption [15], physical activity [16], sleep duration [17], sedentary behaviour [7 18] and leisure time activities [19]. However, published studies use a wide range of wellbeing measures, resulting in conflicting findings [20 21]. Furthermore, studies have largely focused on single risk factors and not explored how factors including behavioural factors interact to influence wellbeing. Additionally, given that many such behaviours are strongly socially patterned, studies have thus far paid little attention to confounding by socioeconomic position and issues relating to the clustering of behaviours and wellbeing within localities.

Policy initiatives to improve wellbeing amongst young people have largely focussed upon cognitive and psychological factors related to resilience to adversity, and have paid little attention to the contribution of non-psychological modifiable factors, such as other lifestyle behaviours. Understanding the potential contribution of modifiable behavioural factors to adolescent wellbeing may inform different strategies to improve young people's wellbeing.

Confidential: For Review Only

We used a very large recent nationally-representative and population-based survey of English 15 year-olds to examine the contribution of individual-level modifiable behaviours to wellbeing, including potentially protective (sleep, reading, and physical activity) and risk behaviours (substance use, unhealthy eating habits and excessive screen time). Our objective was to identify modifiable behavioural factors for mental wellbeing in boys and girls using an adolescent-specific measure and accounting for deprivation, ethnicity and clustering within local authorities (LAs).

Confidential: For Review Only

METHODS

Study design and sample

The What About Youth study (WAY) is a large-scale youth-oriented survey funded by the Department of Health (DH) in England and carried out by NHS Digital in 2014 [22]. The primary aim of the survey was to collect robust local authority level data on youth health behaviours and general health to improve their health outcomes. Study participants were those who turned 15 years old in the academic year 2013/14. A random sampling methodology was employed to draw 298,080 participants from the National Pupil Database. The sample size was calculated to attain 1000 young people in each of 152 LAs in England; 2 LAs were merged with their nearest neighbours due to small size.

The achieved sample was 120,115 individuals, of whom 16% responded online and 84% via postal means (2,835 opted out). The response rates differed by gender, with adjusted response rates of 35% in boys and 49% in girls, and by deprivation, ethnicity and LA. Non-response weights using these factors were calculated to provide alignment between the achieved and target samples [22]. We obtained a fully anonymised cohort data electronically from the United Kingdom (UK) Data Service website[22]. The National Children's Bureau (NBC) carried out a detailed ethical review of the original study, but it was not required for this secondary data analysis.

Outcome variable

Mental wellbeing was measured using the Warwick-Edinburgh Mental Wellbeing Scale (WEMWBS) [23], a population-level wellbeing measure. It is validated to use in adolescents' aged 13 or more and focus primarily on positive aspects of mental wellbeing (Internal consistency, α=0.90). Participants indicate how often they feel like each of the 14 items using a 5-point scale that ranges from 5 "all the time" to 1 "none of the time" [24]. Total scores ranged from 14-70 and were calculated by summing each participant's responses. The potential explanatory behavioural variables were identified from the literature review of previous publication. A detail description of each variable is given in Appendix A.

A composite variable for risk behaviour index for substance use was constructed by the summation of three dichotomous risk behaviour variables: (a) Smoking: If currently smokes (b) Drinking alcohol: If drinks once a month or more frequently (c) Cannabis use: If ever tried cannabis. Based on the number of risk behaviours, we categorised it from "none" to "three". Similarly, composite unhealthy eating habit index was derived from (a) Skipping Breakfast: If avoided breakfast in last 7 days (b) Poor diet: If consumed less than 5 portions of fruits and vegetables a day (c) Takeaway food: If consumed takeaway food in past 7 days. Based on a combination of unhealthy behaviours, the composite score was categorised into 0= none, 1= only one, 2 =any two and 3 =all three. Physical active for 60+ minutes for at least 5 days were classified as "physically active" and the rest "physically inactive." This threshold was defined in line with government recommendations [25 26], except for the intensity of exercise which was not available in the dataset. The selected threshold was taken at five days a week, as only 13% reported being physically active for seven days a week. A digital screen time variable was computed based on reported weekend and weekday usage of television, internet, smartphone and computer games. Subjects were categorised into "≥7hrs/day", "About 5-6 hours/day", "About 3 -4 hours/day", "About 2 hrs/day" and "≤1hrs/day". Time spent reading on weekends and weekdays had response options ranging

Confidential: For Review Only

from none to seven hours per day. Based on the distribution of data, we recoded the variable as "none", about half an hour/day", "about 1 hour/day" and "2 hours /day". The frequency of eight hours sleep in the last seven days was coded as "every day", "most days", "some days" and "not in the past 7 days". Bullying was measured with Olweus Bully/Victim Questionnaire, a reliable 8-item scale used to assess the bullying victimisation [27]. We combined responses to create one overall measure of bullying experience (yes/no). In line with a previous study [28], adolescents who were bullied more than "two or three times a month" were categorised as bullying victims.

Ethnicity, deprivation and mode of questionnaire completion were selected as confounders in the relationship between wellbeing and potential explanatory variables as shown in previous studies [7]. Ethnicity was self-identified by participants' and was an adaptation of the 2001 UK census categories, supplemented by questions on the national group. English Index of Multiple Deprivation (IMD) was used as a measure of relative deprivation for small areas [29]. IMD scores were divided into three deprivation categories as defined by quintiles of the national distribution: 1 and 2 (high deprivation), 3 (average), 4 and 5 (low). Participants were allowed to choose between online or postal modes of questionnaire completion.

Analyses

We conducted unadjusted and adjusted multilevel regression, in Stata 14 (Stata Corp, College Station, TX). For wellbeing scores, the interaction between gender and health behaviours was statistically significant ($p<0.001$) and therefore, analyses were stratified by gender. All variables were plotted to check the distribution and normality was checked with the Kolmogorov-Smirnov (K-S) test. All estimates were weighted by representativeness of participants to compensate for the disproportionate selection of sample and non-response bias. Pearson $\chi 2$ tests were used to compare differences in the distribution of explanatory variables by gender. An unadjusted analysis was run to test the association between each

Confidential: For Review Only

independent variable (substance use, unhealthy eating habits, screen time, reading, bullying, physical activity and sleeping hours) and the outcome adolescent wellbeing (Model 1). The analyses were repeated in a multivariable analysis where ethnicity, mode of questionnaire delivery and IMD were added as confounders between each risk factor and outcome (Model 2). In model 3, all explanatory factors and covariates were included simultaneously to obtain associations between each variable and wellbeing scores after adjusting for confounders and other explanatory variables. LAs were treated as random effects in all models. Models were also tested for significant quadratic terms signifying curvilinear relationships; this was only significant for reading. The intraclass correlations coefficient (ICC), being the proportion of total variance attributable to differences at the LA level, was estimated using multilevel models for wellbeing with adjustment for IMD, ethnicity and mode of completion.

Results

In total, 57,153 boys (47.82%) and 62,962 girls (52.18%) participated in the study (Table A1). Of these, boys had higher average wellbeing score in comparison to girls. The intracluster correlation coefficient (ICC) for the wellbeing score was 0.032 for girls and 0.024 for boys in the adjusted model, suggesting that variance in adolescent wellbeing is small at LA level.

Table A1 shows that there were significant differences in the distribution of potential explanatory variables between boys and girls. Girls had higher risk factors such as substance use, unhealthy eating habits, screen time and reported more bullying than boys. Protective variables such as sleeping hours (more than 8 hours) and reading were also significantly higher in girls than in boys.

Table A1. Descriptive statistics for well-being scores and explanatory variables under study, by gender

	Total	Boys		Girls		
	N	N	Mean (SD) / %	N	Mean (SD) / %	P value

Confidential: For Review Only

	**120,115**	**57,153**	**47.58%**	**62,962**	**52.42%**	
**WEMWBS Scores** [a]	117,842	56,352	47.82%	61,490	52.18%	<0.0001
Mean (SD)			50 (8.60)		45 (9.66)	
**Substance use** [b]	71,133	32,516	45.71%	38617	54.29%	<0.0001
None		17,133	52.69	19932	51.61	
One		10,783	33.16	12150	31.46	
Two		3,010	9.26	3,785	9.8	
Three		1,590	4.89	2,750	7.12	
**Unhealthy Eating Habits** [c]	115918	55,289	47.70%	60,629	52.30%	<0.0001
None		12,081	21.85	11,951	19.71	
One		20,287	36.69	20,389	33.63	
Two		16,373	29.61	19,064	31.44	
Three		6,548	11.84	9,225	15.22	
**Sleeping hours (>8 hrs)**	117,516	56, 207	47.83%	61307	52.17%	<0.0001
Not in the past 7 days		3676	6.54	6922	11.29	
some days		11051	19.66	16051	26.18	
most days		20, 121	35.8	20928	34.14	
everyday		21, 361	38	17406	28.39	
**Bullying**	117,744	56,309	47.82%	61,435	52.18%	<0.0001
No		45,959	81.62	45,094	73.40	
yes		10,350	18.38	16,341	26.60	
**Physical activity**	118450	56,674	47.85	61776	52.15	<0.0001
Physically active		48,172	85	44,348	71.79	
Inactive		8,502	15	17,428	28.21	
**Screen time**	118,845	56,892	47.87	61,943	52.13%	<0.0001
About 2 hrs/day		3,609	6.34	3,469	5.6	
≤1hrs/day		912	1.6	929	1.5	
About 3 -4 hours/day		18,621	32.73	17,311	27.95	
About 5-6 hours/day		17,198	30.23	17,850	28.82	
≥7hrs/day		16,559	29.1	22387	36.14	
**Reading**	118,140	56,513	47.84	61,627	52.16	<0.0001
None		14,278	25.26	9,875	16.02	
About Half an hour/day		17,572	31.09	15,363	24.93	
About 1 hr/day		12,505	22.13	13,442	21.81	

[a] WEMWBS : Warwick-Edinburgh Mental Wellbeing score
[b] Risk behaviours include smoking, drinking and cannabis use
[c] Unhealthy eating habits include skipping breakfast, not having 5 portions of fruits and vegetables and consumption of takeaway food.

All explanatory variables and IMD were significantly associated with wellbeing in both sexes (Table A2). Wellbeing in both sexes decreased with use of substance use, unhealthy eating habits, bullying, physical activity and longer screen time in both sexes. Protective factors,

Confidential: For Review Only

such as, sleeping more than eight hours and reading more than two hours were associated with higher wellbeing in both sexes.

Table A2: Univariate analysis between well-being and explanatory variables, by gender

	MODEL [1]			
	Boys		Girls	
	B	95 % CI	*B*	95 % CI
**Substance Use**				
None	Reference		Reference	
One	-0.34*	[-0.56,-0.13]	-1.48**	[-1.70,-1.27]
Two	-2.19**	[-2.60,-1.78]	-4.96**	[-5.35,-4.57]
Three	-3.56**	[-4.16,-2.95]	-5.89**	[-6.47,-5.30]
**Unhealthy Eating Habits**				
None	Reference		Reference	
One	-1.65**	[-1.88,-1.43]	-2.63**	[-2.91,-2.34]
Two	-3.36**	[-3.60,-3.12]	-4.85**	[-5.13,-4.58]
Three	-5.00**	[-5.39,-4.61]	-6.49**	[-6.83,-6.14]
**Sleeping hours (>8 hrs)**				
Not in the past 7 days	Reference		Reference	
some days	2.99**	[2.51,3.47]	4.71**	[4.39,5.04]
most days	5.59**	[5.15,6.03]	8.39**	[8.06,8.72]
everyday	7.49**	[7.07,7.87]	10.70**	[10.35,11.06]
**Bullying**				
No	Reference		Reference	
Yes	-4.70**	[-3.59,-3.31]	-5.84**	[-5.25,-4.90]
**Physical activity**				
Physically active	Reference		Reference	
Inactive	-3.85**	[-4.11,-3.61]	-2.77**	[-3.04,-2.51]
**Screen time**				
About 2 hrs/day	Reference		Reference	
≤1hrs/day	0.07	[-0.59, 0.73]	0.08	[-0.69, 0.85]
About 3 -4 hours/day	-1.22**	[-1.60,-0.84]	-1.27**	[-1.71,-0.83]
About 5-6 hours/day	-2.17**	[-2.51,-1.83]	-3.06**	[-3.52,-2.60]
>= 7 hours / day	-3.72**	[-4.12,-3.32]	-5.38**	[-5.80,-4.97]
**Reading**				
None	Reference		Reference	
About Half an hour/day	1.61**	[1.41,1.82]	2.17**	[1.88,2.46]
About 1 hr/day	2.25**	[2.06,2.44]	2.54**	[2.26,2.82]
≥2hrs/day	2.27***	[2.01,2.52]	2.35**	[2.05,2.66]
**IMD Scores**				
High deprivation	Reference		Reference	
Average deprivation	0.57**	[0.31,0.81]	0.35**	[0.10,0.59]

Confidential: For Review Only

Least deprivation	1.05**	[0.87,1.21]	1.16**	[0.92,1.40]
**Ethnicity**				
White	Reference		Reference	
Mixed	0.18	[-0.29,0.65]	-0.44*	[-0.82,-0.06]
Asian	-0.54*	[-0.85,-0.23]	0.98**	[0.59,1.38]
Black	0.91**	[0.42,1.39]	1.10**	[0.67,1.52]
Other	-0.45*	[-0.84,-0.04]	0.30	[-0.09,0.70]

[1]Unadjusted model taking into account clustering at Local Authority Level, Multilevel models fitted with weighted design weights, Quadratic function added to reading.

** p<0.001, * p<0.05

In the multivariable models adjusted for covariates (Table A3), poorer wellbeing was associated with multiple substances use and multiple unhealthy eating habits in a dose-dependent fashion. Being physically inactive, longer screen time and experiencing bullying were both associated with decrements in wellbeing in both sexes, with the association being stronger in girls than in boys.

Confidential: For Review Only

Table A3: Gender - stratified partially-adjusted and fully-adjusted multilevel modelling for well-being and explanatory variables

	Boys				Girls			
	MODEL[2]		MODEL[3]		MODEL[2]		MODEL[3]	
**Variables**	B	95 % CI	B	95 % CI	B	95 % CI	B	95 % CI
**Substance use**								
None	Reference		Reference		Reference		Reference	
One	-0.40**	[-0.61,-0.18]	-0.14	[-0.36,0.08]	-1.51**	[-1.73,-1.30]	-0.77**	[-0.97,-0.57]
Two	-2.16**	[-2.57,-1.75]	-1.05**	[-1.42,-0.67]	-4.84**	[-5.22,-4.46]	-2.67**	[-3.01,-2.33]
Three	-3.50**	[-4.11,-2.88]	-1.63**	[-2.16,-1.09]	-5.80**	[-6.36,-5.23]	-2.79**	[-3.35,-2.24]
**Eating Habits**								
None	Reference		Reference		Reference		Reference	
One	-1.63**	[-1.85,-1.41]	-0.89**	[-1.18,-0.60]	-2.63**	[-2.92,-2.34]	-1.37**	[-1.69,-1.06]
Two	-3.31**	[-3.55,-3.08]	-1.84**	[-2.14,-1.54]	-4.84**	[-5.12,-4.55]	-2.29**	[-2.64,-1.96]
Three	-4.95**	[-5.34,-4.56]	-2.44**	[-2.82,-2.06]	-6.46**	[-6.79,-6.10]	-2.61**	[-3.04,-2.18]
**Sleeping hours (>8 hrs)**								
Not in the past 7 days	Reference		Reference		Reference		Reference	
some days	2.98**	[2.50,3.45]	2.69**	[2.10,3.28]	4.69**	[4.36,5.01]	3.71**	[3.34,4.08]
most days	5.55**	[5.11,5.99]	4.30**	[3.78,4.82]	8.35**	[8.01,8.69]	6.64**	[6.28,7.00]
everyday	7.45**	[7.03,7.86]	5.79**	[5.29,6.28]	10.65**	[10.29,11.01]	8.16**	[7.72,8.60]
**Bullying**								
No	Reference		Reference		Reference		Reference	
Yes	-4.64**	[-4.86,-4.42]	-3.78**	[-4.09,-3.48]	-5.77**	[-5.98,-5.56]	-4.01**	[-4.23,-3.78]
**Physical activity**								
Physically active	Reference		Reference		Reference		Reference	
Inactive	-3.78**	[-4.02,-3.55]	-2.63**	[-2.95,-2.30]	-2.77**	[-3.04, -2.50]	-1.70**	[-2.01,-1.39]
**Screen time**								
About 2 hrs/day	Reference		Reference		Reference		Reference	
≤1hrs/day	0.09	[-0.57,0.75]	0.34	[-0.58,1.26]	0.09	[-0.69,0.87]	-0.38	[-1.49,0.72]
About 3 -4 hours/day	-1.22**	[-1.60,-0.83]	-0.61*	[-0.99,-0.23]	-1.26**	[-1.70,-0.83]	-0.54	[-1.14,0.05]
About 5-6 hours/day	-2.15**	[-2.50,-1.81]	-0.82**	[-1.27,-0.37]	-3.04**	[-3.49,-2.58]	-1.21**	[-1.75,-0.67]
>= 7 hours / day	-3.67**	[-4.06,-3.27]	-1.20**	[-1.65,-0.75]	-5.32**	[-5.73,-4.90]	-1.81**	[-2.29,-1.33]

Confidential: For Review Only

2

**Reading**								
None	Reference		Reference		Reference		Reference	
About Half an hour/day	1.59**	[1.38,1.80]	0.57**	[0.26,0.88]	2.13**	[1.84,2.42]	0.56**	[0.23,0.90]
About 1 hr/day	2.24**	[2.04,2.43]	1.04**	[0.79,1.29]	2.51**	[2.24,2.78]	0.60**	[0.26,0.93]
≥2hrs/day	2.28**	[2.05,2.54]	0.90**	[0.55,1.25]	2.34**	[2.03,2.64]	0.33*	[0.02,0.64]
**IMD Scores**								
High deprivation	--	--	Reference		--	--	Reference	
Average deprivation	--	--	-0.12	[-0.38,0.14]	--	--	0.16	[-0.16,0.49]
Least deprivation	--	--	0.17	[-0.06,0.40]	--	--	0.36*	[0.06,0.66]
**Ethnicity**								
White	--	--	Reference		--	--	Reference	
Mixed	--	--	-0.06	[0.81,-0.59]	--	--	0.44	[-0.08,0.96]
Asian	--	--	-0.09	[0.81,-0.84]	--	--	0.35	[-0.53,1.23]
Black	--	--	0.99*	[0.01,0.25]	--	--	1.75**	[1.20,2.31]
Other	--	--	-0.59	[0.02,-1.09]	--	--	-0.19	[-0.88,0.49]
**Mode of questionnaire delivery**								
Online	--	--			--	--		
paper	--	--	0.24	[-0.03,0.51]	--	--	1.26**	[0.99,1.53]

[2] Each predictor variable adjusted for ethnicity, mode of questionnaire delivery and Index of multiple deprivation

[3] All Variables mutually adjusted for each other

Note: Multilevel models fitted with weighted design weights, Quadratic function added to reading.

** p<0.001, * p<0.05

Confidential: For Review Only

Higher wellbeing was associated with number of days young people achieved more than eight hours of sleep again in a dose-dependent fashion. Habitual reading most days was associated with higher wellbeing although there was no evidence of a dose-response above 30 minutes per day in the fully adjusted model (Figure 1). Young people from black ethnic groups had significantly higher wellbeing in both sexes. Deprivation was not associated with wellbeing amongst boys but was amongst girls.

Discussion

This study broadens our understanding of risk and protective factors associated with wellbeing in adolescence, using a very large nationally representative survey to examine a wide spectrum of behavioural and psychosocial factors relating to youth wellbeing and taking into account deprivation and clustering at LA level. The study shows that young people who reported lower levels of wellbeing were more likely to have engaged in multiple unhealthy eating habits and substance use; be victims of bullying; have exercised insufficiently; have exceeded recommended screen time use. These findings were robust to mutual adjustment for all variables and for deprivation, ethnicity, and mode of questionnaire delivery. A dose-response pattern was also observed between wellbeing and health behaviours such as substance use, unhealthy eating habits and sleeping pattern. A decrease in the number of risk behaviours and an increase in the number of days slept for more than eight hours, corresponded with an increase in average levels of wellbeing. The impact of deprivation on wellbeing was surprisingly small, as was LA locality variance, suggesting that variance in wellbeing lies largely in behavioural and psychological factors.

Since different studies have used different variables to define various aspects of wellbeing in the analyses, comparison with other studies is difficult. We found boys reported higher mean wellbeing than girls, consistent with national reports for England [30 31], but in contrast to findings from recent Health Survey England (HSE) 2015, where only slight gender variations were observed [32]. The proportions reporting each of the behaviours were broadly similar to those found in other recent national surveys [31 33 34]. Girls reported higher levels of risky

Confidential: For Review Only

health behaviours including current smoking, alcohol consumption, bullying, lower levels of physical activity, on the other hand, boys were more likely to report higher levels of physical activity and these were consistent with findings from the HBSC England [33].

These findings corroborate with two previous studies where happiness as a marker of wellbeing was found to be positively associated with multiple health protective behaviours (sport's participation and healthier eating) and negatively associated with multiple risk behaviours (smoking, alcohol use and heavy screen use) in adolescents [35 36]. Our finding that substance use was associated with lower wellbeing is similar to that seen in other studies, as were our findings for being bullied [7]. The association of sleep duration and reading with wellbeing in young people has been little studied. Leisure time and adequate sleep have been identified as being associated with wellbeing[17 19], however ours is the first to examine these alongside other behavioural and psychological factors.

We found an association between deprivation and lower wellbeing, although in contrast to previous studies[37] the effect size was small and we found no association in boys. This may reflect the lack of adjustment for multiple behaviours, ethnicity and area effects in other studies. Our finding suggests that much of what has previously been understood, as unhealthy behaviours themselves associated with deprivation may mediate deprivation effects. We found that young people black ethnic groups reported significantly higher wellbeing in both sexes, consistent with previous UK findings [38]. However, the reasons for this remain unclear and require further study. We found this association to be robust to adjustment for deprivation and all significant behavioural and psychological factors, suggesting this likely relates to factors not measured in our study.

We used a large, nationally representative sample of ethnically and socioeconomically diverse adolescents. Prior studies have examined very few health behaviours and relied on proxy measures of wellbeing rather than on population-level wellbeing measures that tap into

Confidential: For Review Only

both feelings and psychological flourishing. In our study, associations between wellbeing and behavioural factors were examined within a multivariable and multilevel framework, using a validated wellbeing scale with robust psychometric properties.

Our findings are subject to a number of limitations. Our data were cross-sectional and thus the direction of causality is unclear for the behavioural variables. Participant responses could be influenced by social desirability, and those with poor wellbeing may be inclined towards endorsing questions more than others, thus introducing bias. The direction of such biases is unclear, however, we note that girls (who had a higher response rate in the overall survey) reported higher levels of both more risky and protective behaviours than boys, potentially reflecting social desirability biases. All variables used were self-reported except for area-level deprivation. We have also repeated analysis excluding the outliers and that did not materially affect the findings. We used bullying victimization as a proxy for psychological problems due to the lack of more appropriate variables in the dataset; thus it is possible that some of the associations seen here result from inadequate adjustment for psychological issues. We combined variables across domains into composite variables; this may have introduced bias although the direction of bias is unclear.

Conclusion

Our findings suggest that promoting healthy sleep, reading, and healthy eating behaviours may present important policy targets for enhancing adolescent wellbeing in addition to more accepted foci on physical activity, screen time and bullying. Future work is needed to examine these factors within a longitudinal causal framework**.**

Confidential: For Review Only

What is already known on this topic?

Young people's wellbeing may be affected by multiple individual and contextual factors.

Key determinants of adolescent wellbeing remain unclear.

Few studies have examined a wide range of potential determinants whilst adjusting for area-level deprivation.

What this study adds?

Findings support current policy foci on bullying, physical activity and screen-time as correlates of wellbeing amongst young people.

Sleep and eating behaviours may also be important policy targets for promoting adolescent wellbeing.

A coherent policy framework to promote adolescent wellbeing needs to be multifaceted and consider a range of health factors in young people's lives

Confidential: For Review Only

Funding Sources

This report is independent research commissioned and funded by the National Institute for Health Research Policy Research Programme. The views expressed in this publication are those of the author(s) and not necessarily those of the NHS, the National Institute for Health Research, the Department of Health or its arm's length bodies, and other Government Departments.

Conflicts of interests

The authors have no conflicts of interest to disclose

Contributors

AG & SD led the writing of the study and undertook the analyses. RV conceptualised the project, contributed to the ideas underlying the article, contributed to writing and revision of the article.

Acknowledgement

Preliminary findings of this paper were presented at the Adolescent Lives and Wellbeing 2017 Conference organised by University College London.

Confidential: For Review Only

References

1. Patton GC, Sawyer SM, Santelli JS, et al. Our future: a Lancet commission on adolescent health and wellbeing. *Lancet (London, England)* 2016;387(10036):2423-78. doi: 10.1016/s0140-6736(16)00579-1 [published Online First: 2016/05/14]

2. RCPCH. State of Child Health Report Royal College of Paediatrics and Child Health; 2017 [Available from: http://www.thehealthwell.info/node/1062390 accessed 6th November 2017.

3. Oxford University Press. Oxford Dictionary. Oxford: Oxford University Press 2016.

4. Žukauskienė R. Adolescence and Well-Being. In: Ben-Arieh A, Casas F, Frønes I, et al., eds. Handbook of Child Well-Being: Theories, Methods and Policies in Global Perspective. Dordrecht: Springer Netherlands 2014:1713-38.

5. Das JK, Salam RA, Lassi ZS, et al. Interventions for Adolescent Mental Health: An Overview of Systematic Reviews. *Journal of Adolescent Health* 2016;59(4, Supplement):S49-S60. doi: https://doi.org/10.1016/j.jadohealth.2016.06.020

6. Keyes CL. Promoting and protecting mental health as flourishing: a complementary strategy for improving national mental health. *The American psychologist* 2007;62(2):95-108. doi: 10.1037/0003-066x.62.2.95 [published Online First: 2007/02/28]

7. Przybylski AK, Bowes L. Cyberbullying and adolescent well-being in England: a population-based cross-sectional study. *The Lancet Child & Adolescent Health* 2017;1(1):19-26. doi: 10.1016/S2352-4642(17)30011-1

8. Dinisman T, Andresen S, Montserrat C, et al. Family structure and family relationship from the child well-being perspective: Findings from comparative analysis. *Children and Youth Services Review* 2017;80(Supplement C):105-15. doi: https://doi.org/10.1016/j.childyouth.2017.06.064

9. Raja SN, McGee R, Stanton WR. Perceived attachments to parents and peers and psychological well-being in adolescence. *Journal of Youth and Adolescence* 1992;21(4):471-85. doi: 10.1007/bf01537898

10. García Bacete FJ, Marande Perrin G, Schneider BH, et al. Effects of School on the Well-Being of Children and Adolescents. In: Ben-Arieh A, Casas F, Frønes I, et al., eds. Handbook of Child Well-Being:

Confidential: For Review Only

Theories, Methods and Policies in Global Perspective. Dordrecht: Springer Netherlands 2014:1251-305.

11. Taylor G, McNeill A, Girling A, et al. Change in mental health after smoking cessation: systematic review and meta-analysis. *BMJ : British Medical Journal* 2014;348 doi: 10.1136/bmj.g1151

12. Farmer S, Hanratty B. The relationship between subjective wellbeing, low income and substance use among schoolchildren in the north west of England: a cross-sectional study. *Journal of Public Health* 2012;34(4):512-22. doi: 10.1093/pubmed/fds022

13. Phillips-Howard PA, Bellis MA, Briant LB, et al. Wellbeing, alcohol use and sexual activity in young teenagers: findings from a cross-sectional survey in school children in North West England. *Substance abuse treatment, prevention, and policy* 2010;5:27. doi: 10.1186/1747-597x-5-27 [published Online First: 2010/11/12]

14. Conner TS, Brookie KL, Carr AC, et al. Let them eat fruit! The effect of fruit and vegetable consumption on psychological well-being in young adults: A randomized controlled trial. *PLoS ONE* 2017;12(2):e0171206. doi: 10.1371/journal.pone.0171206

15. Lien L. Is breakfast consumption related to mental distress and academic performance in adolescents? *Public Health Nutrition* 2007;10(4):422-28. doi: 10.1017/S1368980007258550 [published Online First: 04/01]

16. Ussher MH, Owen CG, Cook DG, et al. The relationship between physical activity, sedentary behaviour and psychological wellbeing among adolescents. *Social Psychiatry and Psychiatric Epidemiology* 2007;42(10):851-56. doi: 10.1007/s00127-007-0232-x

17. Fuligni AJ, Hardway C. Daily Variation in Adolescents' Sleep, Activities, and Psychological Well-Being. *Journal of Research on Adolescence* 2006;16(3):353-78. doi: 10.1111/j.1532-7795.2006.00498.x

18. Hamer M, Yates T, Sherar LB, et al. Association of after school sedentary behaviour in adolescence with mental wellbeing in adulthood. *Preventive Medicine* 2016;87(Supplement C):6-10. doi: https://doi.org/10.1016/j.ypmed.2016.02.021

19. Trainor S, Delfabbro P, Anderson S, et al. Leisure activities and adolescent psychological well-being. *Journal of Adolescence* 2010;33(1):173-86. doi: https://doi.org/10.1016/j.adolescence.2009.03.013

Confidential: For Review Only

20. Pollard EL, Lee PD. Child Well-being: A Systematic Review of the Literature. *Social Indicators Research* 2003;61(1):59-78. doi: 10.1023/a:1021284215801

21. Rose T, Joe S, Williams A, et al. Measuring Mental Wellbeing Among Adolescents: A Systematic Review of Instruments. *Journal of Child and Family Studies* 2017;26(9):2349-62. doi: 10.1007/s10826-017-0754-0

22. Health and Social Care Information Centre. What About YOUth? Survey. England: UK Data Service, 2016.

23. Tennant R, Hiller L, Fishwick R, et al. The Warwick-Edinburgh Mental Well-being Scale (WEMWBS): development and UK validation. *Health and Quality of Life Outcomes* 2007;5:63-63. doi: 10.1186/1477-7525-5-63

24. Taggart F, Stewart-Brown S, Parkinson J. Warwick-Edinburgh Mental Well-being Scale (WEMWBS) Scotland: NHS Health Scotland; 2015 [Available from: http://www.healthscotland.com/uploads/documents/26787-WEMWBS%20User%20Guide%20Version%202%20May%202015.pdf accessed 19th October 2017.

25. Morgan K, Hallingberg B, Littlecott H, et al. Predictors of physical activity and sedentary behaviours among 11-16 year olds: Multilevel analysis of the 2013 Health Behaviour in School-aged Children (HBSC) study in Wales. *BMC Public Health* 2016;16:569. doi: 10.1186/s12889-016-3213-8

26. Booth ML, Okely AD, Chey T, et al. The reliability and validity of the physical activity questions in the WHO health behaviour in schoolchildren (HBSC) survey: a population study. *British journal of sports medicine* 2001;35(4):263-7. [published Online First: 2001/07/31]

27. Olweus D. The Revised Olweus Bully/Victim Questionnaire. University of Bergen: Research Center for Health Promotion (HEMIL Center) 1996.

28. Cook CR, Williams KR, Guerra NG, et al. Predictors of bullying and victimization in childhood and adolescence: A meta-analytic investigation. *School Psychology Quarterly* 2010;25(2):65-83. doi: 10.1037/a0020149

29. Department for Communities and Local Government. The English indices of deprivation London2015 [Available from:

Confidential: For Review Only

https://www.gov.uk/government/uploads/system/uploads/attachment_data/file/465791/English_Indices_of_Deprivation_2015_-_Statistical_Release.pdf

30. Office of National Statistics. Measuring National Well-being: Insights into children's mental health and well-being. England, 2015.

31. Office of National Statistics. Young people's well-being England: Office for National Statistics; 2017 [Available from: https://www.ons.gov.uk/peoplepopulationandcommunity/wellbeing/articles/youngpeopleswellbeingandpersonalfinance/2017 accessed 10th November 2017.

32. Fuller E, Morris S. Health Survey for England. London NHS Digital, 2015.

33. Currie C, Grieber R, Inchley J. Health Behaviour in School-Aged Children (HBSC) Study Protocol: Background, Methodology and Mandatory Items for the 2009/10 Survey. Edinburgh: CAHRU 2010.

34. Brooks F, Magnusson J, Klemera E, et al. Health Behaviours of School-Aged Children (HBSC) 2013-2014 England2014 [Available from: www.hbscengland.com.

35. Booker CL, Skew AJ, Sacker A, et al. Well-Being in Adolescence—An Association With Health-Related Behaviors:Findings From Understanding Society, the UK Household Longitudinal Study. *The Journal of Early Adolescence* 2014;34(4):518-38. doi: 10.1177/0272431613501082

36. Booker CL, Skew AJ, Kelly YJ, et al. Media Use, Sports Participation, and Well-Being in Adolescence: Cross-Sectional Findings From the UK Household Longitudinal Study. *American Journal of Public Health* 2015;105(1):173-79. doi: 10.2105/AJPH.2013.301783

37. Candace Currie, Cara Zanotti, Antony Morgan, et al. Social determinants of health and well-being among young people. International report from the 2009/2010 survey. Health Behavior in school-age children (HBSC) study. Denmark: WHO Regional Office for Europe, 2012:252.

38. Patalay P, Fitzsimons E. Correlates of Mental Illness and Wellbeing in Children: Are They the Same? Results From the UK Millennium Cohort Study. *Journal of the American Academy of Child & Adolescent Psychiatry* 2016;55(9):771-83. doi: 10.1016/j.jaac.2016.05.019

.

Confidential: For Review Only

Confidential: For Review Only

Figure 1

Relationship between wellbeing and health behaviours: (A) Risk Factors (B) Protective Factors

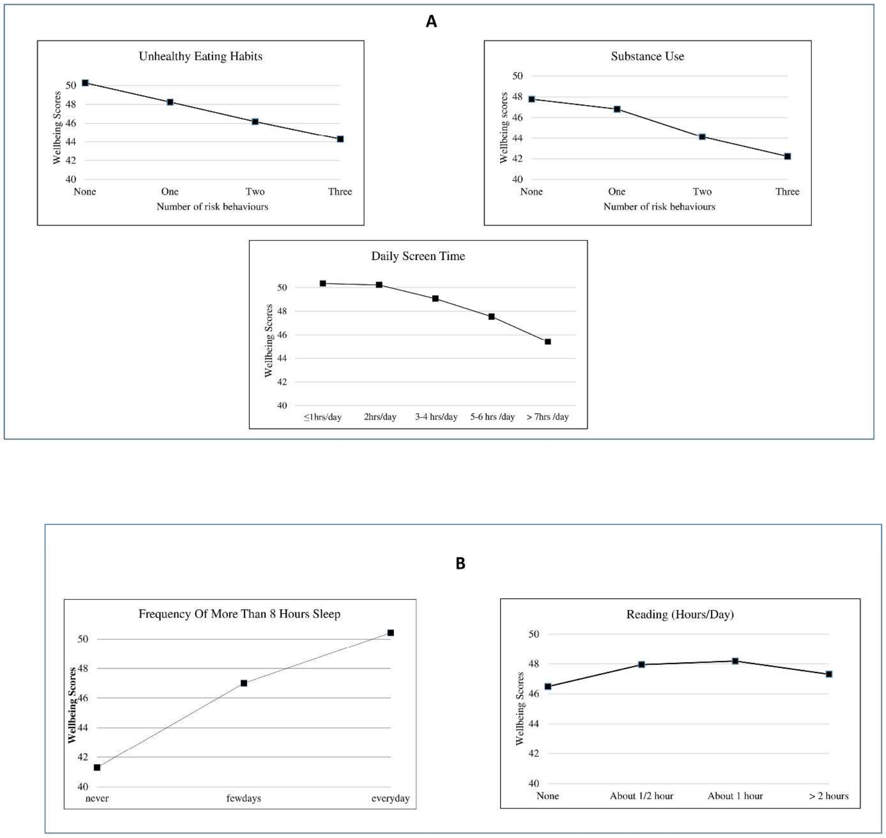

APPENDIX A

(i) VARIABLE DESCRIPTION

Composite variables	Original variables	Variable labels	Composite variables
**Substance use risk [1-6]**	**Definitions were:**		**Alcohol + smoking + cannabis**
	Smoking: Cigarette smoking status (currently smoke)	SmkSt2	(1) Smoking risk: If currently smokes (2) Drinking risk: If drinks once a month or more frequently (3) Cannabis risk: If ever tried cannabis. Each item was coded 0 for "no" and 1 for "yes". The composite score obtained ranged from zero to three based on the number of risk behaviours; (0= None, 1= Only one, 2 =Any two and 3 =All three
	Drinking: Alcohol drinking status (Usually have an alcoholic drink once a month or more frequently)	alcfrq1	
	Cannabis: Cannabis use status (Ever tried cannabis)	Cantry	
**Physical Activity [7 8]**	**Physical activity: in line with general recommendations**		**Duration of exercise + Frequency of exercise + Number of days met recommended guidelines**
	How many days physically active for 60+ minutes, in last 7 days (Active for more than 5 days)	Physact	The first question on moderate to vigorous physical activity was "Over the past 7 days, on how many days you were physically active for a total of at least 60 minutes per day and responses ranged from 0 to 7. Second, the participants were asked "Outside school hours: how often do you usually exercise in your free time so much that you get out of breath or sweat?" with responses from less than a once a month to every day. The question on duration was "Outside school hours: how many hours do you usually exercise in your free time so much that you get out of breath or sweat?", with responses from none to 7 or more hours. Adolescents who exercised for more than twice a week for an hour or more or were physically active for 60+ minutes for at least 5 days were classified as "physically active" and the rest "physically inactive".
	How often usually exercise in free time (Active for more than twice a week)	ExerOft	
	How many hours usually exercise in free (Active for an hour or more)	ExerHrs	
**Sleeping habits [9 10]**	**Sleeping frequency in last week**		
	How often slept 8+ hours per night in last 7 days	Sleep8pn	The question was "Over the past 7 days, how often did you sleep for 8 hours or more" and the responses were "every day", "most days", "some days" and "not in the past 7 days".

Confidential: For Review Only

**Eating Habits [11-13]**	**Definition of each unhealthy eating habits were:**		**Breakfast consumption + take away food + 5 A day**
	How often eaten take-away food in last 7 days (unhealthy habit if taken takeaway in past seven days)	Eattake	(1) Skipping Breakfast: If avoided breakfast in last 7 days (2) Poor diet: If consumed less than 5 portions of fruits and vegetables a day (3) Takeaway food: If consumed takeaway food in past 7 days. Each item was coded 0 for “no” and 1 for “yes”. According to the number of unhealthy eating habits participant’s exhibited, a risk score from zero to three (0= None, 1= Only one, 2 =Any two and 3 =All three) was given
	How often eaten breakfast in last 7 days (unhealthy habit if breakfast not taken regularly)	Eatbfst	
	Consumed more than five portions of fruit and veg yesterday (unhealthy habit if 5-A-DAY not taken regularly)	FV5ormor	
**Screen time [8 14-16]**	**Definitions of screen time derived as follows (Weekend and weekday hours combined)**		**TV +_computer games + internet**
	Hours per day watching TV	Watchwk, Watchwe	FOR ALL, Response options for each item were ‘none at all’, ‘about half an hour a day’, ‘about 1 hour a day’, ‘about 2 hours a day’, ‘about 3 hours a day’, ‘about 4 hours a day’, ‘about 5 hours a day’, ‘about 6 hours a day’ and ‘about 7 or more hours a day’. The total screen time was calculated by grouping the similar options of each item and by combining both weekends and weekdays. Subjects were then categorised into “≥7hrs/day”, “About 5-6 hours/day”, “About 3 -4 hours/day”, “About 2 hrs/day” and “≤1hrs/day”.
	Hours per day spent playing computer games	Compwk , Compwe	
	Hours per day spent on computers for communicating, or homework	Comphwk, Comphwe	
	Hours per day spent on smart phone	Smartwk, Smartwe	
**Reading [17]**	**Definitions of reading derived as follows:**		**Reading hours (Weekend + weekdays)**
	Hours per day spent reading outside school hours	Readwk, Readwe	“About how many hours a day do you usually spend sitting down reading books, magazines or newspapers (including e-readers, online newspapers, and magazines) and studying when you are not at school?”. The questions were asked for weekends and weekdays separately. The response options ranged from none to ≥ 7 hours per day
**Bullying [18 19]**	**Frequency with which the behaviour occurred over the past couple of months:**		**Traditional bullying (physical+ verbal + relational)**

Confidential: For Review Only

I was called mean names, was made fun of, or teased in a hurtful way	Bllynam	Bullying measure was meant to assess the extent to which they had recently experienced other children directing different types of bullying towards themselves. Six statements reflecting traditional bullying (physical, verbal, and relational) were asked. Questions were rated by participants using a 5-point response scale that ranged from 0="I haven't been bullied this way in the past couple of months" 1="It has happened once or twice", 2="2 or 3 times a month", 3="2 or 3 times a week", to 4="Several times a week."
Other people left me out of things on purpose, excluded me from their group of friends, or completely ignored me	Bllyout	
I was hit, kicked, pushed, shoved around, or locked indoors"	Bllyhit	
Other people lied or spread humours	Bllylie	
Made fun of me because of weight	Bllyfun	
Sexual jokes/comments	Bllysex	

(ii) Individual Items and scoring of Warwick-Edinburgh Mental Well-being scale (WEMWBS)

1. I've been feeling optimistic about the future<br>2. I've been feeling useful<br>3. I've been feeling relaxed<br>4. I've been feeling interested in other people<br>5. I've had energy to spare<br>6. I've been dealing with problems well<br>7. I've been thinking clearly<br>8. I've been feeling good about myself<br>9. I've been feeling close to other people<br>10. I've been feeling confident<br>11. I've been able to make up my own mind about things<br>12. I've been feeling loved<br>13. I've been interested in new things<br>14. I've been feeling cheerful	Each of the 14 item responses in WEMWBS are scored from 1 (none of the time), 2 (rarely), 3 (some of the time), 4 (often) to 5 (all of the time). A total scale score is calculated by summing the 14 individual item scores.

Confidential: For Review Only

REFERENCES

1. Farmer S, Hanratty B. The relationship between subjective wellbeing, low income and substance use among schoolchildren in the north west of England: a cross-sectional study. *Journal of Public Health* 2012;34(4):512-22. doi: 10.1093/pubmed/fds022
2. Phillips-Howard PA, Bellis MA, Briant LB, et al. Wellbeing, alcohol use and sexual activity in young teenagers: findings from a cross-sectional survey in school children in North West England. *Substance abuse treatment, prevention, and policy* 2010;5:27. doi: 10.1186/1747-597x-5-27 [published Online First: 2010/11/12]
3. Newcomb MD, Bentler PM, Collins C. Alcohol Use and Dissatisfaction with Self and Life: A Longitudinal Analysis of Young Adults. *Journal of Drug Issues* 1986;16(4):479-94. doi: 10.1177/002204268601600401
4. Bergman MM, Scott J. Young adolescents' wellbeing and health-risk behaviours: gender and socio-economic differences. *Journal of Adolescence* 2001;24(2):183-97. doi: https://doi.org/10.1006/jado.2001.0378
5. Taylor G, McNeill A, Girling A, et al. Change in mental health after smoking cessation: systematic review and meta-analysis. *BMJ : British Medical Journal* 2014;348 doi: 10.1136/bmj.g1151
6. Booker CL, Skew AJ, Sacker A, et al. Well-Being in Adolescence—An Association With Health-Related Behaviors:Findings From Understanding Society, the UK Household Longitudinal Study. *The Journal of Early Adolescence* 2014;34(4):518-38. doi: 10.1177/0272431613501082
7. Ussher MH, Owen CG, Cook DG, et al. The relationship between physical activity, sedentary behaviour and psychological wellbeing among adolescents. *Social Psychiatry and Psychiatric Epidemiology* 2007;42(10):851-56. doi: 10.1007/s00127-007-0232-x
8. Booker CL, Skew AJ, Kelly YJ, et al. Media Use, Sports Participation, and Well-Being in Adolescence: Cross-Sectional Findings From the UK Household Longitudinal Study. *American Journal of Public Health* 2015;105(1):173-79. doi: 10.2105/AJPH.2013.301783
9. Fuligni AJ, Hardway C. Daily Variation in Adolescents' Sleep, Activities, and Psychological Well-Being. *Journal of Research on Adolescence* 2006;16(3):353-78. doi: 10.1111/j.1532-7795.2006.00498.x
10. James S, Hale L. Sleep Duration and Child Wellbeing: A Nonlinear Association. *Journal of clinical child and adolescent psychology : the official journal for the Society of Clinical Child and Adolescent Psychology, American Psychological Association, Division 53* 2017;46(2):258-68. doi: 10.1080/15374416.2016.1204920
11. Rooney C, McKinley MC, Woodside JV. The potential role of fruit and vegetables in aspects of psychological well-being: a review of the literature and future directions. *Proceedings of the Nutrition Society* 2013;72(4):420-32. doi: 10.1017/S0029665113003388 [published Online First: 09/11]
12. Conner TS, Brookie KL, Carr AC, et al. Let them eat fruit! The effect of fruit and vegetable consumption on psychological well-being in young adults: A randomized controlled trial. *PLoS ONE* 2017;12(2):e0171206. doi: 10.1371/journal.pone.0171206
13. Lien L. Is breakfast consumption related to mental distress and academic performance in adolescents? *Public Health Nutrition* 2007;10(4):422-28. doi: 10.1017/S1368980007258550 [published Online First: 04/01]
14. Hoare E, Milton K, Foster C, et al. The associations between sedentary behaviour and mental health among adolescents: a systematic review. *International Journal of Behavioral Nutrition and Physical Activity* 2016;13(1):108. doi: 10.1186/s12966-016-0432-4
15. Przybylski AK, Weinstein N. A Large-Scale Test of the Goldilocks Hypothesis:Quantifying the Relations Between Digital-Screen Use and the Mental Well-Being of Adolescents. *Psychological Science* 2017;28(2):204-15. doi: 10.1177/0956797616678438

Confidential: For Review Only

16. Hamer M, Yates T, Sherar LB, et al. Association of after school sedentary behaviour in adolescence with mental wellbeing in adulthood. *Preventive Medicine* 2016;87(Supplement C):6-10. doi: https://doi.org/10.1016/j.ypmed.2016.02.021
17. Trainor S, Delfabbro P, Anderson S, et al. Leisure activities and adolescent psychological well-being. *Journal of Adolescence* 2010;33(1):173-86. doi: https://doi.org/10.1016/j.adolescence.2009.03.013
18. Landstedt E, Persson S. Bullying, cyberbullying, and mental health in young people. *Scandinavian journal of public health* 2014;42(4):393-9. doi: 10.1177/1403494814525004 [published Online First: 2014/03/13]
19. Przybylski AK, Bowes L. Cyberbullying and adolescent well-being in England: a population-based cross-sectional study. *The Lancet Child & Adolescent Health* 2017;1(1):19-26. doi: 10.1016/S2352-4642(17)30011-1

Confidential: For Review Only

BMJ Paediatrics Open

Confidential: For Review Only

The Impact of Health Behaviours and Deprivation on Wellbeing in a National Sample of English Young People

Journal:	*BMJ Paediatrics Open*
Manuscript ID	bmjpo-2018-000335.R2
Article Type:	Original article
Date Submitted by the Author:	06-Aug-2018
Complete List of Authors:	Gireesh, Aswathikutty; University College London, Institute of Child Health<br>Das, Shikta; University College London, Institute of Cardiovascular Science, MRC Unit for Lifelong Health and Ageing<br>Viner, Russell; UCL Institute of Child Health, Population, Policy & Practice Research Programme
Keywords:	Adolescent Health, Sleep, Nutrition

SCHOLARONE™
Manuscripts

TITLE

The Impact of Health Behaviours and Deprivation on Wellbeing in a National Sample of English Young People

Aswathikutty Gireesh MSc. [a*]

Shikta Das PhD [a*]

Prof. Russell Viner M.B.B.S., PhD [b]

*Joint first authors

[a] UCL Great Ormond Street Institute of Child Health, Population, Policy and Practice Programme, 30 Guilford Street, London WC1N 1EH, United Kingdom

[b] UCL Institute of Cardiovascular Science, MRC Unit for Lifelong Health and Ageing at UCL

Address for correspondence:

Aswathikutty Gireesh

Great Ormond Street Institute of Child Health, Population, Policy and Practice Programme.

30, Guilford Street, London WC1N 1EH, UK.

Email address: a.aswathikutty-gireesh@ucl.ac.uk

Word count: 2574

Confidential: For Review Only

ABSTRACT

Objective: To determine the modifiable factors influencing wellbeing in boys and girls by accounting for deprivation, ethnicity and clustering within local authorities.

Methods: We used data from a very large nationally-representative survey, the What About Youth (WAY) study involving 120,115 adolescents aged 15 years. Our outcome measure of mental wellbeing was the Warwick-Edinburgh Mental Wellbeing Scale (WEMWBS). Potential explanatory factors included substance abuse, screen time, eating habits, reading, bullying, sleeping pattern, physical activity and area-level deprivation. We ran unadjusted and adjusted multilevel models for each explanatory factor, after adjusting for ethnicity, deprivation and including a random effect for the local authority.

Results: Boys had a higher overall mean WEMWBS score than girls (p <0.0001). In the adjusted model, each of multiple risk behaviours, eating habits, sleep, bullying, physical activity, screen-time and reading were independently associated with mental wellbeing in both boy and girls (P<0.0001 for both). Sleep and eating behaviours had a stronger effect in both sexes than bullying, physical activity and screen time.

Conclusion: The largest contributors to adolescent wellbeing appear to be sleep, eating behaviours and bullying when considered in a multivariable framework. Future longitudinal studies and health policies need to consider a range of behavioural factors to drive improve improvements in adolescent wellbeing.

Confidential: For Review Only

INTRODUCTION

There are growing concerns about the wellbeing of young people in modern societies particularly in the UK where there is evidence that young people's wellbeing is lower than in many comparable developed countries [1 2].

Wellbeing is defined as "the state of being comfortable, healthy or happy" [3]. From a holistic perspective, wellbeing incorporates different dimensions of adolescent lives including social relationships as well as individual functioning [4]. However, the determinants of adolescent wellbeing is a relatively understudied area in comparison with the large literature on factors associated with mental health problems, as wellbeing concept has only been on greater focus over the past two decades [5 6].

A wide range of factors have been shown to be related to adolescent wellbeing, including a range cognitive and relational factors such as bullying [7], family structure and relationships [8], peer support [9] and school connectedness [10]. Other behaviours also influence wellbeing, including substance use (alcohol, drugs and smoking habits) [11-13], fruit and vegetable consumption [14], breakfast consumption [15], physical activity [16], sleep duration [17], sedentary behaviour [7 18] and leisure time activities [19]. However, published studies use a wide range of wellbeing measures, resulting in conflicting findings [20 21]. Furthermore, studies have largely focused on single risk factors and not explored how factors including behavioural factors interact to influence wellbeing. Additionally, given that many such behaviours are strongly socially patterned, studies have thus far paid little attention to confounding by socioeconomic position and issues relating to the clustering of behaviours and wellbeing within localities.

Policy initiatives to improve wellbeing amongst young people have largely focussed upon cognitive and psychological factors related to resilience to adversity, and have paid little attention to the contribution of non-psychological modifiable factors, such as other lifestyle behaviours. Understanding the potential contribution of modifiable behavioural factors to adolescent wellbeing may inform different strategies to improve young people's wellbeing.

Confidential: For Review Only

We used a very large recent nationally-representative and population-based survey of English 15 year-olds to examine the contribution of individual-level modifiable behaviours to wellbeing, including potentially protective (sleep, reading, and physical activity) and risk behaviours (substance use, unhealthy eating habits and excessive screen time). Our objective was to identify modifiable behavioural factors for mental wellbeing in boys and girls using an adolescent-specific measure and accounting for deprivation, ethnicity and clustering within local authorities (LAs).

Confidential: For Review Only

METHODS

Study design and sample

The What About Youth study (WAY) is a large-scale youth-oriented survey funded by the Department of Health (DH) in England and carried out by NHS Digital in 2014 [22]. The primary aim of the survey was to collect robust local authority level data on youth health behaviours and general health to improve their health outcomes. Study participants were those who turned 15 years old in the academic year 2013/14. A random sampling methodology was employed to draw 298,080 participants from the National Pupil Database. The sample size was calculated to attain 1000 young people in each of 152 LAs in England; 2 LAs were merged with their nearest neighbours due to small size.

The achieved sample was 120,115 individuals, of whom 16% responded online and 84% via postal means (2,835 opted out). The response rates differed by gender, with adjusted response rates of 35% in boys and 49% in girls, and by deprivation, ethnicity and LA. Non-response weights using these factors were calculated to provide alignment between the achieved and target samples [22]. We obtained a fully anonymised cohort data electronically from the United Kingdom (UK) Data Service website[22]. The National Children's Bureau (NBC) carried out a detailed ethical review of the original study, but it was not required for this secondary data analysis.

Outcome variable

Mental wellbeing was measured using the Warwick-Edinburgh Mental Wellbeing Scale (WEMWBS) [23], a population-level wellbeing measure. It is validated to use in adolescents' aged 13 or more and focus primarily on positive aspects of mental wellbeing (Internal consistency, α=0.90). Participants indicate how often they feel like each of the 14 items using a 5-point scale that ranges from 5 "all the time" to 1 "none of the time" [24]. Total scores ranged from 14-70 and were calculated by summing each participant's responses. The potential explanatory behavioural variables were identified from the literature review of previous publication. A detail description of each variable is given in Appendix A.

A composite variable for risk behaviour index for substance use was constructed by the summation of three dichotomous risk behaviour variables: (a) Smoking: If currently smokes (b) Drinking alcohol: If drinks once a month or more frequently (c) Cannabis use: If ever tried cannabis. Based on the number of risk behaviours, we categorised it from "none" to "three". Similarly, composite unhealthy eating habit index was derived from (a) Skipping Breakfast: If avoided breakfast in last 7 days (b) Poor diet: If consumed less than 5 portions of fruits and vegetables a day (c) Takeaway food: If consumed takeaway food in past 7 days. Based on a combination of unhealthy behaviours, the composite score was categorised into 0= none, 1= only one, 2 =any two and 3 =all three. Physical active for 60+ minutes for at least 5 days were classified as "physically active" and the rest "physically inactive." This threshold was defined in line with government recommendations [25 26], except for the intensity of exercise which was not available in the dataset. The selected threshold was taken at five days a week, as only 13% reported being physically active for seven days a week. A digital screen time variable was computed based on reported weekend and weekday usage of television, internet, smartphone and computer games. Subjects were categorised into "≥7hrs/day", "About 5-6 hours/day", "About 3 -4 hours/day", "About 2 hrs/day" and "≤1hrs/day". Time spent reading on weekends and weekdays had response options ranging

Confidential: For Review Only

from none to seven hours per day. Based on the distribution of data, we recoded the variable as "none", about half an hour/day", "about 1 hour/day" and "2 hours /day". The frequency of eight hours sleep in the last seven days was coded as "every day", "most days", "some days" and "not in the past 7 days". Bullying was measured with Olweus Bully/Victim Questionnaire, a reliable 8-item scale used to assess the bullying victimisation [27]. We combined responses to create one overall measure of bullying experience (yes/no). In line with a previous study [28], adolescents who were bullied more than "two or three times a month" were categorised as bullying victims.

Ethnicity, deprivation and mode of questionnaire completion were selected as confounders in the relationship between wellbeing and potential explanatory variables as shown in previous studies [7]. Ethnicity was self-identified by participants' and was an adaptation of the 2001 UK census categories, supplemented by questions on the national group. English Index of Multiple Deprivation (IMD) was used as a measure of relative deprivation for small areas [29]. IMD scores were divided into three deprivation categories as defined by quintiles of the national distribution: 1 and 2 (high deprivation), 3 (average), 4 and 5 (low). Participants were allowed to choose between online or postal modes of questionnaire completion.

Analyses

We conducted unadjusted and adjusted multilevel regression, in Stata 14 (Stata Corp, College Station, TX). For wellbeing scores, the interaction between gender and health behaviours was statistically significant ($p<0.001$) and therefore, analyses were stratified by gender. All variables were plotted to check the distribution and normality was checked with the Kolmogorov-Smirnov (K-S) test. All estimates were weighted by representativeness of participants to compensate for the disproportionate selection of sample and non-response bias. Pearson $\chi 2$ tests were used to compare differences in the distribution of explanatory variables by gender. An unadjusted analysis was run to test the association between each

Confidential: For Review Only

independent variable (substance use, unhealthy eating habits, screen time, reading, bullying, physical activity and sleeping hours) and the outcome adolescent wellbeing (Model 1). The analyses were repeated in a multivariable analysis where ethnicity, mode of questionnaire delivery and IMD were added as confounders between each risk factor and outcome (Model 2). In model 3, all explanatory factors and covariates were included simultaneously to obtain associations between each variable and wellbeing scores after adjusting for confounders and other explanatory variables. LAs were treated as random effects in all models. Models were also tested for significant quadratic terms signifying curvilinear relationships; this was only significant for reading. The intraclass correlations coefficient (ICC), being the proportion of total variance attributable to differences at the LA level, was estimated using multilevel models for wellbeing with adjustment for IMD, ethnicity and mode of completion.

Results

In total, 57,153 boys (47.82%) and 62,962 girls (52.18%) participated in the study (Table A1). Of these, boys had higher average wellbeing score in comparison to girls. The intracluster correlation coefficient (ICC) for the wellbeing score was 0.032 for girls and 0.024 for boys in the adjusted model, suggesting that variance in adolescent wellbeing is small at LA level.

Table A1 shows that there were significant differences in the distribution of potential explanatory variables between boys and girls. Girls had higher risk factors such as substance use, unhealthy eating habits, screen time and reported more bullying than boys. Protective variables such as sleeping hours (more than 8 hours) and reading were also significantly higher in girls than in boys.

Table A1. Descriptive statistics for well-being scores and explanatory variables under study, by gender

	Total	Boys		Girls		
	N	N	Mean (SD) / %	N	Mean (SD) / %	P value

Confidential: For Review Only

	**120,115**	**57,153**	**47.58%**	**62,962**	**52.42%**	
**WEMWBS Scores** [a]	117,842	56,352	47.82%	61,490	52.18%	<0.0001
Mean (SD)			50 (8.60)		45 (9.66)	
**Substance use** [b]	71,133	32,516	45.71%	38617	54.29%	<0.0001
None		17,133	52.69	19932	51.61	
One		10,783	33.16	12150	31.46	
Two		3,010	9.26	3,785	9.8	
Three		1,590	4.89	2,750	7.12	
**Unhealthy Eating Habits** [c]	115918	55,289	47.70%	60,629	52.30%	<0.0001
None		12,081	21.85	11,951	19.71	
One		20,287	36.69	20,389	33.63	
Two		16,373	29.61	19,064	31.44	
Three		6,548	11.84	9,225	15.22	
**Sleeping hours (>8 hrs)**	117,516	56, 207	47.83%	61307	52.17%	<0.0001
Not in the past 7 days		3676	6.54	6922	11.29	
some days		11051	19.66	16051	26.18	
most days		20, 121	35.8	20928	34.14	
everyday		21, 361	38	17406	28.39	
**Bullying**	117,744	56,309	47.82%	61,435	52.18%	<0.0001
No		45,959	81.62	45,094	73.40	
yes		10,350	18.38	16,341	26.60	
**Physical activity**	118450	56,674	47.85	61776	52.15	<0.0001
Physically active		48,172	85	44,348	71.79	
Inactive		8,502	15	17,428	28.21	
**Screen time**	118,845	56,892	47.87	61,943	52.13%	<0.0001
About 2 hrs/day		3,609	6.34	3,469	5.6	
≤1hrs/day		912	1.6	929	1.5	
About 3 -4 hours/day		18,621	32.73	17,311	27.95	
About 5-6 hours/day		17,198	30.23	17,850	28.82	
≥7hrs/day		16,559	29.1	22387	36.14	
**Reading**	118,140	56,513	47.84	61,627	52.16	<0.0001
None		14,278	25.26	9,875	16.02	
About Half an hour/day		17,572	31.09	15,363	24.93	
About 1 hr/day		12,505	22.13	13,442	21.81	

[a] WEMWBS : Warwick-Edinburgh Mental Wellbeing score

[b] Risk behaviours include smoking, drinking and cannabis use

[c] Unhealthy eating habits include skipping breakfast, not having 5 portions of fruits and vegetables and consumption of takeaway food.

All explanatory variables and IMD were significantly associated with wellbeing in both sexes (Table A2). Wellbeing in both sexes decreased with use of substance use, unhealthy eating habits, bullying, physical activity and longer screen time in both sexes. Protective factors,

Confidential: For Review Only

such as, sleeping more than eight hours and reading more than two hours were associated with higher wellbeing in both sexes.

Table A2: Univariate analysis between well-being and explanatory variables, by gender

	MODEL [1]			
	Boys		Girls	
	B	**95 % CI**	***B***	**95 % CI**
**Substance Use**				
None	Reference		Reference	
One	-0.34*	[-0.56,-0.13]	-1.48**	[-1.70,-1.27]
Two	-2.19**	[-2.60,-1.78]	-4.96**	[-5.35,-4.57]
Three	-3.56**	[-4.16,-2.95]	-5.89**	[-6.47,-5.30]
**Unhealthy Eating Habits**				
None	Reference		Reference	
One	-1.65**	[-1.88,-1.43]	-2.63**	[-2.91,-2.34]
Two	-3.36**	[-3.60,-3.12]	-4.85**	[-5.13,-4.58]
Three	-5.00**	[-5.39,-4.61]	-6.49**	[-6.83,-6.14]
**Sleeping hours (>8 hrs)**				
Not in the past 7 days	Reference		Reference	
some days	2.99**	[2.51,3.47]	4.71**	[4.39,5.04]
most days	5.59**	[5.15,6.03]	8.39**	[8.06,8.72]
everyday	7.49**	[7.07,7.87]	10.70**	[10.35,11.06]
**Bullying**				
No	Reference		Reference	
Yes	-4.70**	[-3.59,-3.31]	-5.84**	[-5.25,-4.90]
**Physical activity**				
Physically active	Reference		Reference	
Inactive	-3.85**	[-4.11,-3.61]	-2.77**	[-3.04,-2.51]
**Screen time**				
About 2 hrs/day	Reference		Reference	
≤1hrs/day	0.07	[-0.59, 0.73]	0.08	[-0.69, 0.85]
About 3 -4 hours/day	-1.22**	[-1.60,-0.84]	-1.27**	[-1.71,-0.83]
About 5-6 hours/day	-2.17**	[-2.51,-1.83]	-3.06**	[-3.52,-2.60]
>= 7 hours / day	-3.72**	[-4.12,-3.32]	-5.38**	[-5.80,-4.97]
**Reading**				
None	Reference		Reference	
About Half an hour/day	1.61**	[1.41,1.82]	2.17**	[1.88,2.46]
About 1 hr/day	2.25**	[2.06,2.44]	2.54**	[2.26,2.82]
≥2hrs/day	2.27***	[2.01,2.52]	2.35**	[2.05,2.66]
**IMD Scores**				
High deprivation	Reference		Reference	
Average deprivation	0.57**	[0.31,0.81]	0.35**	[0.10,0.59]

Confidential: For Review Only

Least deprivation	1.05**	[0.87,1.21]	1.16**	[0.92,1.40]
**Ethnicity**				
White	Reference		Reference	
Mixed	0.18	[-0.29,0.65]	-0.44*	[-0.82,-0.06]
Asian	-0.54*	[-0.85,-0.23]	0.98**	[0.59,1.38]
Black	0.91**	[0.42,1.39]	1.10**	[0.67,1.52]
Other	-0.45*	[-0.84,-0.04]	0.30	[-0.09,0.70]

[1]Unadjusted model taking into account clustering at Local Authority Level, Multilevel models fitted with weighted design weights, Quadratic function added to reading.

** p<0.001, * p<0.05

In the multivariable models adjusted for covariates (Table A3), poorer wellbeing was associated with multiple substances use and multiple unhealthy eating habits in a dose-dependent fashion. Being physically inactive, longer screen time and experiencing bullying were both associated with decrements in wellbeing in both sexes, with the association being stronger in girls than in boys.

Confidential: For Review Only

Table A3: Gender - stratified partially-adjusted and fully-adjusted multilevel modelling for well-being and explanatory variables

	Boys				Girls			
	MODEL[2]		MODEL [3]		MODEL [2]		MODEL [3]	
**Variables**	B	95 % CI	B	95 % CI	B	95 % CI	B	95 % CI
**Substance use**								
None	Reference		Reference		Reference		Reference	
One	-0.40**	[-0.61,-0.18]	-0.14	[-0.36,0.08]	-1.51**	[-1.73,-1.30]	-0.77**	[-0.97,-0.57]
Two	-2.16**	[-2.57,-1.75]	-1.05**	[-1.42,-0.67]	-4.84**	[-5.22,-4.46]	-2.67**	[-3.01,-2.33]
Three	-3.50**	[-4.11,-2.88]	-1.63**	[-2.16,-1.09]	-5.80**	[-6.36,-5.23]	-2.79**	[-3.35,-2.24]
**Eating Habits**								
None	Reference		Reference		Reference		Reference	
One	-1.63**	[-1.85,-1.41]	-0.89**	[-1.18,-0.60]	-2.63**	[-2.92,-2.34]	-1.37**	[-1.69,-1.06]
Two	-3.31**	[-3.55,-3.08]	-1.84**	[-2.14,-1.54]	-4.84**	[-5.12,-4.55]	-2.29**	[-2.64,-1.96]
Three	-4.95**	[-5.34,-4.56]	-2.44**	[-2.82,-2.06]	-6.46**	[-6.79,-6.10]	-2.61**	[-3.04,-2.18]
**Sleeping hours (>8 hrs)**								
Not in the past 7 days	Reference		Reference		Reference		Reference	
some days	2.98**	[2.50,3.45]	2.69**	[2.10,3.28]	4.69**	[4.36,5.01]	3.71**	[3.34,4.08]
most days	5.55**	[5.11,5.99]	4.30**	[3.78,4.82]	8.35**	[8.01,8.69]	6.64**	[6.28,7.00]
everyday	7.45**	[7.03,7.86]	5.79**	[5.29,6.28]	10.65**	[10.29,11.01]	8.16**	[7.72,8.60]
**Bullying**								
No	Reference		Reference		Reference		Reference	
Yes	-4.64**	[-4.86,-4.42]	-3.78**	[-4.09,-3.48]	-5.77**	[-5.98,-5.56]	-4.01**	[-4.23,-3.78]
**Physical activity**								
Physically active	Reference		Reference		Reference		Reference	
Inactive	-3.78**	[-4.02,-3.55]	-2.63**	[-2.95,-2.30]	-2.77**	[-3.04, -2.50]	-1.70**	[-2.01,-1.39]
**Screen time**								
About 2 hrs/day	Reference		Reference		Reference		Reference	
≤1hrs/day	0.09	[-0.57,0.75]	0.34	[-0.58,1.26]	0.09	[-0.69,0.87]	-0.38	[-1.49,0.72]
About 3 -4 hours/day	-1.22**	[-1.60,-0.83]	-0.61*	[-0.99,-0.23]	-1.26**	[-1.70,-0.83]	-0.54	[-1.14,0.05]
About 5-6 hours/day	-2.15**	[-2.50,-1.81]	-0.82**	[-1.27,-0.37]	-3.04**	[-3.49,-2.58]	-1.21**	[-1.75,-0.67]
>= 7 hours / day	-3.67**	[-4.06,-3.27]	-1.20**	[-1.65,-0.75]	-5.32**	[-5.73,-4.90]	-1.81**	[-2.29,-1.33]

**Reading**								
None	Reference		Reference		Reference		Reference	
About Half an hour/day	1.59**	[1.38,1.80]	0.57**	[0.26,0.88]	2.13**	[1.84,2.42]	0.56**	[0.23,0.90]
About 1 hr/day	2.24**	[2.04,2.43]	1.04**	[0.79,1.29]	2.51**	[2.24,2.78]	0.60**	[0.26,0.93]
≥2hrs/day	2.28**	[2.05,2.54]	0.90**	[0.55,1.25]	2.34**	[2.03,2.64]	0.33*	[0.02,0.64]
**IMD Scores**								
High deprivation	--	--	Reference		--	--	Reference	
Average deprivation	--	--	-0.12	[-0.38,0.14]	--	--	0.16	[-0.16,0.49]
Least deprivation	--	--	0.17	[-0.06,0.40]	--	--	0.36*	[0.06,0.66]
**Ethnicity**								
White	--	--	Reference		--	--	Reference	
Mixed	--	--	-0.06	[0.81,-0.59]	--	--	0.44	[-0.08,0.96]
Asian	--	--	-0.09	[0.81,-0.84]	--	--	0.35	[-0.53,1.23]
Black	--	--	0.99*	[0.01,0.25]	--	--	1.75**	[1.20,2.31]
Other	--	--	-0.59	[0.02,-1.09]	--	--	-0.19	[-0.88,0.49]
**Mode of questionnaire delivery**								
Online	--	--			--	--		
paper	--	--	0.24	[-0.03,0.51]	--	--	1.26**	[0.99,1.53]

[2] Each predictor variable adjusted for ethnicity, mode of questionnaire delivery and Index of multiple deprivation

[3] All Variables mutually adjusted for each other

Note: Multilevel models fitted with weighted design weights, Quadratic function added to reading.

** p<0.001, * p<0.05

Confidential: For Review Only

Higher wellbeing was associated with number of days young people achieved more than eight hours of sleep again in a dose-dependent fashion. Habitual reading most days was associated with higher wellbeing although there was no evidence of a dose-response above 30 minutes per day in the fully adjusted model (Figure 1). Adolescents from black ethnic groups had higher wellbeing scores overall. Area deprivation did not affect male wellbeing but had a small effect on female wellbeing.

Confidential: For Review Only

Discussion

This study broadens our understanding of risk and protective factors associated with wellbeing in adolescence, using a very large nationally representative survey to examine a wide spectrum of behavioural and psychosocial factors relating to youth wellbeing and taking into account deprivation and clustering at LA level. The study shows that young people who reported lower levels of wellbeing were more likely to have engaged in multiple unhealthy eating habits and substance use; be victims of bullying; have exercised insufficiently; have exceeded recommended screen time use. These findings were robust to mutual adjustment for all variables and for deprivation, ethnicity, and mode of questionnaire delivery. A dose-response pattern was also observed between wellbeing and health behaviours such as substance use, unhealthy eating habits and sleeping pattern. A decrease in the number of risk behaviours and an increase in the number of days slept for more than eight hours, corresponded with an increase in average levels of wellbeing. The impact of deprivation on wellbeing was surprisingly small, as was LA locality variance, suggesting that variance in wellbeing lies largely in behavioural and psychological factors.

Since different studies have used different variables to define various aspects of wellbeing in the analyses, comparison with other studies is difficult. We found boys reported higher mean wellbeing than girls, consistent with national reports for England [30 31], but in contrast to findings from recent Health Survey England (HSE) 2015, where only slight gender variations were observed [32]. The proportions reporting each of the behaviours were broadly similar to those found in other recent national surveys [31 33 34]. Girls reported higher levels of risky

health behaviours including current smoking, alcohol consumption, bullying, lower levels of physical activity, on the other hand, boys were more likely to report higher levels of physical activity and these were consistent with findings from the HBSC England [33].

These findings corroborate with two previous studies where happiness as a marker of wellbeing was found to be positively associated with multiple health protective behaviours (sport's participation and healthier eating) and negatively associated with multiple risk behaviours (smoking, alcohol use and heavy screen use) in adolescents [35 36]. Our finding that substance use was associated with lower wellbeing is similar to that seen in other studies, as were our findings for being bullied [7]. The association of sleep duration and reading with wellbeing in young people has been little studied. Leisure time and adequate sleep have been identified as being associated with wellbeing[17 19], however ours is the first to examine these alongside other behavioural and psychological factors.

We found an association between deprivation and lower wellbeing, although in contrast to previous studies[37] the effect size was small and we found no association in boys. This may reflect the lack of adjustment for multiple behaviours, ethnicity and area effects in other studies. Our finding suggests that much of what has previously been understood, as unhealthy behaviours themselves associated with deprivation may mediate deprivation effects. We found that young people from black ethnic groups reported significantly higher wellbeing in both sexes, consistent with previous UK findings [38]. However, the reasons for this remain unclear and require further study. We found this association to be robust to adjustment for deprivation and all significant behavioural and psychological factors, suggesting this likely relates to factors not measured in our study.

We used a large, nationally representative sample of ethnically and socioeconomically diverse adolescents. Prior studies have examined very few health behaviours and relied on proxy measures of wellbeing rather than on population-level wellbeing measures that tap into

Confidential: For Review Only

both feelings and psychological flourishing. In our study, associations between wellbeing and behavioural factors were examined within a multivariable and multilevel framework, using a validated wellbeing scale with robust psychometric properties.

Our findings are subject to a number of limitations. Our data were cross-sectional and thus the direction of causality is unclear for the behavioural variables. Participant responses could be influenced by social desirability, and those with poor wellbeing may be inclined towards endorsing questions more than others, thus introducing bias. The direction of such biases is unclear, however, we note that girls (who had a higher response rate in the overall survey) reported higher levels of both more risky and protective behaviours than boys, potentially reflecting social desirability biases. All variables used were self-reported except for area-level deprivation. We have also repeated analysis excluding the outliers and that did not materially affect the findings. We used bullying victimization as a proxy for psychological problems due to the lack of more appropriate variables in the dataset; thus it is possible that some of the associations seen here result from inadequate adjustment for psychological issues. We combined variables across domains into composite variables; this may have introduced bias although the direction of bias is unclear.

Conclusion

Our findings suggest that promoting healthy sleep, reading, and healthy eating behaviours may present important policy targets for enhancing adolescent wellbeing in addition to more accepted foci on physical activity, screen time and bullying. While there was an association between deprivation and wellbeing, the effect size was small. Future work is needed to examine these modifiable factors within a longitudinal causal framework.

Confidential: For Review Only

What is already known on this topic?

Young people’s wellbeing may be affected by multiple individual and contextual factors.

Key determinants of adolescent wellbeing remain unclear.

Few studies have examined a wide range of potential determinants whilst adjusting for area-level deprivation.

What this study adds?

Findings support current policy foci on bullying, physical activity and screen-time as correlates of wellbeing amongst young people.

Sleep and eating behaviours may also be important policy targets for promoting adolescent wellbeing.

A coherent policy framework to promote adolescent wellbeing needs to be multifaceted and consider a range of health factors in young people's lives

Confidential: For Review Only

Funding Sources

This report is independent research commissioned and funded by the National Institute for Health Research Policy Research Programme. The views expressed in this publication are those of the author(s) and not necessarily those of the NHS, the National Institute for Health Research, the Department of Health or its arm's length bodies, and other Government Departments.

Conflicts of interests

The authors have no conflicts of interest to disclose

Contributors

AG & SD led the writing of the study and undertook the analyses. RV conceptualised the project, contributed to the ideas underlying the article, contributed to writing and revision of the article.

Acknowledgement

Preliminary findings of this paper were presented at the Adolescent Lives and Wellbeing 2017 Conference organised by University College London.

Confidential: For Review Only

References

1. Patton GC, Sawyer SM, Santelli JS, et al. Our future: a Lancet commission on adolescent health and wellbeing. *Lancet (London, England)* 2016;387(10036):2423-78. doi: 10.1016/s0140-6736(16)00579-1 [published Online First: 2016/05/14]

2. RCPCH. State of Child Health Report Royal College of Paediatrics and Child Health; 2017 [Available from: http://www.thehealthwell.info/node/1062390 accessed 6th November 2017.

3. Oxford University Press. Oxford Dictionary. Oxford: Oxford University Press 2016.

4. Žukauskienė R. Adolescence and Well-Being. In: Ben-Arieh A, Casas F, Frønes I, et al., eds. Handbook of Child Well-Being: Theories, Methods and Policies in Global Perspective. Dordrecht: Springer Netherlands 2014:1713-38.

5. Das JK, Salam RA, Lassi ZS, et al. Interventions for Adolescent Mental Health: An Overview of Systematic Reviews. *Journal of Adolescent Health* 2016;59(4, Supplement):S49-S60. doi: https://doi.org/10.1016/j.jadohealth.2016.06.020

6. Keyes CL. Promoting and protecting mental health as flourishing: a complementary strategy for improving national mental health. *The American psychologist* 2007;62(2):95-108. doi: 10.1037/0003-066x.62.2.95 [published Online First: 2007/02/28]

7. Przybylski AK, Bowes L. Cyberbullying and adolescent well-being in England: a population-based cross-sectional study. *The Lancet Child & Adolescent Health* 2017;1(1):19-26. doi: 10.1016/S2352-4642(17)30011-1

8. Dinisman T, Andresen S, Montserrat C, et al. Family structure and family relationship from the child well-being perspective: Findings from comparative analysis. *Children and Youth Services Review* 2017;80(Supplement C):105-15. doi: https://doi.org/10.1016/j.childyouth.2017.06.064

9. Raja SN, McGee R, Stanton WR. Perceived attachments to parents and peers and psychological well-being in adolescence. *Journal of Youth and Adolescence* 1992;21(4):471-85. doi: 10.1007/bf01537898

10. García Bacete FJ, Marande Perrin G, Schneider BH, et al. Effects of School on the Well-Being of Children and Adolescents. In: Ben-Arieh A, Casas F, Frønes I, et al., eds. Handbook of Child Well-Being:

Confidential: For Review Only

Theories, Methods and Policies in Global Perspective. Dordrecht: Springer Netherlands 2014:1251-305.

11. Taylor G, McNeill A, Girling A, et al. Change in mental health after smoking cessation: systematic review and meta-analysis. *BMJ : British Medical Journal* 2014;348 doi: 10.1136/bmj.g1151

12. Farmer S, Hanratty B. The relationship between subjective wellbeing, low income and substance use among schoolchildren in the north west of England: a cross-sectional study. *Journal of Public Health* 2012;34(4):512-22. doi: 10.1093/pubmed/fds022

13. Phillips-Howard PA, Bellis MA, Briant LB, et al. Wellbeing, alcohol use and sexual activity in young teenagers: findings from a cross-sectional survey in school children in North West England. *Substance abuse treatment, prevention, and policy* 2010;5:27. doi: 10.1186/1747-597x-5-27 [published Online First: 2010/11/12]

14. Conner TS, Brookie KL, Carr AC, et al. Let them eat fruit! The effect of fruit and vegetable consumption on psychological well-being in young adults: A randomized controlled trial. *PLoS ONE* 2017;12(2):e0171206. doi: 10.1371/journal.pone.0171206

15. Lien L. Is breakfast consumption related to mental distress and academic performance in adolescents? *Public Health Nutrition* 2007;10(4):422-28. doi: 10.1017/S1368980007258550 [published Online First: 04/01]

16. Ussher MH, Owen CG, Cook DG, et al. The relationship between physical activity, sedentary behaviour and psychological wellbeing among adolescents. *Social Psychiatry and Psychiatric Epidemiology* 2007;42(10):851-56. doi: 10.1007/s00127-007-0232-x

17. Fuligni AJ, Hardway C. Daily Variation in Adolescents' Sleep, Activities, and Psychological Well-Being. *Journal of Research on Adolescence* 2006;16(3):353-78. doi: 10.1111/j.1532-7795.2006.00498.x

18. Hamer M, Yates T, Sherar LB, et al. Association of after school sedentary behaviour in adolescence with mental wellbeing in adulthood. *Preventive Medicine* 2016;87(Supplement C):6-10. doi: https://doi.org/10.1016/j.ypmed.2016.02.021

19. Trainor S, Delfabbro P, Anderson S, et al. Leisure activities and adolescent psychological well-being. *Journal of Adolescence* 2010;33(1):173-86. doi: https://doi.org/10.1016/j.adolescence.2009.03.013

Confidential: For Review Only

20. Pollard EL, Lee PD. Child Well-being: A Systematic Review of the Literature. *Social Indicators Research* 2003;61(1):59-78. doi: 10.1023/a:1021284215801

21. Rose T, Joe S, Williams A, et al. Measuring Mental Wellbeing Among Adolescents: A Systematic Review of Instruments. *Journal of Child and Family Studies* 2017;26(9):2349-62. doi: 10.1007/s10826-017-0754-0

22. Health and Social Care Information Centre. What About YOUth? Survey. England: UK Data Service, 2016.

23. Tennant R, Hiller L, Fishwick R, et al. The Warwick-Edinburgh Mental Well-being Scale (WEMWBS): development and UK validation. *Health and Quality of Life Outcomes* 2007;5:63-63. doi: 10.1186/1477-7525-5-63

24. Taggart F, Stewart-Brown S, Parkinson J. Warwick-Edinburgh Mental Well-being Scale (WEMWBS) Scotland: NHS Health Scotland; 2015 [Available from: http://www.healthscotland.com/uploads/documents/26787-WEMWBS%20User%20Guide%20Version%202%20May%202015.pdf accessed 19th October 2017.

25. Morgan K, Hallingberg B, Littlecott H, et al. Predictors of physical activity and sedentary behaviours among 11-16 year olds: Multilevel analysis of the 2013 Health Behaviour in School-aged Children (HBSC) study in Wales. *BMC Public Health* 2016;16:569. doi: 10.1186/s12889-016-3213-8

26. Booth ML, Okely AD, Chey T, et al. The reliability and validity of the physical activity questions in the WHO health behaviour in schoolchildren (HBSC) survey: a population study. *British journal of sports medicine* 2001;35(4):263-7. [published Online First: 2001/07/31]

27. Olweus D. The Revised Olweus Bully/Victim Questionnaire. University of Bergen: Research Center for Health Promotion (HEMIL Center) 1996.

28. Cook CR, Williams KR, Guerra NG, et al. Predictors of bullying and victimization in childhood and adolescence: A meta-analytic investigation. *School Psychology Quarterly* 2010;25(2):65-83. doi: 10.1037/a0020149

29. Department for Communities and Local Government. The English indices of deprivation London2015 [Available from:

Confidential: For Review Only

https://www.gov.uk/government/uploads/system/uploads/attachment_data/file/465791/English_Indices_of_Deprivation_2015_-_Statistical_Release.pdf

30. Office of National Statistics. Measuring National Well-being: Insights into children's mental health and well-being. England, 2015.

31. Office of National Statistics. Young people's well-being England: Office for National Statistics; 2017 [Available from: https://www.ons.gov.uk/peoplepopulationandcommunity/wellbeing/articles/youngpeopleswellbeingandpersonalfinance/2017 accessed 10th November 2017.

32. Fuller E, Morris S. Health Survey for England. London NHS Digital, 2015.

33. Currie C, Grieber R, Inchley J. Health Behaviour in School-Aged Children (HBSC) Study Protocol: Background, Methodology and Mandatory Items for the 2009/10 Survey. Edinburgh: CAHRU 2010.

34. Brooks F, Magnusson J, Klemera E, et al. Health Behaviours of School-Aged Children (HBSC) 2013-2014 England2014 [Available from: www.hbscengland.com.

35. Booker CL, Skew AJ, Sacker A, et al. Well-Being in Adolescence—An Association With Health-Related Behaviors:Findings From Understanding Society, the UK Household Longitudinal Study. *The Journal of Early Adolescence* 2014;34(4):518-38. doi: 10.1177/0272431613501082

36. Booker CL, Skew AJ, Kelly YJ, et al. Media Use, Sports Participation, and Well-Being in Adolescence: Cross-Sectional Findings From the UK Household Longitudinal Study. *American Journal of Public Health* 2015;105(1):173-79. doi: 10.2105/AJPH.2013.301783

37. Candace Currie, Cara Zanotti, Antony Morgan, et al. Social determinants of health and well-being among young people. International report from the 2009/2010 survey. Health Behavior in school-age children (HBSC) study. Denmark: WHO Regional Office for Europe, 2012:252.

38. Patalay P, Fitzsimons E. Correlates of Mental Illness and Wellbeing in Children: Are They the Same? Results From the UK Millennium Cohort Study. *Journal of the American Academy of Child & Adolescent Psychiatry* 2016;55(9):771-83. doi: 10.1016/j.jaac.2016.05.019

.

Confidential: For Review Only

Confidential: For Review Only

Figure 1

Relationship between wellbeing and health behaviours: (A) Risk Factors (B) Protective Factors

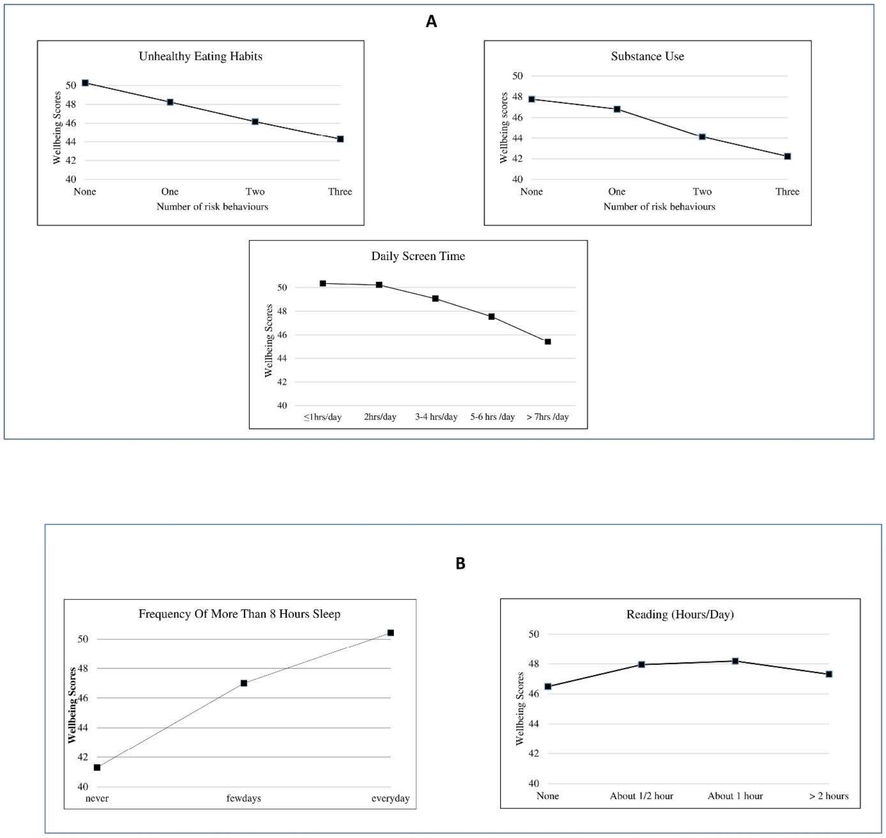

APPENDIX A

(i) VARIABLE DESCRIPTION

Composite variables	Original variables	Variable labels	Composite variables
**Substance use risk [1-6]**	**Definitions were:**		**Alcohol + smoking + cannabis**
	Smoking: Cigarette smoking status (currently smoke)	SmkSt2	(1) Smoking risk: If currently smokes (2) Drinking risk: If drinks once a month or more frequently (3) Cannabis risk: If ever tried cannabis. Each item was coded 0 for "no" and 1 for "yes". The composite score obtained ranged from zero to three based on the number of risk behaviours; (0= None, 1= Only one, 2 =Any two and 3 =All three
	Drinking: Alcohol drinking status (Usually have an alcoholic drink once a month or more frequently)	alcfrq1	
	Cannabis: Cannabis use status (Ever tried cannabis)	Cantry	
**Physical Activity [7 8]**	**Physical activity: in line with general recommendations**		**Duration of exercise + Frequency of exercise + Number of days met recommended guidelines**
	How many days physically active for 60+ minutes, in last 7 days (Active for more than 5 days)	Physact	The first question on moderate to vigorous physical activity was "Over the past 7 days, on how many days you were physically active for a total of at least 60 minutes per day and responses ranged from 0 to 7. Second, the participants were asked "Outside school hours: how often do you usually exercise in your free time so much that you get out of breath or sweat?" with responses from less than a once a month to every day. The question on duration was "Outside school hours: how many hours do you usually exercise in your free time so much that you get out of breath or sweat?", with responses from none to 7 or more hours. Adolescents who exercised for more than twice a week for an hour or more or were physically active for 60+ minutes for at least 5 days were classified as "physically active" and the rest "physically inactive".
	How often usually exercise in free time (Active for more than twice a week)	ExerOft	
	How many hours usually exercise in free (Active for an hour or more)	ExerHrs	
**Sleeping habits [9 10]**	**Sleeping frequency in last week**		
	How often slept 8+ hours per night in last 7 days	Sleep8pn	The question was "Over the past 7 days, how often did you sleep for 8 hours or more" and the responses were "every day", "most days", "some days" and "not in the past 7 days".

Confidential: For Review Only

**Eating Habits [11-13]**	**Definition of each unhealthy eating habits were:**		**Breakfast consumption + take away food + 5 A day**
	How often eaten take-away food in last 7 days (unhealthy habit if taken takeaway in past seven days)	Eattake	(1) Skipping Breakfast: If avoided breakfast in last 7 days (2) Poor diet: If consumed less than 5 portions of fruits and vegetables a day (3) Takeaway food: If consumed takeaway food in past 7 days. Each item was coded 0 for "no" and 1 for "yes". According to the number of unhealthy eating habits participant's exhibited, a risk score from zero to three (0= None, 1= Only one, 2 =Any two and 3 =All three) was given
	How often eaten breakfast in last 7 days (unhealthy habit if breakfast not taken regularly)	Eatbfst	
	Consumed more than five portions of fruit and veg yesterday (unhealthy habit if 5-A-DAY not taken regularly)	FV5ormor	
**Screen time [8 14-16]**	**Definitions of screen time derived as follows (Weekend and weekday hours combined)**		**TV +_computer games + internet**
	Hours per day watching TV	Watchwk, Watchwe	FOR ALL, Response options for each item were 'none at all', 'about half an hour a day', 'about 1 hour a day', 'about 2 hours a day', 'about 3 hours a day', 'about 4 hours a day', 'about 5 hours a day', 'about 6 hours a day' and 'about 7 or more hours a day'. The total screen time was calculated by grouping the similar options of each item and by combining both weekends and weekdays. Subjects were then categorised into "≥7hrs/day", "About 5-6 hours/day", "About 3 -4 hours/day", "About 2 hrs/day" and "≤1hrs/day".
	Hours per day spent playing computer games	Compwk , Compwe	
	Hours per day spent on computers for communicating, or homework	Comphwk, Comphwe	
	Hours per day spent on smart phone	Smartwk, Smartwe	
**Reading [17]**	**Definitions of reading derived as follows:**		**Reading hours (Weekend + weekdays)**
	Hours per day spent reading outside school hours	Readwk, Readwe	"About how many hours a day do you usually spend sitting down reading books, magazines or newspapers (including e-readers, online newspapers, and magazines) and studying when you are not at school?". The questions were asked for weekends and weekdays separately. The response options ranged from none to ≥ 7 hours per day
**Bullying [18 19]**	**Frequency with which the behaviour occurred over the past couple of months:**		**Traditional bullying (physical+ verbal + relational)**

Confidential: For Review Only

I was called mean names, was made fun of, or teased in a hurtful way	Bllynam	Bullying measure was meant to assess the extent to which they had recently experienced other children directing different types of bullying towards themselves. Six statements reflecting traditional bullying (physical, verbal, and relational) were asked. Questions were rated by participants using a 5-point response scale that ranged from 0="I haven't been bullied this way in the past couple of months" 1="It has happened once or twice", 2="2 or 3 times a month", 3="2 or 3 times a week", to 4="Several times a week."
Other people left me out of things on purpose, excluded me from their group of friends, or completely ignored me	Bllyout	
I was hit, kicked, pushed, shoved around, or locked indoors"	Bllyhit	
Other people lied or spread humours	Bllylie	
Made fun of me because of weight	Bllyfun	
Sexual jokes/comments	Bllysex	

(ii) Individual Items and scoring of Warwick-Edinburgh Mental Well-being scale (WEMWBS)

1. I've been feeling optimistic about the future
2. I've been feeling useful
3. I've been feeling relaxed
4. I've been feeling interested in other people
5. I've had energy to spare
6. I've been dealing with problems well
7. I've been thinking clearly
8. I've been feeling good about myself
9. I've been feeling close to other people
10. I've been feeling confident
11. I've been able to make up my own mind about things
12. I've been feeling loved
13. I've been interested in new things
14. I've been feeling cheerful

Each of the 14 item responses in WEMWBS are scored from 1 (none of the time), 2 (rarely), 3 (some of the time), 4 (often) to 5 (all of the time). A total scale score is calculated by summing the 14 individual item scores.

Confidential: For Review Only

REFERENCES

1. Farmer S, Hanratty B. The relationship between subjective wellbeing, low income and substance use among schoolchildren in the north west of England: a cross-sectional study. Journal of Public Health 2012;34(4):512-22. doi: 10.1093/pubmed/fds022
2. Phillips-Howard PA, Bellis MA, Briant LB, et al. Wellbeing, alcohol use and sexual activity in young teenagers: findings from a cross-sectional survey in school children in North West England. Substance abuse treatment, prevention, and policy 2010;5:27. doi: 10.1186/1747-597x-5-27 [published Online First: 2010/11/12]
3. Newcomb MD, Bentler PM, Collins C. Alcohol Use and Dissatisfaction with Self and Life: A Longitudinal Analysis of Young Adults. Journal of Drug Issues 1986;16(4):479-94. doi: 10.1177/002204268601600401
4. Bergman MM, Scott J. Young adolescents' wellbeing and health-risk behaviours: gender and socio-economic differences. Journal of Adolescence 2001;24(2):183-97. doi: https://doi.org/10.1006/jado.2001.0378
5. Taylor G, McNeill A, Girling A, et al. Change in mental health after smoking cessation: systematic review and meta-analysis. BMJ : British Medical Journal 2014;348 doi: 10.1136/bmj.g1151
6. Booker CL, Skew AJ, Sacker A, et al. Well-Being in Adolescence—An Association With Health-Related Behaviors:Findings From Understanding Society, the UK Household Longitudinal Study. The Journal of Early Adolescence 2014;34(4):518-38. doi: 10.1177/0272431613501082
7. Ussher MH, Owen CG, Cook DG, et al. The relationship between physical activity, sedentary behaviour and psychological wellbeing among adolescents. Social Psychiatry and Psychiatric Epidemiology 2007;42(10):851-56. doi: 10.1007/s00127-007-0232-x
8. Booker CL, Skew AJ, Kelly YJ, et al. Media Use, Sports Participation, and Well-Being in Adolescence: Cross-Sectional Findings From the UK Household Longitudinal Study. American Journal of Public Health 2015;105(1):173-9. doi: 10.2105/AJPH.2013.301783
9. Fuligni AJ, Hardway C. Daily Variation in Adolescents' Sleep, Activities, and Psychological Well-Being. Journal of Research on Adolescence 2006;16(3):353-78. doi: 10.1111/j.1532-7795.2006.00498.x
10. James S, Hale L. Sleep Duration and Child Wellbeing: A Nonlinear Association. Journal of clinical child and adolescent psychology : the official journal for the Society of Clinical Child and Adolescent Psychology, American Psychological Association, Division 53 2017;46(2):258-68. doi: 10.1080/15374416.2016.1204920
11. Rooney C, McKinley MC, Woodside JV. The potential role of fruit and vegetables in aspects of psychological well-being: a review of the literature and future directions. Proceedings of the Nutrition Society 2013;72(4):420-32. doi: 10.1017/S0029665113003388 [published Online First: 09/11]
12. Conner TS, Brookie KL, Carr AC, et al. Let them eat fruit! The effect of fruit and vegetable consumption on psychological well-being in young adults: A randomized controlled trial. PLoS ONE 2017;12(2):e0171206. doi: 10.1371/journal.pone.0171206
13. Lien L. Is breakfast consumption related to mental distress and academic performance in adolescents? Public Health Nutrition 2007;10(4):422-28. doi: 10.1017/S1368980007258550 [published Online First: 04/01]
14. Hoare E, Milton K, Foster C, et al. The associations between sedentary behaviour and mental health among adolescents: a systematic review. International Journal of Behavioral Nutrition and Physical Activity 2016;13(1):108. doi: 10.1186/s12966-016-0432-4
15. Przybylski AK, Weinstein N. A Large-Scale Test of the Goldilocks Hypothesis:Quantifying the Relations Between Digital-Screen Use and the Mental Well-Being of Adolescents. Psychological Science 2017;28(2):204-15. doi: 10.1177/0956797616678438

Confidential: For Review Only

16. Hamer M, Yates T, Sherar LB, et al. Association of after school sedentary behaviour in adolescence with mental wellbeing in adulthood. Preventive Medicine 2016;87(Supplement C):6-10. doi: https://doi.org/10.1016/j.ypmed.2016.02.021
17. Trainor S, Delfabbro P, Anderson S, et al. Leisure activities and adolescent psychological well-being. Journal of Adolescence 2010;33(1):173-86. doi: https://doi.org/10.1016/j.adolescence.2009.03.013
18. Landstedt E, Persson S. Bullying, cyberbullying, and mental health in young people. Scandinavian journal of public health 2014;42(4):393-9. doi: 10.1177/1403494814525004 [published Online First: 2014/03/13]
19. Przybylski AK, Bowes L. Cyberbullying and adolescent well-being in England: a population-based cross-sectional study. The Lancet Child & Adolescent Health 2017;1(1):19-26. doi: 10.1016/S2352-4642(17)30011-1

Confidential: For Review Only

Confidential: For Review Only

!	" #
$ % ! & &	' #
( ) * &	+ &, - & . !/ 0 1 ! (#) % , * (& % 2 &<br>, $& . / 0 1 ! (#) % , * (% 1<br>$, (0 *) * # 2 & % # #<br>3 , / 0() * (& % 2 &, 4 , 4 ! 5 4<br>& 4 #
6 !- %	% 2 &, $, 7

SCHOLARONE™
Manuscripts

! " # $ % & '

" '

& ( ) %& & & &*

'+

,-. $ / - * * #

* 01 $ * . -2 2 * , 3

,-. - * %(- , .

,-.

4

! " # $

$ / - * * # &

01* $ * . -2 2 * ,3&

4 & ! " #5 6 & & "

4 789:

Confidential: For Review Only

4 ! # #

* # ! &

#5 #*

; < # 271*228 28 # & /

! ! ! "5 % ; % <&

= # * * * *

* * # # 5 & >

> = # * > #*

#&

4 # % ; ?1&1112<&

> * " * * * # * #

#* 5 ! # ! !

; ?1&1112 <&

= # * # # & "

! = & ! !

! # ! &

! *

! ! "&

" ! *

! ! & @

! &

Confidential: For Review Only

! ! #

,3 ! # A ! !

2 7&

B * # #C 0& @

* !

! : & ! *

! # !

! * ! #

! 8 D&

! ! ! *

9* # E*

F 21& / ! *

; * " < 22520*

2: * " 28* # # 2D* 29* #

9 2E 2F& ! * !

! * 71 72& @ * #

" = !

! & #* # #

*

! ! &

! #

#*

5 # * #

Confidential: For Review Only

& ,

! # # A ! &

#5 5

28 # 5 = 5

! * # ; * * # #< "

; * # = <& / >

! # ! #

5 * # !

;. <&

!

" # $ $%&

; < 5 # 5

; < # 712: [77]&

! #

& # !

! 28 # # 7120G2:&

! # ! 7FE*1E1 &

H ! 2111 # 287 . I 7

. ! ! H &

! 271*228 * ! 2DJ E:J

;7*E08 <& # * ! >

08J # :FJ * # * # . & 5

! ! !

[77]& # # # ,

Confidential: For Review Only

3 ;,3< ! [77]& - K ; -<

! #* ! L #

&

% $ $

% ! ! ! "5 %

; % < [70]* 5 ! &

K 20 # !

; #* MN1&F1 & ! # " 2:

85 8 B C 2 B C [7:]&

2:591 ! # K &

= # ! !

& = &

" = ! #

" 4 ; < " 4 # "

; < " 4 " L # ; < - 4

& " * ! B C

B C& #* # = ! ; < "

" 4 " 9 # ; < 4 8

; < " ! # 4 " ! # 9 # &

* !

1N * 2N # * 7 N # ! 0 N & # D1O

8 # ! B # # C B # # &C

! ! [78 7D]* = #

= ! ! & ! "

! "* # 20J # # # ! "&

Confidential: For Review Only

! ! " ! " #

* * & > !

BP9 G #C* B 85D G #C* B 0 5: G #C* B 7 G #C

BQ2 G #C& ! " ! " #

#& * !

B C* G #C* B 2 G #C B7 G #C& L #

! B # #C* B # C* B # C

B 9 # C& # ! ! / ! #G)

R * E5 # 79&

# = ;# G <&

! # 7E* ! ! B !

C ! # &

#* L !

! ! = # !

9& # ! 5 # K ! 7112 ,3

* # L & =

% ; % < ! 7F&

% ! # L

4 2 7 ; <* 0 ; <* : 8 ; !<& !

! ! L &

$ "

> > * 2: ;

- * - * S<& @ ! * !

! # ; ?1&112< * # ! #

& ! " # ! " !

Confidential: For Review Only

3 5 ;35 < & ! ! #

5

& 7 ! = #

& > # ! !

; * # * * * # *

< ! ;% 2<&

! # ! #* L

% ! ! " ;%

7<& 0* = # ! #

! ! >

= # & . ! & % !

L # I ! #

& ; --<*

. * !

! ! > % * # &

* 89*280 # ;:9&E7J< D7*FD7 ;87&2EJ< #

; 2<& / * # ! &

; --< ! ! 1&107

1&17: # > * !

. &

2 ! !

= # ! # & $ "

* # * # # &

; E < ! #

&

Confidential: For Review Only

$ '(& $) * + # $,& $ $ " $ $ "- " #

	$		"		.	
			$ / 0 1 2		$ / 0 1 2	3 $
	'45-"6	67-'68	97(6:2	;4-<;4	64(942	
$	229*E:7	8D*087	:9&E7J	D2*:F1	87&2EJ	?1&1112
% ; <			81 ;E&D1<		:8 ;F&DD<	
$	92*200	07*82D	:8&92J	0ED29	8:&7FJ	?1&1112
		29*200	87&DF	2FF07	82&D2	
/		21*9E	00&2	27281	02&:I	
!		0*121	F&7D	0*9E8	F&E	
		2*8F:	:&EI	7*98:	9&2	
$ " $ # !$	228F2I	88*7E	:9&91J	D1*D7	87&01:	?1&111
		27*1E2	72&E8	22*F82	2F&92	
/		71*7E	0D&I	71*0E	00&D	
!		2D*090	7F&D2	2F*1D:	02&::	
		D*8:E	22&E	F*77	28&7	
& # /=: 0	229*82I	8D* 71	:9&E0J	D201	87&29	?1&111
9 #		0D9I	D&8	DF7	22&7	
#		22182	2F&D	2D18	7D&2	
#		71* 272	08&E	71F7E	0:&2:	
# #		72* 0D	0E	29:1D	7E&0	
" #	229*9::	8D*01F	:9&E7J	D2*:08	87&2EJ	?1&1112
		:8*F8F	E2&D	:8*1F:	90&:	
#		21*081	2E&0E	2D*0:2	7D&D1	
3 " $ $ "	22E:81	8D*D9:	:9&E8	D299D	87&28	?1&1112
# #		:E*297	E8	::*0:E	92&9F	
		E*817	28	29*:7E	7E&72	
%	22E*E:8	8D*EF	:9&E	D2*F:(	87&20	?1&111
7 G #		0*D1F	D&0:	0*:DF	8&D	
Q2 G #		F27	2&	F7F	2&	
0 5: G #		2E*D72	07&90	29*022	79&F8	
8 5D G #		29*2F	01&7	29*E8	7E&E	
P9 G #		2D*88F	7F&2	770E9	0D&2:	
$ #	22E*2:1	8D*82	:9&E	D2*D7	87&2	?1&111
		2:*79E	78&7D	F*E98	2D&17	
G #		29*897	02&1F	28*0D0	7:&F0	
2 G #		27*818	77&2	20*::7	72&E	

!
" # ! $! %
& ' (( ) % ! # (! ! !)) # ! $(! !) ')!! *

Confidential: For Review Only

= # % ! # ! ! =

; 7<& = ! * #

* # * # # = & *

* ! !

! ! = &

$ 4 $ $ $ $ " * * + # $,& $ $ " $ $ - " #

	"		.	
		<6 2		<6 2
$				
	(		(	
/	51&0:'	T51&8D*51&20U	52&:E"	T52&91*52&79U
!	57&2F"	T57&D1*52&9EU	5:&FD"	T58&08*5:&89U
	50&8D"	T5:&2D*57&F8U	58&EF"	T5D&:9*58&01U
$ " $ # !$				
	(		(	
/	52&D8"	T52&EE*52&:0U	57&D0"	T57&F2*57&0:U
!	50&0D"	T50&D1*50&27U	5:&E8"	T58&20*5:&8EU
	58&11"	T58&0F*5:&D2U	5D&:F"	T5D&E0*5D&2:U
& # /=: 0				
9 #	(		(	
#	7&FF"	T7&82*0&:9U	:&92"	T:&0F*8&1:U
#	8&8F"	T8&28*D&10U	E&0F"	TE&1D*E&97U
# #	9&:F"	T9&19*9&E9U	21&91"	T21&08*22&1DU
" #				
	(		(	
	5:&91"	T50&8F*50&02U	58&E:"	T58&78*5:&F1U
3 " $ $ "				
# #	(		(	
	50&E8"	T5:&22*50&D2U	57&99"	T50&1:*57&82U
%				
7 G #	(		(	
Q2 G #	1&19	T51&8F* 1&90U	1&1E	T51&DF* 1&E8U
0 5: G #	52&77"	T52&D1*51&E:U	52&79"	T52&92*51&E0U
85D G #	57&29"	T57&82*52&E0U	50&1D"	T50&87*57&D1U
VN 9 G #	50&97"	T5:&27*50&07U	58&0E"	T58&E1*5:&F9U
$ #				
	(		(	
G #	2&D2"	T2&:2*2&E7U	7&29"	T2&EE*7&:DU

+

Confidential: For Review Only

2 G #	7&78"	T7&1D*7&::U	7&8:"	T7&7D*7&E7U
P7 G #	7&79'"	T7&12*7&87U	7&08"	T7&18*7&DDU
	(		(	
	1&89"	T1&02*1&E2U	1&08"	T1&21*1&8FU
.	2&18"	T1&E9*2&72U	2&2D"	T1&F7*2&:1U
"				
	(		(	
% =	1&2E	T51&7F*1&D8U	51&::'	T51&E7*51&1DU
	51&8:'	T51&E8*51&70U	1&FE"	T1&8F*2&0EU
"	1&F2"	T1&:7*2&0FU	2&21"	T1&D9*2&87U
/	51&:8'	T51&E:*51&1:U	1&01	T51&1F*1&91U

---

[2], > " . # . * % ! ! ! * R
&

" ?1&112* ' ?1&18

> ; 0<* ! !

! # 5

& # # * = #

! ! ! = * !

&

Confidential: For Review Only

,

$ 8 . + $) &$ $ "+$ $) "+$ % % #) * + # $,& $ $ " $ $

	"				.			
	4		8		4		8	
>$ $	F8 J -		F8 J -		F8 J -		F8 J -	
$								
	(		(		(		(	
/	51&:1"	T51&D51&2E	51&2	T51&0D*1&	52&82"	T52&9052&01	51&99"	T51&F51&89
!	57&2D"	T57&8952&98	52&18"	T52&:751&D9	5:&E:"	T58&75:&:DU	57&D9"	T50&1257&00
	50&81"	T5:&2257&EE	52&D0"	T57&2E52&1F	58&E1"	T5D&058&70	57&9F"	T50&0857&7:
$ # !$								
	(		(		(		(	
/	52&D0"	T52&E52&:2	51&EF"	T52&2E51&D:	57&D0"	T57&F57&0:	52&09"	T52&D52&1D
!	50&02"	T50&88*50&1EU	52&E:"	T57&2:*52&8:U	5:&E:"	T58&27*5:&88U	57&7F"	T57&D:*52&FDU
	5:&F8"	T58&0:*5:&8DU	57&::"	T57&E7*57&1DU	5D&:D"	T5D&9F*5D&21U	57&D2"	T50&1:*57&2EU
& # /=: 0								
9 #	(		(		(		(	
#	7&FE"	T7&81*0&:8U	7&DF"	T7&21*0&7EU	:&DF"	T:&0D*8&12U	0&92"	T0&0:*:&1EU
#	8&88"	T8&22*8&FFU	:&01"	T0&9E*:&E7U	E&08"	TE&12*E&DFU	D&D:"	TD&7E*9&11U
# #	9&:8"	T9&10*9&EDU	8&9F"	T8&7F*D&7EU	21&D8"	T21&7F*22&12U	E&2D"	T9&97*E&D1U
" #								
	(		(		(		(	
	5:&D:"	T5:&ED*5:&:7U	50&9E"	T5:&1F*50&:EU	58&99"	T58&FE*58&8DU	5:&12"	T5:&70*50&9EU
3 " $ $ "								
# #	(		(		(		(	
	50&9E"	T5:&17*50&88U	57&D0"	T57&F8*57&01U	57&99"	T50&1:* 57&81U	52&91"	T57&12*52&0FU
%								
7 G #	(		(		(		(	
Q2 G #	1&1	T51&89*1&9	1&0	T51&8E*2&	1&1	T51&DF*1&	51&0	T52&:F*1&9

-

Confidential: For Review Only

0 5: G #	52&77"	T52&D51&E(	51&D2	T51&FF1&70	52&7D"	T52&9:51&E(	51&8	T52&2:*1&1
8 5D G #	57&28"	T57&8:52&E2	51&E7"	T52&7951&09	50&1:"	T50&:F57&8E	52&72"	T52&951&D9
VN 9 G #	50&D9"	T5:&1D*50&79U	52&71"	T52&D8*51&98U	58&07"	T58&90*5:&F1U	52&E2"	T57&7F*52&00U
$ #								
	(		(		(		(	
G #	2&8F"	T2&0E*28	1&89"	T1&7D*18	7&20"	T2&E:*7&	1&8D"	T1&70*1&
2 G #	7&7:"	T7&1:*7&:0U	2&1:"	T1&9F*2&7FU	7&82"	T7&7:*7&9EU	1&D1"	T1&7D*1&F0U
P7 G #	7&7E"	T7&18*7&8:U	1&F1"	T1&88*2&78U	7&0:"	T7&10*7&D:U	1&00'	T1&17*1&D:U
	5!	5!	(		5!	5!	(	
	55	55	51&27	T51&0E*1&2:U	55	55	1&2D	T51&2D*1&:FU
.	55	55	1&29	T51&1D*1&:1U	55	55	1&0D'	T1&1D*1&DDU
"								
	5!	5!	(		5!	5!	(	
% =	55	55	51&1D	T1&E2*51&8FU	55	55	1&::	T51&1E*1&FDU
	55	55	51&1F	T1&E2*51&E:U	55	55	1&08	T51&80*2&70U
"	55	55	1&FF'	T1&12*1&78U	55	55	2&98"	T2&71*7&02U
/	5!	5!	51&8	T1&152&1F	5!	5!	51&2	T51&EE*1&
) ? $ "								
/	5!	5!			5!	5!		
	5!	5!	1&7	T51&10*1&8	5!	5!	2&7D"	T1&FF*28

> #* L # =
) # >
4 % ! ! ! * R &

" ?1&112* ' ?1&18

Confidential: For Review Only

! ! ! # #

5 & # !

! ! ! 5

01 # # > ;@ 2<& "

! & !

! &

" !

! * # # # =

! # # ! "

. & # ! # !

! ! ! " # #

I # I = #I

= & ! >

* #* L #& 5

! ! !

* # & "

*

! ! &

! ! # * ! . # *

! # # &

!

* ! &

! * ! 01 02 *

; < 7128* !

Confidential: For Review Only

! [07]& ! #

[02 00 0:]& $ "#

" * * # * !

#* * # ! " #

! ! - [00]&

! ! ! "

! ! # !

; K < # ! "

; " * # < [08 0D]& /

! ! ! ! *

! [9]& !

! # & . L

! ! [29 2F]* ! =

&

! ! ! *

[09] H ! ! # & #

" > * #

& / ! # * #

! # &

" # !

= * ! ,3 [0E]& ! *

L #& >

* "

#&

Confidential: For Review Only

* # # #

& = # !

=# ! 5 !

& #* ! !

! = ! ! "*

! ! # &

/ > & / ! 5

&

#* ! ! # !

L * &

* ! * ! ;! #<

"# # * #

& ! 5 = 5

& # =

& # H =#

" I

L > #

& I #

&

/ # * * #

!

#* # &

Confidential: For Review Only

! ! ! * H ! & @ ! "

= ! ! " (

$ $ $ " @ * & A

K ! # # = &

3 # ! &

@ ! = ! ! > 5

&

$ " $ A

@ # # * # # 5

! # &

# #

! &

! " !

A

Confidential: For Review Only

B #

#

(# (& ! =

; < # *

(* A * $

&

))

$ W ! # " # & ()

> * # * !

&

@ * # %

! .

7129 - # , # - . &

Confidential: For Review Only

)

2& $-* !# %* + * & / 4 .

! & 712DI0E9;2110D<47:7059E& 4 21&212DG 12:15D90D;2D<1189F5

T / @ 4 712DG18G2:U

7& (- - & - ((# - - I 7129 T 4

4GG!!!& ! & G G21D70F1 D 7129&

0& /= , # & /= #& /= 4 /= , # 712D&

:& X " " Y (& 5 & 4 5 * - @* @ Z * &* & "

- 5 4 * % $ & 4

712:4292050E&

8& +3* (* . [* & % 4 / ! #

(! & 712DI8F;:* <4 :F5 D1& 4

4GG & G21&212DG>&> &712D&1D&171

D& 3 # -.& 4 # #

& 7119ID7;7<4F8521E& 4 21&2109G111051DD=&D7&7&

T / @ 4 7119G17G7EU

9& H# # " 3* ! .& -# # ! 5 4 5 5

#& 7129I2;2<42F57D& 4 21&212DG 70875

:D:7;29<0112252

E& * * % -* & @ # # ! 5

4 @ # & ! " ! #

7129IE1; -<4218528& 4 4GG & G21&212DG>& # &7129&1D&1D:

F& (> * % $ (* (& # ! 5

& 2FF7I72;:<4:925E8& 4 21&2119G 12809EFE

Confidential: For Review Only

21& $ \ @+* % $* * & 5 -

& 4 5 * - @* @ Z * &* & " - 5 4

* % $ & 4 712:427825

018&

22& # $* % * $ * & - " 4 # !

5 # & $% & $ % 712:I0:E 4 21&220DG >& 2282

27& @ * # & ! > ! * !

! 4 5 #& ' (

7127I0:;:<4827577& 4 21&21F0G G 177

20& 5 ! * % * . * & * = # #

4 5 # & (

(! 7121I8479& 4 21&22EDG29:958F9=58579 T /

@ 4 7121G22G27U

2:& - * " 3.* - -* & .]

! 5 # 4 H & ')*

7129I27;7<4 129271D& 4 21&2092G> & &129271D

28& . .& " ^

' (* 7119I21;:<4:7757E& 4 21&2129G 20DEFE111978E881 T / @ 4

1:G12U

2D& , % * /! -$* - " $* & ! # #* #

! & ' '

7119I:7;21<4E8258D& 4 21&2119G 112795119517075=

29& @ +* ! # -& #) A * * # 5 &

" 711DI2D;0<408059E& 4 21&2222G>&2807599F8&711D&11:FE&=

+

Confidential: For Review Only

2E& %* * . * & # !

! & ' ! ! % 712DIE9; -<4D521& 4

4GG & G21&212DG>&# &712D&17&172

2F& * * * & . # ! 5 &

7121I00;2<42905ED& 4GG & G21&212DG>& &711F&10&120

71& .* . & - 5 4 # (! . & + "

7110ID2;2<48F59E& 4 21&2170G 421727E:728E12

72& (* + * * & % % 4 # (!

& , 7129I7D;F<470:F5D7& 4 21&2119G 21E7D51295

198:51

77& - - & /, ^ #& 4 ,3 *

712D&

70& (* .* @ ! " (* & ! "5 % 5 ; % <4

,3 & -) 7119I84D05D0& 4

21&22EDG2:9959878585D0

7:& @* ! 5 ! * " +& ! "5 % 5 ; % <

4 I 7128 T 4

4GG!!!& & G G G7D9E 95

% J71, J71$ J71) J717J71% #J717128& 2F / 7129&

78& % 3* * . * & # # #

2252D # 4 % # 7120 5 -

; -< # & $% ' (712DI2D48DF& 4 21&22EDG 27EEF512D507205E

7D& %.* /" # * - # * & # # # # L

/ ; -< #4 #& $.

7112I08;:<47D059& T / @ 4 7112G19G02U

,

Confidential: For Review Only

79& / ! & (/ ! #G) R & , # 4 (-

; % . - < 2FFD&

7E& - " -(* 3(* $ $* & # H

4 5 # & ' - 7121I78;7<4D85E0& 4

21&2109G 11712:F

7F& - . $ & . 7128

T 4

4GG!!!& & "G G G # G G _ G G:D89F2G _

_ _ _7128 _5 _ _(&

01& / & % 5 4 A

! 5 & * 7128&

02& / & A ! 5 4 / I 7129

T 4

4GG!!!& & & "G #G ! G G# !

G7129 21 7129&

07& @ * % & # & . * 7128&

00& - -* $ (* # +& 5 - ; -< # 4

" * % # % # 711FG21 #& 4 - (, 7121&

0:& " @* % +* 3 * & 5 - ; -< 71205712:

712: T 4 !!!& & &

08& " -.* " ! +* " * & 5 ` 5(

4@ @ , #* ,3 . #&

712:I0:;:<482E50E& 4 21&2299G1797:02D208121E7

0D& " -.* " ! +* 3 # +* & % , * * 5 4

- 5 @ @ ,3 . #& ' (

7128I218;2<429059F& 4 21&7218G + &7120&0129E0

-

Confidential: For Review Only

09& - - * - [* # % * & ! 5

& 711FG7121 #& 5

; -< #& "4 / (/ * 71274787&

0E& # * @ H & - % - 4 # ^

(@ ,3 % - #&

' 712DI88;F<49925E0& 4 21&212DG>&> &712D&18&12F

Confidential: For Review Only

FIGURE 1

Relationship between wellbeing and health behaviours: (A) Risk Factors (B) Protective Factors

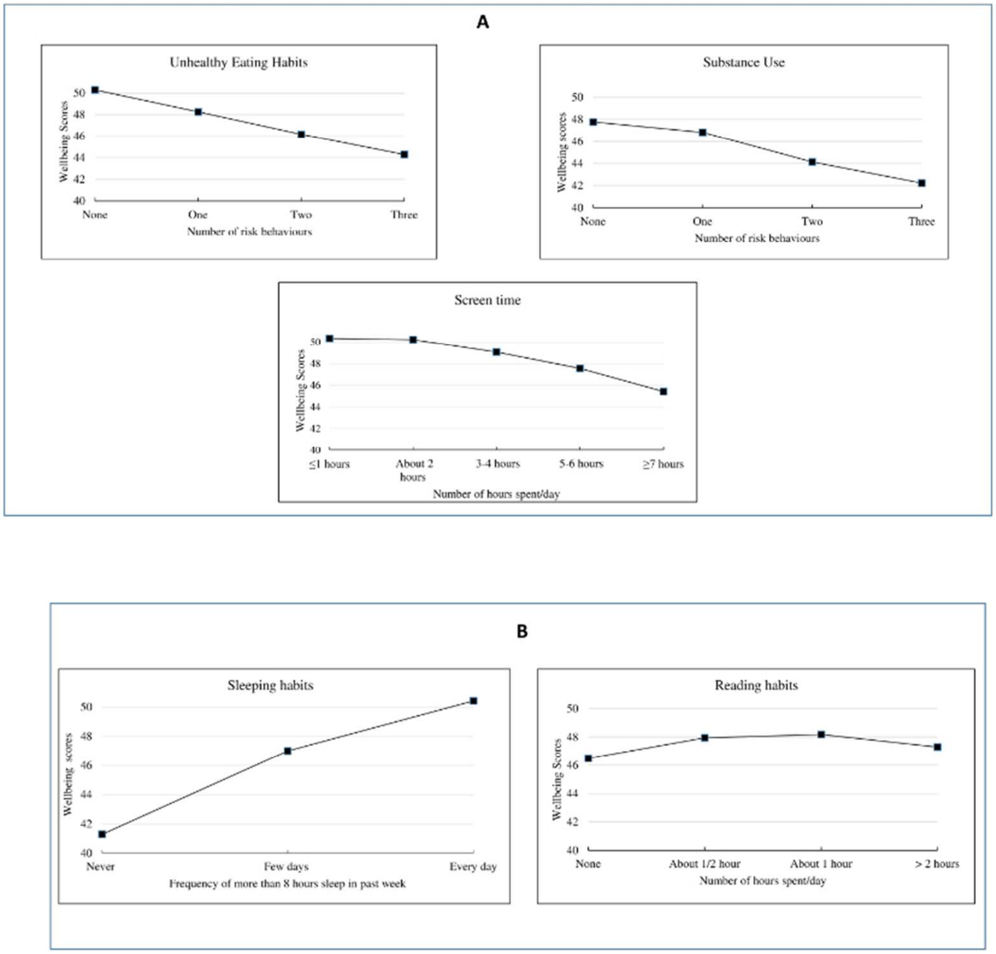

ew Only

APPENDIX A

(i) VARIABLE DESCRIPTION

Composite variables	Original variables	Variable labels	Composite variables
Substance use risk[1-6]	Definitions were		Alcohol + smoking + cannabis
	Smoking: Cigarette smoking status (currently smoke)	SmkSt2	(1) Smoking risk: If currently smokes (2) Drinking risk: If drinks once a month or more frequently (3) Cannabis risk: If ever tried cannabis. Each item was coded 0 for "no" and 1 for "yes". The composite score obtained ranged from zero to three based on the number of risk behaviours; (0= None, 1= Only one, 2 =Any two and 3 =All three
	Drinking: Alcohol drinking status (Usually have an alcoholic drink once a month or more frequently)	alcfrq1	
	Cannabis: Cannabis use status (Ever tried cannabis)	Cantry	
Physical Activity [7,8]	Physical activity: in line with general recommendations		Duration of exercise + Frequency of exercise + Number of days met recommended guidelines
	How many days physically active for 60+ minutes, in last 7 days (Active for more than 5 days)	Phyact	The first question on moderate to vigorous physical activity was "Over th past 7 days, on how many days you were physically active for a total of a least 60 minutes per day and responses ranged from 0 to 7. Second, the participants were asked "Outside school hours: how often do you usually exercise in your free time so much that you get out of breath or sweat?" with responses from less than a once a month to every day. The questio on duration was "Outside school hours: how many hours do you usually exercise in your free time so much that you get out of breath or sweat?", with responses from none to 7 or more hours. Adolescents who exercised for more than twice a week for an hour or more or were physically active for 60+ minutes for at least 5 days were classified as "physically active" and the rest "physically inactive".
	How often usually exercise in free time (Active for mc than twice a week)	ExerOft	
	How many hours usually exercise in free (Active for hour or more)	ExerHrs	
Sleeping habits[9,10]	Sleeping frequency in last week		
	How often slept 8+ hours per night in last 7 days	Sleep8pn	The question was "Over the past 7 days, how often did you sleep for hours or more" and the responses were "every day", "most days", "some days" and "not in the past 7 days".

Confidential: For Review Only

Eating Habits [11-13]	Definition of each unhealthy eating habits were:		Breakfast consumption + take away food + 5 Aday
	How often eaten takeaway food in last 7 days (unhealthy habit if taken takeaway in past seven days	Eattake	(1) Skipping Breakfast: If avoided breakfast in last 7 days (2) Poor die consumed less than 5 portions of fruits and vegetables a day (3) Takeaw food: If consumed takeaway food in past 7 days. Each item was coded 0 for "no" and 1 for "yes". According to the number of unhealthy eating habits participant's exhibited, a risk score from zero to three (0= None, 1= Only one, 2 =Any two and 3 =All three) was given
	How often eaten breakfast in last 7 days (unhealthy h if breakfast not taken regularly)	Eatbfst	
	Consumed more than five portions of fruit and veg yesterday (unhealthy habit if 5-A-DAY not taken regularly)	FV5ormor	
Screen time [8 14-16]	Definitions of screentime derived as follows (Weekend and weekday hours combined)		TV +_computer games + internet
	Hours per day watching TV	Watchwk, Watchwe	FOR ALL, Response options for each item were 'none at all', 'about h an hour a day', 'about 1 hour a day', 'about 2 hours a day', 'about 3 hours a day', 'about 4 hours a day', 'about 5 hours a day', 'about 6 hours a day' and 'about 7 or more hours a day'. The total screen time was calculated by grouping the similar options of each item and by combining both Z F H N H Q G V D Q G Z H H N G D \ V 6 X E M H F W V Z H U "About 5-6 hours/day", "About 3 -4 hours/day", "About 2 hrs/day" and 3" K Day". G
	Hours per day spent playing computer games	Compwk, Compwe	
	Hours per day spent on computers for communicatin or homework	Comphwk, Comphwe	
	Hoursper day spent on smart phone	Smartwk, Smartwe	
Reading [17]	Definitions of reading derived as follows:		Reading hours (weekend + weekdays)
	Hours per day spent reading outside school hours	Readwk, Readwe	"About how many hours a day do you usually spend sitting down read books, magazines or newspapers (including e-readers, online newspapers, and magazines) and studying when you are not at school?". The question were asked for weekends and weekdays separately. The response optio U D Q J H G I U R P Q R Q H W R K R X U V S H U G
Bullying [18 19]	Frequency with which the behaviour occurred over the past couple of months:		Traditional bullying (physical+ verbal + relational)

Confidential: For Review Only

I was called mean names, was made fun of, or tease a hurtful way	Bllynam	Bullying measure was meant to assess the extent to which they had recently experienced other children directing different types of bullying towards themselves. Six statements reflecting traditional bullying (physical, verbal, and relational) were asked. Questions were rated by participants using a 5-point response scale that ranged from 0="I haven't been bullied this way in the past couple of months" 1="It has happened once or twice", 2="2 or 3 times a month", 3="2 or 3 times a week", to 4="Several times a week."
Other people left me out of things on purpose, excluc me from their group of friends, or completely ignored me	Bllyout	
I was hit, kicked, pushed, shoved around, or locked indoors"	Bllyhit	
Other people lied or spread humours	Bllylie	
Made fun of me because of weight	Bllyfun	
Sexual jokes/comments	Bllysex	

(ii) Individual Items and scoring of Warwick-Edinburgh Mental Well-being scale (WEMWBS)

1. I've been feeling optimistic about the future
2. I've been feeling useful
3. I've been feeling relaxed
4. I've been feeling interested in other people
5. I've had energy to spare
6. I've been dealing with problems well
7. I've been thinking clearly
8. I've been feeling good about myself
9. I've been feeling close to other people
10. I've been feeling confident
11. I've been able to make up my own mind about things
12. I've been feeling loved
13. I've been interested in new things
14. I've been feeling cheerful

Each of the 14 item responses in WEMWBS are scored from 1 (none of the time), 2 (rarely), 3 (some of the time), 4 (often) to 5 (all of the time). A total scale score is calculated by summing the 14 individual item scores.

Confidential: For Review Only

REFERENCES

1. Farmer S, Hanratty B. The relationship between subjective wellbeing, low income and substance use among schoolchildren in the north west of England: a crosssectional study. Journal of Public Health 2012;34(4):512-22. doi: 10.1093/pubmed/fds022
2. Phillips-Howard PA, Bellis MA, Briant LB, et al. Wellbeing, alcohol use and sexual activity in young teenagers: findings from a cross-sectional survey in school children in North West England. Substance abuse treatment, prevention, and policy 2010;5:27. doi: 10.1186/1747-597x-5-27 [published Online First: 2010/11/12]
3. Newcomb MD, Bentler PM, Collins C. Alcohol Use and Dissatisfaction with Self and Life: A Longitudinal Analysis of Young Adults. Journal of Drug Issues 1986;16(4):479-94. doi: 10.1177/002204268601600401
4. Bergman MM, Scott J. Young adolescents' wellbeing and health-risk behaviours: gender and socio-economic differences. Journal of Adolescence 2001;24(2):183-97. doi: https://doi.org/10.1006/jado.2001.0378
5. Taylor G, McNeill A, Girling A, et al. Change in mental health after smoking cessation: systematic review and meta-analysis. BMJ : British Medical Journal 2014;348 doi: 10.1136/bmj.g1151
6. Booker CL, Skew AJ, Sacker A, et al. Well-Being in Adolescence—An Association With Health-Related Behaviors:Findings From Understanding Society, the UK Household Longitudinal Study. The Journal of Early Adolescence 2014;34(4):518-38. doi: 10.1177/0272431613501082
7. Ussher MH, Owen CG, Cook DG, et al. The relationship between physical activity, sedentary behaviour and psychological wellbeing among adolescents. Social Psychiatry and Psychiatric Epidemiology 2007;42(10):851-56. doi: 10.1007/s00127-007-0232-x
8. Booker CL, Skew AJ, Kelly YJ, et al. Media Use, Sports Participation, and Well-Being in Adolescence: Cross-Sectional Findings From the UK Household Longitudinal Study. American Journal of Public Health 2015;105(1):173-79. doi: 10.2105/AJPH.2013.301783
9. Fuligni AJ, Hardway C. Daily Variation in Adolescents' Sleep, Activities, and Psychological Well-Being. Journal of Research on Adolescence 2006;16(3):353-78. doi: 10.1111/j.1532-7795.2006.00498.x
10. James S, Hale L. Sleep Duration and Child Wellbeing: A Nonlinear Association. Journal of clinical child and adolescent psychology : the official journal for the Society of Clinical Child and Adolescent Psychology, American Psychological Association, Division 53 2017;46(2):258-68. doi: 10.1080/15374416.2016.1204920
11. Rooney C, McKinley MC, Woodside JV. The potential role of fruit and vegetables in aspects of psychological wellbeing: a review of the literature and future directions. Proceedings of the Nutrition Society 2013;72(4):420-32. doi: 10.1017/S0029665113003388 [published Online First: 09/11]
12. Conner TS, Brookie KL, Carr AC, et al. Let them eat fruit! The effect of fruit and vegetable consumption on psychological well-being in young adults: A randomized controlled trial. PLoS ONE 2017;12(2):e0171206. doi: 10.1371/journal.pone.0171206
13. Lien L. Is breakfast consumption related to mental distress and academic performance in adolescents? Public Health Nutrition 2007;10(4):422-28. doi: 10.1017/S1368980007258550 [published Online First: 04/01]
14. Hoare E, Milton K, Foster C, et al. The associations between sedentary behaviour and mental health among adolescents: a systematic review. International Journal of Behavioral Nutrition and Physical Activity 2016;13(1):108. doi: 10.1186/s12966-016-0432-4
15. Przybylski AK, Weinstein N. A Large-Scale Test of the Goldilocks Hypothesis:Quantifying the Relations Between Digital-Screen Use and the Mental Well-Being of Adolescents. Psychological Science 2017;28(2):204-15. doi: 10.1177/0956797616678438

Confidential: For Review Only

16. Hamer M, Yates T, Sherar LB, et al. Association of after school sedentary behaviour in adolescence with mental wellbeing in adulthood. Preventive Medicine 2016;87(Supplement C):6-10. doi: https://doi.org/10.1016/j.ypmed.2016.02.021
17. Trainor S, Delfabbro P, Anderson S, et al. Leisure activities and adolescent psychological well-being. Journal of Adolescence 2010;33(1):173-86. doi: https://doi.org/10.1016/j.adolescence.2009.03.013
18. Landstedt E, Persson S. Bullying, cyberbullying, and mental health in young people. Scandinavian journal of public health 2014;42(4):393-9. doi: 10.1177/1403494814525004 [published Online First: 2014/03/13]
19. Przybylski AK, Bowes L. Cyberbullying and adolescent well-being in England: a population-based cross-sectional study. The Lancet Child & Adolescent Health 2017;1(1):19-26. doi: 10.1016/S2352-4642(17)30011-1

Confidential: For Review Only
